# Heavy Heterodendralenes: Structure and Reactivity of Phosphabora[3]dendralenes

**DOI:** 10.1021/jacs.4c07850

**Published:** 2024-08-14

**Authors:** Vesela G. Zarkina, Gary S. Nichol, Michael J. Cowley

**Affiliations:** EaSTCHEM School of Chemistry, University of Edinburgh, Joseph Black Building, David Brewster Road, Edinburgh EH9 3FJ, United Kingdom

## Abstract

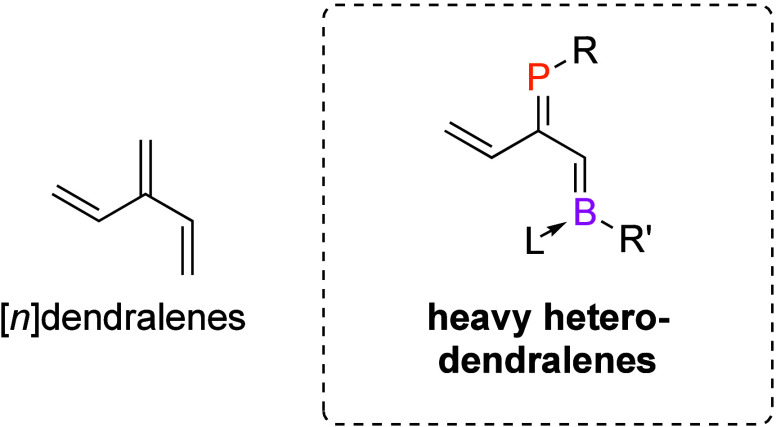

The incorporation of phosphorus and boron into [3]dendralenes provides access to heavy heterodendralenes, a new class of main-group precursor to “doped” polycyclic hydrocarbons. [*n*]Dendralenes are a core class of unsaturated hydrocarbons built from geminally connected polyenes; the resulting arrangement of conjugated C=C bonds enables [*n*]dendralenes to undergo reactions that allow rapid access to complex polycyclic compounds. The increasing technological and synthetic importance of main-group-containing polycyclic hydrocarbons and their analogues makes new routes to access such systems highly attractive. Here we report the preparation of the first heavy heterodendralenes in the form of phosphorus- and boron-containing [3]dendralenes, prepared by a ring-opening reaction of a 1,2-phosphaborete. We reveal the electronic effect of P/B incorporation and demonstrate that, like their hydrocarbon analogues, phosphabora[3]dendralenes undergo diene-transmissive cycloaddition chemistry.

The electronic properties of unsaturated organic compounds can be modulated by placing heavier main-group elements within their π systems. Such incorporation significantly alters the character, energy, and energetic separation of frontier molecular orbitals compared to the “parent” hydrocarbons. The resulting precise and systematic control over the electronic and optical properties of unsaturated organic molecules has established the inclusion of main-group elements as an important tool for designing functional compounds and materials. The technique proves essential for applications in organic electronics, molecular sensing, bioimaging, and supramolecular chemistry.^1−6^

π-Conjugated compounds containing main-group elements within the arene cores are quite numerous. Replacement of a “CH” unit leads us to phosphinines,^7−10^ arsinines,^11,12^ base-supported borabenzenes,^13,14^ or silabenzenes^15−18^ (Figure 1, top). More ambitiously, isoelectronic replacement of two-carbon “HC=CH” units with “B/N” or “P/B” generates borazines,^19,20^ azaborines,^21,22^ or phosphaborines.^23−25^ Polycyclic aromatic hydrocarbons containing boron,^26−30^ nitrogen, and/or phosphorus are a particularly active area of research due to potential applications in materials science.^31−33^

**Figure 1:**
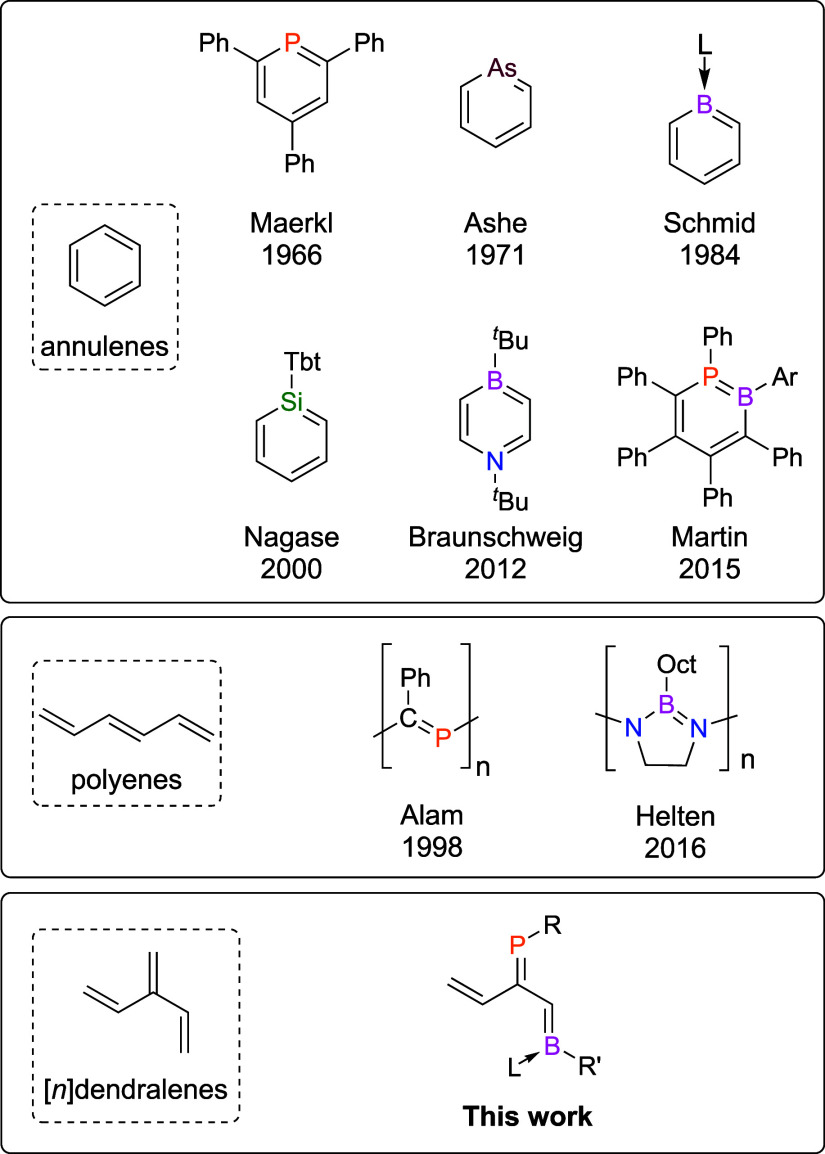
(top) Selected main-group isosteres of arenes. L = pyridine. (middle) Selected main-group isosteres of polyenes. (bottom) [3]Dendralene and phosphabora[3]dendralene.

Despite the focus on the main-group modification of arenes, they are only one of several core classes of unsaturated hydrocarbons. Two other major classes stand out: linear polyenes and [*n*]dendralenes, which are cross-conjugated^34,35^ polyenes with geminal connectivity.^36^ Main-group-element-containing examples of linear polyenes include systems with conjugated C=P^37−39^ or B=N bonds (Figure 1, middle).^40,41^ Of the [*n*]dendralenes, which have only recently gained significant attention as starting materials in organic chemistry,^42−45^ there are no heavier main-group-element-containing examples (Figure 1, bottom).

Interest in [*n*]dendralenes is largely motivated by their role as powerful precursors of polycyclic compounds. Bearing multiple diene units allows [*n*]dendralenes to undergo sequential diene-transmissive pericyclic reactions, and cascades of such reactions allow for shorter synthetic routes to polycyclic systems.^46−49^ If capable of similar reactivity, main-group-element-containing [*n*]dendralenes would provide a powerful synthetic point of entry into an array of electronically tunable main-group-element-containing polycyclic compounds of interest as precursors for optoelectronically active polycyclic aromatic compounds.

Here we report the preparation of the first heavy heterodendralenes by isoelectronic replacement of two carbon atoms with a phosphorus/boron pair. We were motivated by our interest in “phosphaboraorganic” compounds,^50^ in which C=C units are isoelectronically substituted with P/B pairs. Such substitution is emerging as a powerful potential tool for the precise design of functional compounds and materials. P/B incorporation leads to changes in the structure and UV/vis spectra compared to [3]dendralene. We demonstrate that like the parent [3]dendralene, phosphabora[3]dendralenes participate in diene-transmissive Diels–Alder reactions, opening synthetic access to polycyclic systems.

Our strategy to access phosphabora[3]dendralenes was based on ring-opening reactivity of 1,2-phosphaboretes that we previously pioneered.^50^ Accordingly, we prepared phosphaborete **1** from the reaction of diphosphadiboretane **I** with 1-ethynylcyclohexene (Scheme 1), with the compound obtained in 66% yield. The ^31^P{^1^H} and ^11^B NMR chemical shifts of **1** (δ −45.2 and 43.7, respectively) are as expected for a 1,2-phosphaborete. Single-crystal X-ray crystallography studies further verified the identity of **1** (see the Supporting Information (SI)). The addition of the alkyne is entirely regioselective; **1** is the sole observed product of this reaction, which likely proceeds through monomerization of **I** to generate a transient phosphaborene intermediate.^48,51^

**Scheme 1:**
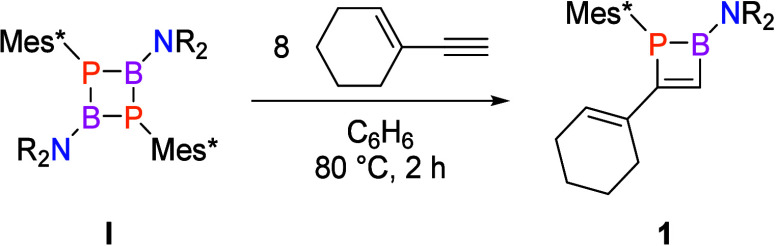
Preparation of 1,2-Phosphaborete **1** (Mes* = 1,3,5-Tri-*tert*-butylphenyl; NR_2_ = 2,2,6,6-Tetramethylpiperidine)

Phosphabora[3]dendralenes are readily obtained by base-induced ring opening of **1**. When we combined 1,3,4,5-tetramethylimidazol-2-ylidene^52^ (^Me^IMe) and **1** in benzene at room temperature, we observed the immediate precipitation of deep-red crystals of **2** (Scheme 2). The emergence of a new resonance in the ^31^P{^1^H} NMR spectrum at δ 127.5 accompanied the complete consumption of **1**. The new downfield ^31^P resonance (vs δ −45.2 in **1**), together with a low-field doublet in the ^13^C{^1^H} NMR spectrum (δ 191.15, ^1^*J*_C–P_ = 56.1 Hz), confirmed the formation of a Mes*P=C phosphaalkene moiety.

**Scheme 2:**
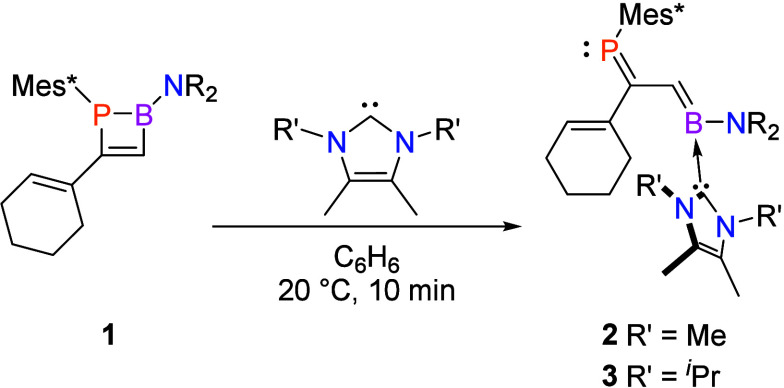
Preparation of Phosphabora[3]dendralenes **2** and **3** from **1** (Mes* = 1,3,5-Tri-*tert*-butylphenyl; NR_2_ = 2,2,6,6-Tetramethylpiperidine)

Single-crystal X-ray crystallography revealed the solid-state structure of **2** (Figure 2a). The P1–C2 and B1–C1 bond distances are 1.7361(10) and 1.4854(14) Å, respectively, typical of phospha- and boraalkenes.^53,54^ The cyclohexene double bond (C3–C4, 1.3491(13) Å) remains intact, completing the dendralene core. In the solid state, the P1–C2 and B1–C1 bonds are coplanar; however, we observe a P1–C2–C3–C4 torsion angle of −53.82(11)° relative to that plane. In the solid state, the configuration of **2**’s dendralene core is thus best described as gauche *cis*,*trans* (referring respectively to the P1–C2/C3–C4 and P1–C2/B1–C1 “pairs” of double bonds).

**Figure 2:**
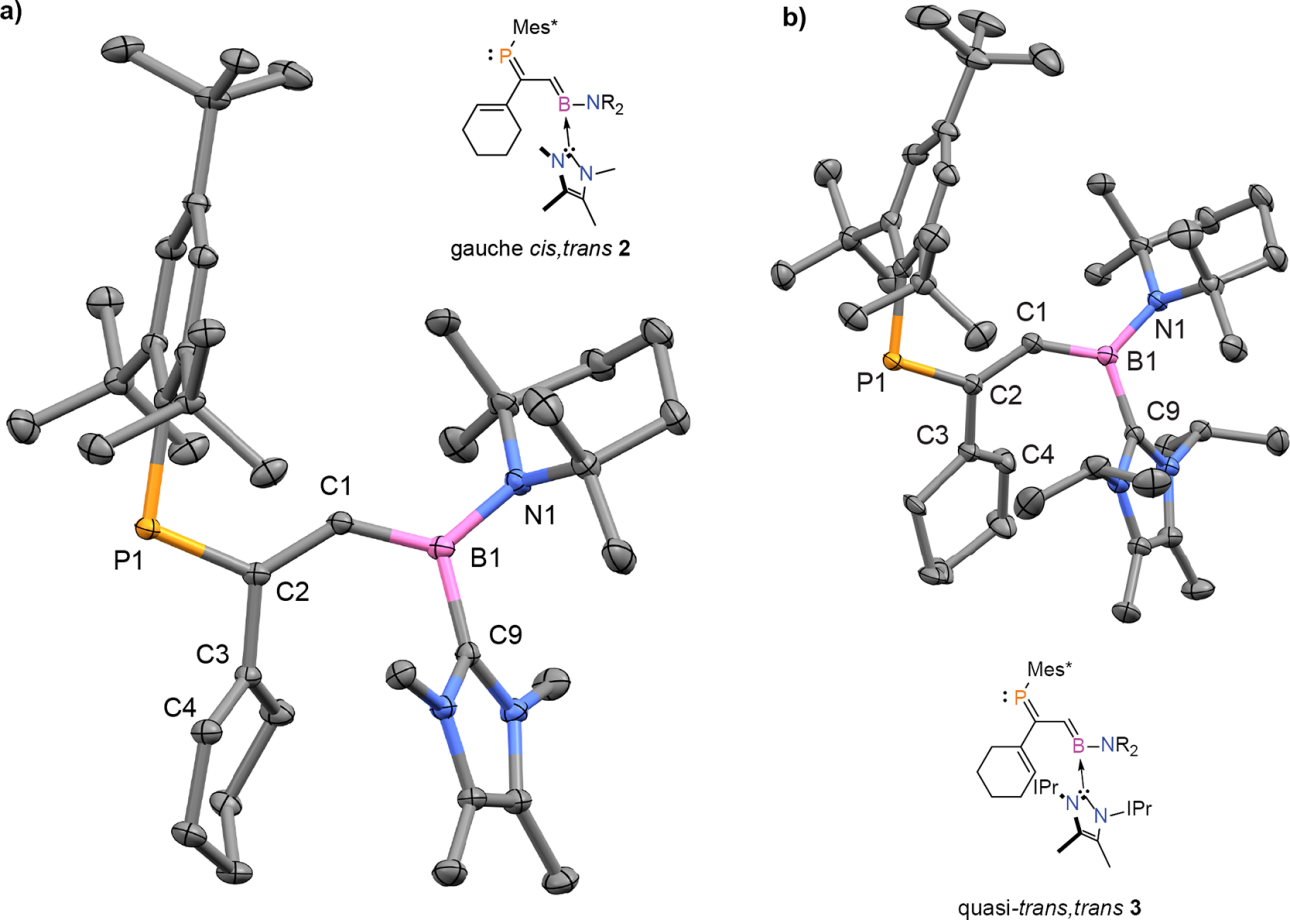
(a) Molecular structure of **2** in the solid state, revealing its gauche *cis*,*trans* conformation. P1–C2 1.7361(10) Å, C1–C2 1.4232(13) Å, B1–C1 1.4854(14) Å, B1–C9 1.6070(14) Å, C3–C4 1.4953(13) Å, B1–N1 1.4925(13) Å, P1–C2–C3–C4 −53.82(11)°. (b) Molecular structure of **3** in the solid state, revealing its quasi-*trans*,*trans* conformation. P1–C2 1.7433(15) Å, C1–C2 1.430(2) Å, B1–C1 1.492(2) Å, B1–C9 1.626(2) Å, C3–C4 1.349(2) Å, B1–N1 1.4926(19) Å, P1–C2–C3–C4 140.26(14)°. Ellipsoids are drawn at 50% probability; hydrogen atoms have been omitted for clarity.

Following the synthesis of **2**, we reacted the larger isopropyl-substituted NHC ^*i*Pr^IMe^52^ and **1** under identical conditions, generating a deep-purple solution. Phosphabora[3]dendralene **3** was obtained from a concentrated hexane solution at room temperature as purple crystals. Similarly to **2**, the ^31^P{^1^H} NMR spectrum of **3** revealed a singlet resonance at δ 137.3 coupled to a carbon atom in the ^13^C{^1^H} NMR spectrum at δ 189.8 (^1^*J*_C–P_ = 55.6 Hz), confirming phosphaalkene formation. X-ray crystallography confirmed the formation of the phosphabora[3]dendralene **3** (Figure 2b). We note briefly here that we could also observe the formation in solution, though not isolate, a DMAP adduct of a phosphabora[3]dendralene (see the SI for details).

Although **2** and **3** possess similar P1–C2, B1–C1, and C3–C4 bond distances, the conformations of their dendralene cores diverge. **3** is appreciably more planar, and with the C3–C4 bond adopting a torsion angle of 140.26(14)° with respect to the PC/CB plane, the compound is best described as adopting a quasi-*trans*,*trans* conformation in the solid state. Akin to their hydrocarbon parent compounds, neither **2** nor **3** thus adopts a planar (i.e. wholly cross-conjugated) structure.^44^

The “central” C1–C2 bonds in **2** and **3** were found to be relatively short (1.4232(13) and 1.430(2) Å, respectively), which indicates some conjugation of the P1–C2 and B1–C1 bonds. This was further supported by density functional theory (DFT) calculations (M06-2X/def2svp) on the optimized geometries of **2** and **3**, which revealed that in both compounds the HOMO–3 has substantial electron density located across the P=C–C=B units (Figure S5). The HOMOs of **2** and **3** have substantial P1–C2 and B1–C1 π-bonding character; the LUMOs are correspondingly largely P1–C2 and B1–C1 π* in nature. **2** and **3** have similar frontier orbital energies and HOMO–LUMO gaps (4.724 eV for **2**, 4.729 eV for **3**).

Regardless of their different colors and conformations in the solid state, UV/vis spectra of **2** and **3** in *n*-pentane reveal almost identical λ_max_ at 500 and 496 nm, respectively (Figures S1 and S3). Time-dependent DFT (TD-DFT) calculations (M06-2X/def2svp) on **2** and **3** duplicate the experimentally observed trends in the spectra, though the absorptions are blue-shifted by ∼60 nm (Figures S6 and S7). The calculations nevertheless suggest that the absorptions at 500 and 496 nm correspond to HOMO–LUMO transitions.

Though **2** and **3** have similar UV–vis absorption spectra in solution, they differ in color in the solid state (**2** is red, **3** is purple), where of course they adopt differing conformations. We used TD-DFT to predict the UV–vis absorption spectra of **2** and **3** in both gauche *cis*,*trans* and quasi-*trans*,*trans* conformations. In both cases, the longest-wavelength absorption is rather insensitive to the conformation (Figures S6–S9). λ_max_ values for **2**/**3** are predicted at 440/458 nm (gauche *cis*,*trans*) and 438/447 nm (quasi-*trans*,*trans*). For both compounds, the two isomers are calculated to be close in energy (2–8 kcal mol^–1^).

NMR spectroscopic studies are more revealing about the conformations adopted by **2** and **3** in solution, since they allow us to estimate the H–H distance between the boraalkene (B=C*H*) and alkene (C=C*H*) units in **2** and **3**. Determination of H···H distances can be achieved using NOESY NMR experiments and DFT calculations; the ratio of NOE integrals for “known” (estimated using DFT) and unknown H···H distances is used to calculate the unknown distance (SI section 4.3).^55^ In the gauche *cis*,*trans* conformation, the DFT-predicted H···H distances between the B=CH and C=CH units in **2** and **3** are 4.663 and 4.695 Å, respectively. However, for the quasi-*trans*,*trans* conformation, the same distances are much shorter, at 3.500 and 3.480 Å (Table S10). For the conformations for which we have crystallographic data, the DFT-predicted distances are a good match for the distances determined experimentally (taking into consideration the X-ray crystallographic underestimation of C–H bond distances). Using ^1^H NOESY NMR experiments, we estimated the B=CH and C=CH distances in **2** and **3** to be 3.091 and 3.682 Å, respectively. This finding is consistent with both compounds adopting the quasi-*trans*,*trans* conformation in solution at room temperature.

The synthetic utility of [*n*]dendralenes lies in their ability to participate in diene-transmissive Diels–Alder transformations.^56^ Therefore, we set out to investigate the reactivity of **2** with the electron-poor dienophile dimethyl acetylenedicarboxylate, which we expected to participate in normal-electron-demand [4 + 2] cycloadditions.

Crucially for Diels–Alder reactivity, conformations of **2** or **3** in which a least one pair of double bonds are mutually *cis* must be energetically accessible. Although our NMR experiments suggest that the favored solution conformation for both **2** and **3** is quasi-*trans*,*trans*, our DFT calculations place this isomer above, though close in energy, to the *cis*,*trans* conformations, especially for **2** (+2.2 kJ mol^–1^ for **2**, +8.2 kJ mol^–1^ for **3**). Although low-temperature ^31^P{^1^H} NMR studies on **2** and **3** (see the SI) did not allow us to observe any splitting of the single room-temperature resonance for **2**, when solutions of **3** were cooled to 213 K we observed its room-temperature singlet to split into two signals. These observations are consistent with two rapidly exchanged conformers of **3** existing in solution at room temperature.

The polycyclic phosphorus-containing compound **4** is formed when **2** is treated with dimethyl acetylenedicarboxylate at room temperature, arising from an apparent [4 + 2] cycloaddition reaction (Scheme 3). Upon addition, the immediate formation of a deep-orange-red solution was observed. The resonances of the P=C (phosphalkene) unit of **2** were no longer visible in the ^31^P{^1^H} or ^13^C{^1^H} NMR spectrum. Instead, we saw the emergence of two new resonances in the ^31^P{^1^H} NMR spectrum at δ 34.4 and 36.2 in a 3:1 ratio. In the ^13^C{^1^H} spectrum, the phosphaalkene P=C resonances were replaced by two signals at δ 131.1 (^1^*J*_C–P_ = 33.1 Hz) and 129.2 (^1^*J*_C–P_ = 36.2 Hz), also in a 3:1 ratio. The ^13^C chemical shifts of these signals indicate that the carbons remain alkenic (as expected in the anticipated cycloaddition reaction), but the reduced values for ^1^*J*_C–P_ in **4** compared to **2** (^1^*J*_C–P_ = 56.1 Hz) indicate the destruction of the P=C π bond. Further evidence for formation of **4** is apparent in the ^1^H NMR spectrum of the product mixture, in which the C=CH resonance of the cyclohexene ring of **2** is replaced by two new resonances at δ 3.86 and 3.74.

**Scheme 3:**
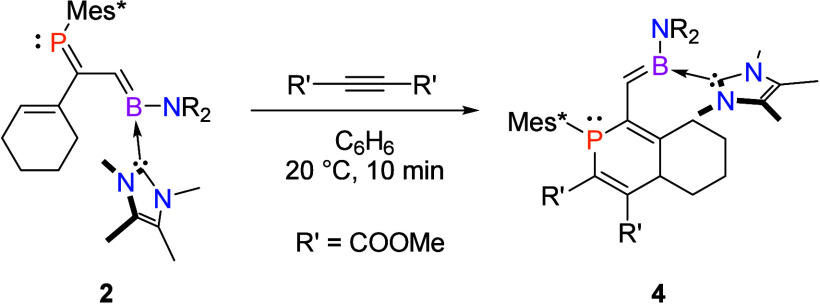
Formation of **4** from **2** and Dimethyl Acetylenedicarboxylate (Mes* = 1,3,5-Tri-*tert*-butylphenyl; NR_2_ = 2,2,6,6-Tetramethylpiperidine)

Single-crystal X-ray crystallography confirmed the structure of **4** to be a product of a diene-transmissive formal [4 + 2] cycloaddition between the 1-phosphabutadiene unit of **2** and the alkyne (Figure 3). The newly formed heterodiene consists of the B1–C10 B=C bond, which is maintained, and the newly formed C1–C2 alkene. P1 adopts typical trigonal-pyramidal geometry, with a sum of bond angles equal to 323.47(8)°.

**Figure 3:**
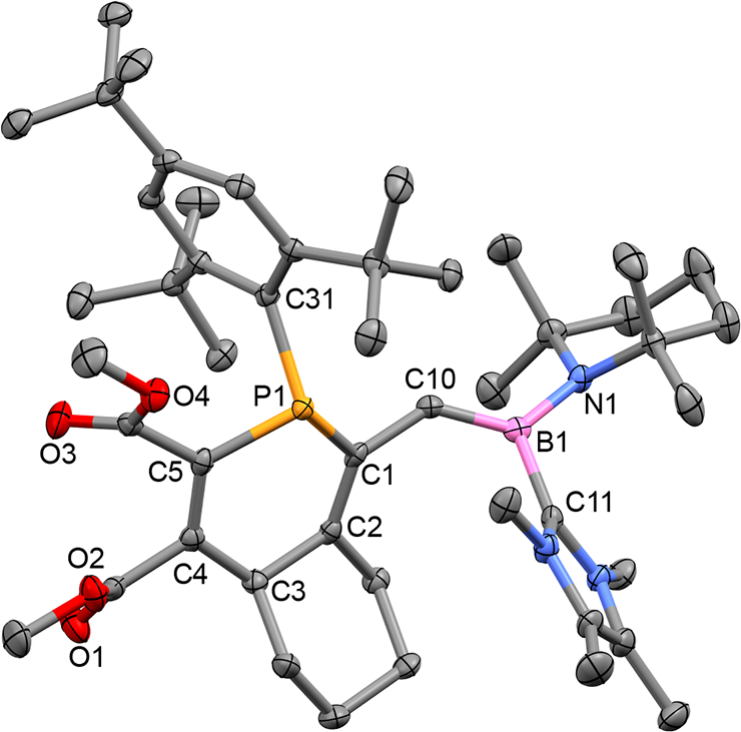
Molecular structure of **4** in the solid state. P1–C31 1.8778(17) Å, P1–C1 1.8395(17) Å, P1–C5 1.8107(18) Å, C1–C2 1.351(2) Å, C2–C3 1.515(2) Å, C4–C5 1.343(2) Å, B1–C10 1.457(3) Å, B1–C11 1.621(2) Å, B1–N1 1.497(2) Å. Ellipsoids are drawn at 50% probability; hydrogen atoms have been omitted for clarity.

The observation of two resonances in a 3:1 ratio in the ^31^P{^1^H} spectrum and others in the same ratio in the ^13^C and ^1^H NMR spectra is attributed to the formation of **4** as a mixture of two diastereomers, arising from the stereogenic phosphorus and carbon (C3) centers. The presence of the Mes* substituent at phosphorus typically lowers barriers to planarization and can allow for facile racemization. In **4**, we observe the onset of line broadening from racemization in the ^31^P{^1^H} NMR spectrum at 360 K. Using DFT, we calculated the barrier to racemization of **4** to be 25.5 kcal mol^–1^, consistent with the observed NMR behaviors. As a result of its relatively facile racemization, the stereochemistry of the observed diastereomers of **4** is not informative with regard to the nature of the [4 + 2] cycloaddition reaction. Reaction of the alkyne with the P=C–C=C unit of **2** is consistent with previously observed reactivity of isolable and transient 1-phosphabutadienes.^57−59^

In conclusion, we have reported the preparation of the first heavy heterodendralenes. P/B “doping” of dendralenes raises frontier molecular orbital energies and narrows HOMO–LUMO gaps compared to hydrocarbon analogues. Like their hydrocarbon analogues, phosphabora[3]dendralenes may engage in diene-transmissive Diels–Alder cycloaddition chemistry, which opens the possibility of synthetic routes to main-group-doped polycyclic compounds, ultimately potentially including polycyclic aromatic species.

## Supporting Information Available

Experimental procedures, characterization, and information on crystallographic and DFT studies (PDF)

## References

[ref1] Hirai M.; Tanaka N.; Sakai M.; Yamaguchi S. Structurally Constrained Boron-, Nitrogen-, Silicon-, and Phosphorus-Centered Polycyclic π-Conjugated Systems. Chem. Rev. 2019, 119 (14), 8291–8331. 10.1021/acs.chemrev.8b00637. 30860363

[ref2] Parke S. M.; Boone M. P.; Rivard E. Marriage of Heavy Main Group Elements with π-Conjugated Materials for Optoelectronic Applications. Chem. Commun. 2016, 52 (61), 9485–9505. 10.1039/C6CC04023C. 27344980

[ref3] Ding L.; Yu Z.-D.; Wang X.-Y.; Yao Z.-F.; Lu Y.; Yang C.-Y.; Wang J.-Y.; Pei J. Polymer Semiconductors: Synthesis, Processing, and Applications. Chem. Rev. 2023, 123 (12), 7421–7497. 10.1021/acs.chemrev.2c00696. 37232480

[ref4] Zhang L.; Cao Y.; Colella N. S.; Liang Y.; Brédas J.-L.; Houk K. N.; Briseno A. L. Unconventional, Chemically Stable, and Soluble Two-Dimensional Angular Polycyclic Aromatic Hydrocarbons: From Molecular Design to Device Applications. Acc. Chem. Res. 2015, 48 (3), 500–509. 10.1021/ar500278w. 25458442

[ref5] Mamada M.; Hayakawa M.; Ochi J.; Hatakeyama T. Organoboron-Based Multiple-Resonance Emitters: Synthesis, Structure–Property Correlations, and Prospects. Chem. Soc. Rev. 2024, 53 (3), 1624–1692. 10.1039/D3CS00837A. 38168795

[ref6] Wang W.; Shao X. Synthesis and Derivatization of Hetera-Buckybowls. Org. Biomol. Chem. 2021, 19 (1), 101–122. 10.1039/D0OB01931C. 33196065

[ref7] Märkl G. 2,4,6-Triphenylphosphabenzene. Angew. Chem., Int. Ed. Engl. 1966, 5 (9), 846–847. 10.1002/anie.196608463.

[ref8] Savateev A.; Vlasenko Y.; Shtil N.; Kostyuk A. Reduction of λ^5^-Phosphinines. Eur. J. Inorg. Chem. 2016, 2016 (5), 628–632. 10.1002/ejic.201500856.

[ref9] Hashimoto N.; Umano R.; Ochi Y.; Shimahara K.; Nakamura J.; Mori S.; Ohta H.; Watanabe Y.; Hayashi M. Synthesis and Photophysical Properties of λ^5^-Phosphinines as a Tunable Fluorophore. J. Am. Chem. Soc. 2018, 140 (6), 2046–2049. 10.1021/jacs.7b13018. 29400451

[ref10] Habicht M. H.; Wossidlo F.; Bens T.; Pidko E. A.; Müller C. 2-(Trimethylsilyl)-λ^3^-Phosphinine: Synthesis, Coordination Chemistry, and Reactivity. Chem.—Eur. J. 2018, 24 (4), 944–952. 10.1002/chem.201704539. 29068102

[ref11] Ashe A. J. I. Phosphabenzene and Arsabenzene. J. Am. Chem. Soc. 1971, 93 (13), 3293–3295. 10.1021/ja00742a038.

[ref12] Ashe A. J. I.; Chan W.-T. Preparation of 2-Substituted Arsabenzenes. J. Org. Chem. 1979, 44 (9), 1409–1413. 10.1021/jo01323a010.

[ref13] Boese R.; Finke N.; Henkelmann J.; Maier G.; Paetzold P.; Reisenauer H. P.; Schmid G. Synthese und Strukturuntersuchung von Pyridin-Borabenzol und Pyridin-2-Boranaphthalin. Chem. Ber. 1985, 118 (4), 1644–1654. 10.1002/cber.19851180431.

[ref14] Fu G. C. The Chemistry of Borabenzenes (1986–2000). Adv. Organomet. Chem. 2001, 47, 101–119. 10.1016/S0065-3055(01)47010-9.

[ref15] Wakita K.; Tokitoh N.; Okazaki R.; Takagi N.; Nagase S. Crystal Structure of a Stable Silabenzene and Its Photochemical Valence Isomerization into the Corresponding Silabenzvalene. J. Am. Chem. Soc. 2000, 122 (23), 5648–5649. 10.1021/ja000309i.

[ref16] Wakita K.; Tokitoh N.; Okazaki R.; Nagase S. Synthesis and Properties of an Overcrowded Silabenzene Stable at Ambient Temperature. Angew. Chem., Int. Ed. 2000, 39 (3), 634–636. 10.1002/(SICI)1521-3773(20000204)39:3<634::AID-ANIE634>3.0.CO;2-#. 10671282

[ref17] Tokitoh N.; Wakita K.; Matsumoto T.; Sasamori T.; Okazaki R.; Takagi N.; Kimura M.; Nagase S. The Chemistry of Stable Silabenzenes. J. Chin. Chem. Soc. 2008, 55 (3), 487–507. 10.1002/jccs.200800073.

[ref18] Nakata N.; Oikawa T.; Matsumoto T.; Kabe Y.; Sekiguchi A. Silyl-Substituted 1,4-Disila(Dewar Benzene): New Synthesis and Unexpected Insertion of CO into the Si–Si Bond To Form a Disilyl Ketone. Organometallics 2005, 24 (14), 3368–3370. 10.1021/om050261n.

[ref19] Schlesinger H. I.; Ritter D. M.; Burg A. B. Hydrides of Boron. X. The Preparation and Preliminary Study of the New Compound B_2_H_7_N. J. Am. Chem. Soc. 1938, 60 (10), 2297–2300. 10.1021/ja01277a006.

[ref20] Hohnstedt L. F.; Haworth D. T. Preparation of N-Trisubstituted Borazines by Reduction of B-Trichloroborazines. J. Am. Chem. Soc. 1960, 82 (1), 89–92. 10.1021/ja01486a019.

[ref21] Braunschweig H.; Damme A.; Jimenez-Halla J. O. C.; Pfaffinger B.; Radacki K.; Wolf J. Metal-Mediated Synthesis of 1,4-Di-*tert*-butyl-1,4-azaborine. Angew. Chem., Int. Ed. 2012, 51 (40), 10034–10037. 10.1002/anie.201205795. 22952191

[ref22] Chrostowska A.; Xu S.; Lamm A. N.; Mazière A.; Weber C. D.; Dargelos A.; Baylère P.; Graciaa A.; Liu S.-Y. UV-Photoelectron Spectroscopy of 1,2- and 1,3-Azaborines: A Combined Experimental and Computational Electronic Structure Analysis. J. Am. Chem. Soc. 2012, 134 (24), 10279–10285. 10.1021/ja303595z. 22616808 PMC3380147

[ref23] Agou T.; Kobayashi J.; Kawashima T. Dibenzophosphaborin: A Hetero-π-Conjugated Molecule with Fluorescent Properties Based on Intramolecular Charge Transfer between Phosphorus and Boron Atoms. Org. Lett. 2005, 7 (20), 4373–4376. 10.1021/ol051537q. 16178536

[ref24] Barnard J. H.; Brown P. A.; Shuford K. L.; Martin C. D. 1,2-Phosphaborines: Hybrid Inorganic/Organic P–B Analogues of Benzene. Angew. Chem., Int. Ed. 2015, 54 (41), 12083–12086. 10.1002/anie.201507003. 26315985

[ref25] Yang W.; Krantz K. E.; Dickie D. A.; Molino A.; Wilson D. J. D.; Gilliard R. J. Jr. Crystalline BP-Doped Phenanthryne via Photolysis of The Elusive Boraphosphaketene. Angew. Chem., Int. Ed. 2020, 59 (10), 3971–3975. 10.1002/anie.201916362. 31912624

[ref26] Hatakeyama T.; Hashimoto S.; Seki S.; Nakamura M. Synthesis of BN-Fused Polycyclic Aromatics via Tandem Intramolecular Electrophilic Arene Borylation. J. Am. Chem. Soc. 2011, 133 (46), 18614–18617. 10.1021/ja208950c. 22026463

[ref27] Hertz V. M.; Bolte M.; Lerner H.-W.; Wagner M. Boron-Containing Polycyclic Aromatic Hydrocarbons: Facile Synthesis of Stable, Redox-Active Luminophores. Angew. Chem. 2015, 127 (30), 8924–8928. 10.1002/ange.201502977. 26060003

[ref28] Farrell J. M.; Mützel C.; Bialas D.; Rudolf M.; Menekse K.; Krause A.-M.; Stolte M.; Würthner F. Tunable Low-LUMO Boron-Doped Polycyclic Aromatic Hydrocarbons by General One-Pot C–H Borylations. J. Am. Chem. Soc. 2019, 141 (22), 9096–9104. 10.1021/jacs.9b04675. 31117551

[ref29] Sun W.; Guo J.; Fan Z.; Yuan L.; Ye K.; Dou C.; Wang Y. Ribbon-Type Boron-Doped Polycyclic Aromatic Hydrocarbons: Conformations, Dynamic Complexation and Electronic Properties. Angew. Chem., Int. Ed. 2022, 61 (40), e202209271 10.1002/anie.202209271. 35950548

[ref30] Yuan K.; Gupta A. K.; Si C.; Uzelac M.; Zysman-Colman E.; Ingleson M. J. Brominated B1-Polycyclic Aromatic Hydrocarbons for the Synthesis of Deep-Red to Near-Infrared Delayed Fluorescence Emitters. Org. Lett. 2023, 25 (31), 5880–5884. 10.1021/acs.orglett.3c02167. 37498083 PMC10425980

[ref31] Kawai S.; Nakatsuka S.; Hatakeyama T.; Pawlak R.; Meier T.; Tracey J.; Meyer E.; Foster A. S. Multiple Heteroatom Substitution to Graphene Nanoribbon. Sci. Adv. 2018, 4 (4), eaar7181 10.1126/sciadv.aar7181. 29662955 PMC5898832

[ref32] Sano Y.; Shintani T.; Hayakawa M.; Oda S.; Kondo M.; Matsushita T.; Hatakeyama T. One-Shot Construction of BN-Embedded Heptadecacene Framework Exhibiting Ultra-Narrowband Green Thermally Activated Delayed Fluorescence. J. Am. Chem. Soc. 2023, 145 (21), 11504–11511. 10.1021/jacs.3c02873. 37192399

[ref33] Ando N.; Yamada T.; Narita H.; Oehlmann N. N.; Wagner M.; Yamaguchi S. Boron-Doped Polycyclic π-Electron Systems with an Antiaromatic Borole Substructure That Forms Photoresponsive B–P Lewis Adducts. J. Am. Chem. Soc. 2021, 143 (26), 9944–9951. 10.1021/jacs.1c04251. 34109785

[ref34] The term “cross-conjugated” is used here according to common convention, in which it is not simply a purely electronic descriptor but rather also one that describes connectivity in systems of alternating single and double bonds.^35^

[ref35] Phelan N. F.; Orchin M. Cross Conjugation. J. Chem. Educ. 1968, 45 (10), 633 10.1021/ed045p633.

[ref36] Hopf H. Classics in Hydrocarbon Chemistry: Syntheses, Concepts, Perspectives; John Wiley & Sons, 2000.

[ref37] Loy D. A.; Jamison G. M.; McClain M. D.; Alam T. M. Spontaneous Polymerization of Phenylphosphaethyne. J. Polym. Sci., Part A: Polym. Chem. 1999, 37 (2), 129–133. 10.1002/(SICI)1099-0518(19990115)37:2<129::AID-POLA3>3.0.CO;2-Y.

[ref38] Wright V. A.; Gates D. P. Poly(p-Phenylenephosphaalkene): A π-Conjugated Macromolecule Containing P=C Bonds in the Main Chain. Angew. Chem., Int. Ed. 2002, 41 (13), 2389–2392. 10.1002/1521-3773(20020703)41:13<2389::AID-ANIE2389>3.0.CO;2-6. 12203603

[ref39] Wright V. A.; Patrick B. O.; Schneider C.; Gates D. P. Phosphorus Copies of PPV: π-Conjugated Polymers and Molecules Composed of Alternating Phenylene and Phosphaalkene Moieties. J. Am. Chem. Soc. 2006, 128 (27), 8836–8844. 10.1021/ja060816l. 16819877

[ref40] Ayhan O.; Eckert T.; Plamper F. A.; Helten H. Poly(Iminoborane)s: An Elusive Class of Main-Group Polymers?. Angew. Chem., Int. Ed. 2016, 55 (42), 13321–13325. 10.1002/anie.201607131. 27651296

[ref41] Cross M. J.; Brodie C. N.; Crivoi D. G.; Goodall J. C.; Ryan D. E.; Martínez-Martínez A. J.; Johnson A.; Weller A. S. Dehydropolymerization of Amine–Boranes Using Bis(Imino)Pyridine Rhodium Pre-Catalysis: σ-Amine–Borane Complexes, Nanoparticles, and Low Residual-Metal BN–Polymers That Can Be Chemically Repurposed. Chem.—Eur. J. 2023, 29 (60), e202302110 10.1002/chem.202302110. 37530441 PMC10947130

[ref42] Bradford T. A.; Payne A. D.; Willis A. C.; Paddon-Row M. N.; Sherburn M. S. Practical Synthesis and Reactivity of [3]Dendralene. J. Org. Chem. 2010, 75 (2), 491–494. 10.1021/jo9024557. 20000615

[ref43] Bojase G.; Payne A. D.; Willis A. C.; Sherburn M. S. One-Step Synthesis and Exploratory Chemistry of [5]Dendralene. Angew. Chem. 2008, 120 (5), 924–926. 10.1002/ange.200704470. 18092312

[ref44] Bradford T. A.; Payne A. D.; Willis A. C.; Paddon-Row M. N.; Sherburn M. S. Practical Synthesis and Reactivity of [3]Dendralene. J. Org. Chem. 2010, 75 (2), 491–494. 10.1021/jo9024557. 20000615

[ref45] Miller N. A.; Willis A. C.; Paddon-Row M. N.; Sherburn M. S. Chiral Dendralenes for Rapid Access to Enantiomerically Pure Polycycles. Angew. Chem., Int. Ed. 2007, 46 (6), 937–940. 10.1002/anie.200603335. 17171747

[ref46] Miller N. A.; Willis A. C.; Sherburn M. S. Formal Total Synthesis of Triptolide. Chem. Commun. 2008, (10), 1226–1228. 10.1039/b718754h. 18309425

[ref47] Newton C. G.; Drew S. L.; Lawrence A. L.; Willis A. C.; Paddon-Row M. N.; Sherburn M. S. Pseudopterosin Synthesis from a Chiral Cross-Conjugated Hydrocarbon through a Series of Cycloadditions. Nat. Chem. 2015, 7 (1), 82–86. 10.1038/nchem.2112. 25515894

[ref48] Pronin S. V.; Shenvi R. A. Synthesis of a Potent Antimalarial Amphilectene. J. Am. Chem. Soc. 2012, 134 (48), 19604–19606. 10.1021/ja310129b. 23153381

[ref49] Lu H.-H.; Pronin S. V.; Antonova-Koch Y.; Meister S.; Winzeler E. A.; Shenvi R. A. Synthesis of (+)-7,20-Diisocyanoadociane and Liver-Stage Antiplasmodial Activity of the Isocyanoterpene Class. J. Am. Chem. Soc. 2016, 138 (23), 7268–7271. 10.1021/jacs.6b03899. 27244042 PMC5851774

[ref50] Price A. N.; Nichol G. S.; Cowley M. J. Phosphaborenes: Accessible Reagents for the Synthesis of C–C/P–B Isosteres. Angew. Chem., Int. Ed. 2017, 56 (33), 9953–9957. 10.1002/anie.201705050. 28643472

[ref51] Linti G.; Nöth H.; Polborn K.; Paine R. T. An Allene-Analogous Boranylidenephosphane with B P Double Bond: 1,1-Diethylpropyl(2,2,6,6-Tetramethylpiperidino)-Boranylidenephosphane-P-Pentacarbonylchromium. Angew. Chem., Int. Ed. Engl. 1990, 29 (6), 682–684. 10.1002/anie.199006821.

[ref52] Kuhn N.; Kratz T. Synthesis of Imidazol-2-Ylidenes by Reduction of Imidazole-2(3*H*)-Thiones. Synthesis 1993, 1993 (6), 561–562. 10.1055/s-1993-25902.

[ref53] Appel R.; Knoll F.; Ruppert I. Phospha-Alkenes and Phospha-Alkynes, Genesis and Properties of the (p-p)π-Multiple Bond. Angew. Chem., Int. Ed. Engl. 1981, 20 (9), 731–744. 10.1002/anie.198107311.

[ref54] Jie X.; Chen C.; Daniliuc C. G.; Kehr G.; Erker G. Boraalkenes Made by a Hydroboration Route: Cycloaddition and B=C Bond Cleavage Reactions. Angew. Chem., Int. Ed. 2023, 62 (4), e202214700 10.1002/anie.202214700. 36433899

[ref55] Boros S.; Gáspári Z.; Batta G. Accurate NMR Determinations of Proton–Proton Distances. Annu. Rep. NMR Spectrosc. 2018, 94, 1–39. 10.1016/bs.arnmr.2017.12.002.

[ref56] Hopf H.; Sherburn M. S. Dendralenes Branch Out: Cross-Conjugated Oligoenes Allow the Rapid Generation of Molecular Complexity. Angew. Chem., Int. Ed. 2012, 51 (10), 2298–2338. 10.1002/anie.201102987. 22337341

[ref57] Ohtsuki K.; Walsgrove H. T. G.; Hayashi Y.; Kawauchi S.; Patrick B. O.; Gates D. P.; Ito S. Diels–Alder Reactions of 1-Phosphabutadienes: A Highly Selective Route to P=C-Substituted Phosphacyclohexenes. Chem. Commun. 2020, 56 (5), 774–777. 10.1039/C9CC08997G. 31845681

[ref58] Doxsee K. M.; Shen G. S.; Knobler C. B. Reactivity of 1,2-Dihydrophosphetes: Formation and Structural Characterization of a Formal [4 + 2] Cycloadduct. J. Chem. Soc., Chem. Commun. 1990, (22), 1649–1650. 10.1039/c39900001649.

[ref59] Huy N. H. T.; Mathey F. 1,2-Dihydrophosphetes as Masked 1-Phosphabutadienes. Tetrahedron Lett. 1988, 29 (25), 3077–3078. 10.1016/0040-4039(88)85089-5.

