# Supplementary material for: Heavy Heterodendralenes: Structure and Reactivity of Phosphabora[3]dendralenes
Source: J Am Chem Soc. 2024 Aug 14;146(34):23680–5. doi: 10.1021/jacs.4c07850 (PMC11363017; doi:10.1021/jacs.4c07850)
Supplement: Supplementary file 1 — ja4c07850_si_001.pdf [file ja4c07850_si_001.pdf]

Heavy Hetero-Dendralenes: Structure and Reactivity of Phosphabora-[*3*]Dendralenes

Vesela G. Zarkina, Gary S. Nichol, and Michael J. Cowley*

Contents

1. Experimental Details 4
   1.1 General Considerations 4
   1.2 Preparation of 1 5
   1.3 Preparation of 2 6
   1.4 Preparation of 3 7
   1.5 Preparation of 4 8
   1.6 Generation of DMAP-supported Phosphabora-[3]dendralene 10
2. UV-vis Studies 11
   2.1 Spectra of 2 in *n*-pentane 11
   2.2 Spectra of 3 in *n*-pentane 13
3. Density Functional Theory Calculations 14
   3.1 Computational Methods 14
   3.2 Images of Frontier Molecular Orbitals 15
   3.3 Phorphabora-[3]dendralene Conformations M062X/def2SVP Energies 16
   3.4 Time-Dependant Density Functional Theory 16
   3.5 Relative energies of parent [*3*]Dendralene conformations 21
   3.6 Conformational study of a model Phosphabora-[3]dendralene 22
   3.7 Barrier to racemisation of 4 24
4. 2D NOESY, 1D Selective NOESY NMR and Interproton Distances Calculations 25
   4.1 Compound 2 25
   4.2 Compound 3 29
   4.3 Interproton Distance Estimations for 2 and 3 33
5. Variable Temperature NMR studies 35
   5.1 Compound 2 35
   5.2 Compound 3 38
   5.3 Compound 4 42
6. X-Ray Crystallographic Information 44
   6.1 Experimental and Crystal Data for 1 44
   6.2 Experimental and Crystal Data for 2 47
   6.3 Experimental and Crystal Data for 3 50
   6.4 Experimental and Crystal Data for 4 53
7. NMR Spectra 56
   7.1 Compound 1 56

7.2 Compound 2 60
7.3 Compound 3 64
7.4 Compound 4 68
7.5 DMAP-supported Phosphabora[3]-dendralene 72
8. References 76

1. Experimental Details

1.1 General Considerations

All synthetic and analytical procedures were carried out under a dry, oxygen-free argon atmosphere using standard Schlenk or glovebox techniques. All glass apparatus used was stored in a drying oven at 110 °C prior to use. All solvents, with the exception of deuterated ones and benzene, were obtained from a commercial solvent purification system (from Inert Corporation). Benzene was distilled from sodium and benzophenone under argon. Deuterated solvents were distilled from potassium under argon. All solvents, including deuterated ones, were stored over 4 Å molecular sieves.

2,4,6-tri-*tert*-butylphenylphosphine (Mes*PH_2),[1] 1-(dichloroboryl)-2,2,6,6-*tetra*methylpiperidine (tmpBCl_2),[2] diphosphadiboretane $(Mes*PBtmp)_2$,[3] 1,3-dihydro-1,3,4,5-*tetra*methyl-2H-imidazol-2-ylidene and 1,3-dihydro-4,5-dimethyl-1,3-*bis*(1-methylethyl)-2H-imidazol-2-ylidene were synthesised according to literature procedures.[4] Phenylacetylene, 1-ethynylhexene and dimethyl acetylene dicarboxylate were passed through a plug of alumina and freeze-pump-thawed immediately prior to use. All other reagents were obtained commercially and used as supplied without any further purification.

NMR spectra were recorded with Bruker AVA400/BBFO+ probe, AVA500/DCH cryo-probe, AVA600/TCI cryo-probe or PRO500/Prodigy cryo-probe spectrometers. 2D experiments were utilised for some assignments. ^{1}H and ^{13}C NMR were referenced to residual solvent peaks, ^{31}P and ^{11}B NMR were referenced externally to phosphoric acid and trimethyl borate. Readers may notice background signals from ~30 to –40 ppm in our ^{11}B NMR spectra. These arise from glass components of the cryoprobes used in our spectrometers and the background signals thus cannot be mitigated using quartz NMR tubes. UV/Vis spectra were acquired under argon in *n*-pentane, unless specified otherwise, using a Varian Cary 50 scan UV-visible spectrophotometer in 0.1 cm quartz cuvettes. Melting temperatures were recorded using a Stuart SMP10 digital melting point apparatus in a sealed capillary tube under an argon atmosphere. All elemental analysis was carried out by Elemental Microanalysis Ltd.

1.2 Preparation of 1

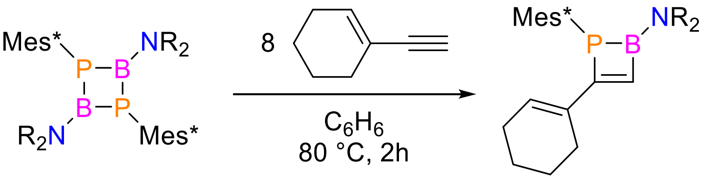

I **1**

I (2.00 g, 2.34 mmol) and 1-ethynylcyclohexene (2.20 mL, 18.71 mmol) were dissolved in benzene (40 mL) to give a clear yellow solution. The mixture was heated at 80 °C for two hours, resulting in a clear bright orange solution. All volatiles were removed *in vacuo*, yielding a bright orange foam. *n*-Hexane (20 mL) was added until the foam was dissolved, and the total volume of the clear bright orange mixture was reduced to approx.10 mL. Analytically pure material in the form of flat yellow crystals were obtained from storage of the solution at room temperature for 7 days (1.65 g, 3.09 mmol, 66%).

^{1}H NMR (500.2 MHz, 300 K, C_6D_6): δ 7.53 (s, 2H, Mes* 3,5-C**H**), 6.24 (d, 1H, $^2J_{H\text{-}P}$ = 25.70 Hz, PBC(**H**)C(Cy)), 5.95 (m, 1H, Cy C=C**H**), 2.21 (m, 2H, Cy 2-C**H**$_2$), 2.01(m, 2H, Cy 5-C**H**$_2$), 1.79 (s, br, 18H, Mes* 2,6-^{t}Bu C**H**$_3$), 1.53 (m, 4H, tmp 3,5-C**H**$_2$), 1.42 (m, 6H, tmp 4-C**H**$_2$ and Cy 3,4-C**H**$_2$), 1.38 (s, 6H, tmp 2,6-C**H**$_3$), 1.34 (s, 9H, Mes* 4-^{t}Bu C**H**$_3$), 1.30 (br, 6H, tmp 2,6-C**H**$_3$).

^{13}C{^{1}H} NMR (125.8 MHz, 300 K, C_6D_6): δ 180.31 (d, $^1J_{C\text{-}P}$ = 20.1 Hz, PBC(H)**C**(Cy)), 159.49 (s, br, Mes* Ar 2,6-**C**), 149.70 (d, $^4J_{C\text{-}P}$ = 2.3 Hz, Mes* Ar 4-**C**), 137.05 (d, $^2J_{C\text{-}P}$ = 11.8 Hz, Cy 1-**C**), 136.31 (d, $^1J_{C\text{-}P}$ = 80.9 Hz, Mes* Ar 1-**C**), 134.73 (d, $^3J_{C\text{-}P}$ = 2.4 Hz, Cy 6-**C**H), 132.61 (d, $^2J_{C\text{-}P}$ = 37.5 Hz, PB**C**(H)C(Cy)), 122.49 (d, $^3J_{C\text{-}P}$ = 5.5 Hz, Mes* Ar 3,5- **C**H), 56.31 (s, tmp 2,6-**C**Me$_2$), 39.67(s, tmp 3,5-**C**H$_2$ and Cy 3,4-**C**H$_2$) , 34.87 (s, Mes* 4-**C**Me$_3$), 34.56 (s, br, Mes* 2,6-**C**Me$_2$), 32.18 (br, Mes* 2,6-^{t}Bu **C**H$_3$), 31.53 (s, Mes* 4-^{t}Bu **C**H$_3$), 26.64 (s, Cy 5-**C**H$_2$), 25.63 (s, Cy 2-**C**H$_2$), 23.00 (s, tmp 3,5-**C**H$_2$), 22.43 (s, tmp 2,2,6,6-**C**H$_3$), 16.28 (s, tmp 4-**C**H$_2$).

^{11}B{^{1}H} NMR (160.5 MHz, 300 K, C_6D_6): δ 43.68.

^{31}P{^{1}H} NMR (202.5 MHz, 300 K, C_6D_6): δ -45.19.

High resolution mass spectrometry: calculated mass of $C_{35}H_{57}BNP$ = 533.43162 Da, M^+ = 533.429836 Da.

Melting point: 153-155 °C.

UV-vis (λ_{max}): shoulder peaks at 249 and 285 nm.

Elemental Analysis: Found (%): C 78.95, H 10.69, N 2.63. Calculated (%) for $C_{35}H_{57}BNP$: C 78.78, H 10.77, N 2.62.

1.3 Preparation of 2

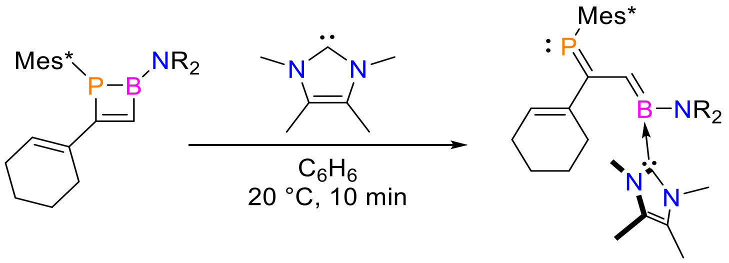

1 **2**

1 (0.40 g, 0.75 mmol) and 1,3-dihydro-1,3,4,5-*tetra*methyl-2H-imidazol-2-ylidene (0.09 g, 0.75 mmol) were dissolved in benzene (15 mL). The immediate formation of a dark red solution and needle-like crystals was observed at room temperature. The mixture was stirred overnight at room temperature and analytically pure material in the form of red needle-like crystals (0.35 g, 0.53 mmol, 72%) was obtained by separation from the mother liquors *via* filtration.

^{1}H NMR (500 MHz, Toluene-d$_8$): δ 7.66 (s, 2H, Mes* 3,5-C**H**), 5.66 (apparent quart, $^1J_{H-H}$ = 4.0, 2.3 Hz, 1H, Cy C=C**H**), 4.85 (d, $^2J_{H-P}$ = 7.22 Hz, 1H, B=C-**H**), 3.20 (s, 6H, ^{Me}IMe N-C**H**$_3$), 2.50 (s, br, 2H, Cy 6-C**H**$_2$), 1.99 (s, 18H, Mes* 2,6-^{t}Bu C**H**$_3$), 1.92-1.71 (br, 2H, Cy 3-C**H**$_2$), 1.63 (s, br, 4H, tmp 3,5-C**H**$_2$), 1.49 (s, 9H, Mes* 4-^{t}Bu C**H**$_3$), 1.45-1.37 (br, 2H, Cy 5-C**H**$_2$), 1.36 (s, 6H, ^{Me}IMe 3,4-C**H**$_3$),1.36-1.30 (br, 2H, tmp 4-C**H**$_2$), 1.30-0.61 (br, 12H, tmp 2,2,6,6-C**H**$_3$).

^{13}C{^{1}H} NMR (126 MHz, Toluene-d$_8$): δ 191.53 (d, $^1J_{C-P}$ = 56.1 Hz, P=**C**), 162.58 (^{Me}IMe 3,4-**C**Me), 155.59 (Mes* Ar 2,6-**C**), 147.30 (Mes* Ar 4-**C**), 145.71 (d, $^2J_{C-P}$ = 31.6 Hz, Cy 1-**C**), 144.86 (d, $^1J_{C-P}$ = 70.1 Hz, Mes* Ar 1-**C**), 121.67 (Mes* 3,5-CH), 117.40 (s, br, B=**C**-H), 117.04 (d, $^3J_{C-P}$ = 21.2 Hz, Cy C=CH), 54.63 (s, tmp 2,2,6,6-**C**Me$_2$), 42.71 (s, br, Cy 6-CH$_2$), 39.58 (Mes* 2,6- ^{t}Bu **C**Me$_3$), 35.48 (s, Mes* 4-^{t}Bu **C**Me$_3$), 34.19 (s, br, s, Mes* 2,6-^{t}Bu CH$_3$), 32.86 (s, ^{Me}IMe N-CH$_3$), 32.38 (s, Mes* 4-^{t}Bu **C**H$_3$), 31.66 (d, $^4J_{C-P}$ = 10.0 Hz, Mes* 2,6-^{t}Bu **C**H$_3$), 26.59 (d, $^4J_{C-P}$ = 2.2 Hz, Cy 3-**C**H$_2$), 23.95 (s, Cy 4,5-**C**H$_2$), 23.51 (s, tmp 2,2,6,6-**C**H$_3$), 18.61 (s, tmp 3,5-**C**H$_2$), 8.15 (s, ^{Me}IMe 3,4-CH$_3$).

^{11}B{^{1}H} NMR (160 MHz, Toluene-d$_8$): δ 25.33.

^{31}P{^{1}H} NMR (202 MHz, Toluene-d$_8$): δ 127.50.

High resolution mass spectrometry: calculated mass of $C_{43}H_{71}BN_2P$ = 657.54424 Da, M^+ = 657.54538 Da.

Melting point: 282-283 °C.

UV-vis (λ_{max}, ε): 500 nm; 1,035 dm^{3}mol^{-1}cm^{-1}.

Elemental Analysis: Found (%): C 76.62, H 10.49, N 6.29. Calculated (%) for $C_{43}H_{71}BN_2P$: C 76.69, H 10.57, N 6.39.

1.4 Preparation of 3

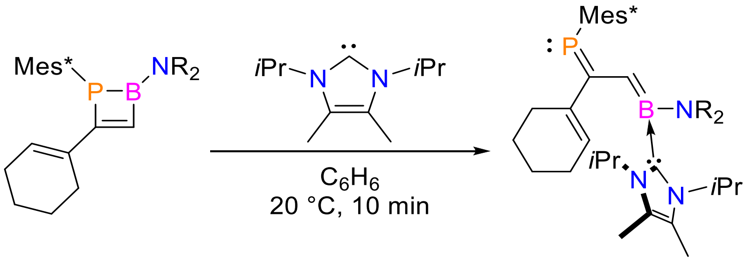

1 **3**

1 (0.30 g, 0.56 mmol) and 1,3-dihydro-4,5-dimethyl-1,3-*bis*(1-methylethyl)- 2H-imidazol-2-ylidene (0.10 g, 0.56 mmol) were dissolved in benzene (15 mL). The immediate formation of a dark purple solution was observed at room temperature. The mixture was stirred overnight at room temperature and all volatiles were removed *in vacuo* the following morning to yield a crude purple solid. *n*-Hexane (10 mL) was added resulting in a deep purple solution which was concentrated to approx. 5 mL. Analytically pure material in the form of square deep purple crystals (0.10 g, 0.14 mmol, 25%) was obtained by separation from the mother liquors *via* filtration.

^{1}H NMR (400 MHz, toluene-d$_8$): δ 7.64 (s, 2H, Mes* 3,5-C**H**), 5.97 (apparent quart, $^{3}J_{H\text{-}H}$ = 4.1 Hz, 1H, Cy C=C**H**), 5.86 (hept, $^{2}J_{H\text{-}H}$ = 7.0 Hz, 2H, ^{iPr}IMe N-C(**H**)Me$_2$), 5.10 (d, $^{3}J_{H\text{-}P}$ = 8.7 Hz, 1H, B=C-**H**), 2.70 (s, 2H, Cy 6-C**H**$_2$), 1.95 (d, $^{4}J_{H\text{-}P}$ = 1.4 Hz, 18H, Mes* 2,6-^{t}Bu C**H**$_3$), 1.72 (br, 2H, Cy 3-C**H**$_2$), 1.68-1.64 (br, tmp 4-CH$_2$), 1.63 (s, 6H, ^{iPr}IMe 3,4-C**H**$_3$), 1.49 (s, 9H, Mes* 4-^{t}Bu C**H**$_3$), 1.43-1.39 (br, 2H, Cy 5-C**H**$_2$), 1.39 (s, br, 2H, Cy 4-C**H**$_2$), 1.37 (s, br, 2H, tmp 3-C**H**$_2$),1.36 (s, 6H, tmp 2,6-C**H**$_3$), 1.31(m, br, 2H, tmp 5-C**H**$_2$), 1.29 (d, $^{2}J_{H\text{-}H}$ = 7.2 Hz, 6H, ^{iPr}IMe 2,5-^{i}Pr C**H**$_3$), 1.12 (d, $^{2}J_{H\text{-}H}$ = 6.9 Hz, 6H, ^{iPr}IMe 2,5-^{i}Pr C**H**$_3$), 0.82 (s, 6H, tmp 2,6-C**H**$_3$).

^{13}C{^{1}H} NMR (151 MHz, 300 K, C$_6$D$_6$): δ 189.76 (d, $^{1}J_{C\text{-}P}$ = 55.6 Hz, P=**C**), 155.35 (Mes* Ar 2,6-**C**), 147.13 (Mes* Ar 4-**C**), 143.92 (d, $^{1}J_{C\text{-}P}$ = 66.9 Hz, Mes* Ar 1-**C**), 143.05 (d, $^{2}J_{C\text{-}P}$ = 30.4 Hz, Cy 1-**C**),125.30 (s, ^{iPr}IMe 3,4-**C**Me), 121.70 (Mes* 3,5-**C**H), 120.46 (d, $^{3}J_{C\text{-}P}$ = 22.6 Hz, Cy C=**C**H), 118.45 (s, br, B=**C**-H), 55.17 (s, tmp 2,6-**C**Me$_2$), 53.90 (s, tmp 2,6-**C**Me$_2$), 49.34 (s, ^{iPr}IMe 2,5-**C**H(Me)$_2$), 44.98 (s, tmp 4-**C**H$_2$), 41.84 (s, tmp 3,5-**C**H$_2$), 39.18 (s, Mes* 2,6- ^{t}Bu **C**Me$_3$), 35.15 (s, Mes* 4-^{t}Bu **C**Me$_3$), 33.71 (d, $^{4}J_{C\text{-}P}$ = 8.0 Hz, Mes* 2,6-^{t}Bu **C**H$_3$), 32.50 (s, br, tmp 2,2,6,6-**C**H$_3$), 32.04 (s, Mes* 4-^{t}Bu **C**H$_3$), 30.73 (d, $^{3}J_{C\text{-}P}$ = 24.7 Hz, Cy 6-**C**H$_2$), 26.48 (s, Cy 3-**C**H$_2$), 24.25 (d, $^{4}J_{C\text{-}P}$ = 3.5 Hz, Cy 5-**C**H$_2$), 23.49 (s, Cy 4-**C**H$_2$), 21.87 (s, ^{iPr}IMe 2,5-^{i}Pr **C**H$_3$), 21.74 (s, br, ^{iPr}IMe 2,5-^{i}Pr **C**H$_3$), 18.86 (s, ^{iPr}IMe 3,4-**C**H$_3$), 10.03 (^{iPr}IMe 1-**C**).

^{11}B{^{1}H} NMR (160.5 MHz, 300 K, C$_6$D$_6$): δ 24.89 (s, br).

^{31}P{^{1}H} NMR (202.5 MHz, 300 K, C$_6$D$_6$): δ 137.31 (s).

High resolution mass spectrometry: calculated mass of $C_{46}H_{77}BN_3P$ = 713.59427 Da, M^{+} = 713.594527 Da.

Melting point: 113-115 °C

UV-vis (λ_{max}, ε): 496 nm, 1,847 dm^{3}mol^{-1}cm^{-1}.

Elemental Analysis: Found (%): C 77.39, H 10.87, N 5.89. Calculated (%) for $C_{46}H_{77}BN_3P$: C 76.05, H 10.59, N 6.47.

1.5 Preparation of 4

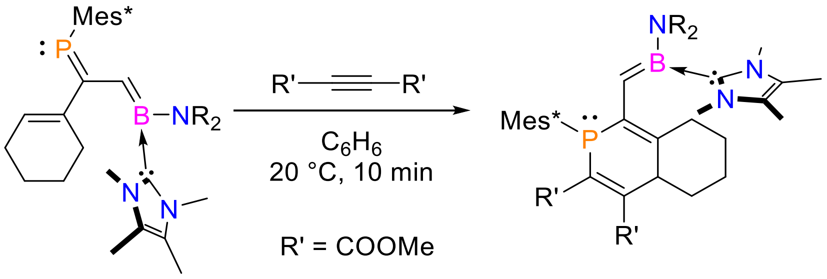

2 **4**

2 (0.15 g, 0.24 mmol) was dissolved in benzene (15 mL). Dimethyl acetylene dicarboxylate (0.03 mL, 0.24 mmol) was added and mixture was stirred at room temperature for 2 h. A colour change from deep pink to deep red was observed. All volatiles were removed *in vacuo* to yield a red oil. *n*-Hexane (20 mL) was added, the suspension was filtered and filtrate was stored at -20 °C for a week, yielding a red solid. Square red analytically pure crystals were obtained *via* recrystallization from hot *n*-hexane (9 mL) and storage at room temperature for 2 days (0.06 g, 3%).

^{1}H NMR (400 MHz, C_6D_6) of major isomer: δ 7.65 (d, $^3J_{H\text{-}P}$ = 2.3 Hz, 1H, Mes* 5-C**H**), 7.62 (dd, $^2J_{H\text{-}H}$ = 4.9 Hz, $^3J_{H\text{-}P}$ = 2.3 Hz, Mes* 3-C**H**), 4.80 (d, $^2J_{H\text{-}P}$ = 7.8 Hz, 1H, B=C-**H**), 3.86 (m, 1H, 4-C**H**), 3.60 (s, 3H, ^{Me}IMe N-C**H$_3$**), 3.43 (s, 3H, 3-COOC**H$_3$**), 3.39 (s, 3H, 2-COOC**H$_3$**), 3.16 (s, 3H, ^{Me}IMe N-C**H$_3$**), 2.14 (s, 9H, Mes* 2-^{t}Bu), 1.84 (s, 9H, Mes* 6-^{t}Bu), 1.80 (s, 3H, ^{Me}IMe 3,4-C**H**$_3$), 1.68 (s, br, 2H, tmp 2-C**H**$_2$), 1.61 (s, 3H, ^{Me}IMe 3,4-C**H**$_3$), 1.54 (m, 2H, tmp 4-C**H**$_2$), 1.47 (s, br, 2H, tmp 5-C**H**$_2$), 1.43 (s, 3H, tmp 2,6-C**H**$_3$), 1.41(s, 3H, tmp 2,6-C**H**$_3$), 1.38 – 1.34 (m, Mes* 4-^{t}Bu), 1.28 (d, $^1J_{H\text{-}H}$ = 4.09 Hz, 2H, 7-C**H**$_2$), 1.27 (m, 2H, 6-C**H**$_2$), 1.23 (m, 3H, tmp 2,6-C**H**$_3$), 0.92 (dt, $^1J_{H\text{-}H}$ = 11.88 Hz, $^2J_{H\text{-}H}$ = 6.12 Hz, 2H, 5-C**H**$_2$), 0.89 (t, $^1J_{H\text{-}H}$ = 7.1 Hz, 2H, 8-C**H**$_2$), 0.44 (s, 3H, 2,6-C**H**$_3$).

^{1}H NMR (400 MHz, C_6D_6) of minor isomer: δ 7.76 (d, $^3J_{H\text{-}P}$ = 2.2 Hz, Mes* 5-C**H**), 7.73 (dd, $^2J_{H\text{-}H}$ = 5.1 Hz, $^3J_{H\text{-}P}$ = 2.2 Hz, Mes* 3-C**H**), 4.80 (d, $^2J_{H\text{-}P}$ = 7.8 Hz, 1H, B=C-**H**), 3.74 (d, $^1J_{H\text{-}H}$ = 9.48 Hz, 4-C**H**), 3.60 (s, 3H, ^{Me}IMe N-C**H$_3$**), 3.45 (s, 3-COOC**H$_3$**), 3.41 (s, 2-COOC**H$_3$**), 3.16 (s, 3H, ^{Me}IMe N-C**H$_3$**), 2.14 (s, 9H, Mes* 2-^{t}Bu), 1.84 (s, 9H, Mes* 6-^{t}Bu), 1.68 (s, br, 2H, tmp 2-CH$_2$), 1.54 (m, 2H, tmp 4-C**H**$_2$), 1.47 (s, br, 2H, tmp 5-C**H**$_2$), 1.43 (s, 3H, tmp 2,6-C**H**$_3$), 1.41(s, 3H, tmp 2,6-C**H**$_3$), 1.38 – 1.34 (m, Mes* 4-^{t}Bu), 1.28 (d, $^1J_{H\text{-}H}$ = 4.09 Hz, 2H, 7-C**H**$_2$), 1.27 (m, 2H, 6-C**H**$_2$), 1.23 (m, 3H, tmp 2,6-C**H**$_3$), 0.92 (dt, $^1J_{H\text{-}H}$ = 11.88 Hz, $^2J_{H\text{-}H}$ = 6.12 Hz, 2H, 5-C**H**$_2$), 0.89 (t, $^1J_{H\text{-}H}$ = 7.1 Hz, 2H, 8-C**H**$_2$), 0.39 (s, 2,6-C**H**$_3$).

^{13}C{^{1}H} NMR (151 MHz, 300 K, C_6D_6) of major isomer: 169.09 (3-**C**OOCH$_3$), 168.14 (d, $^2J_{C\text{-}P}$ = 14.9 Hz, 2-**C**OOCH$_3$), 161.69 (d, $^1J_{C\text{-}P}$ = 33.6 Hz, 2-**C**), 159.60 (d, $^4J_{C\text{-}P}$ = 8.4 Hz, Mes* Ar 4-**C**), 149.91 (d, $^5J_{C\text{-}P}$ = 2.8 Hz, Mes* ^{t}Bu 4-**C**), 143.52 (d, $^1J_{C\text{-}P}$ = 36.9 Hz, 10-**C**), 133.64 (d, $^2J_{C\text{-}P}$ = 24.4 Hz, 9-**C**), 131.14 (d, $^1J_{C\text{-}P}$ = 57.1 Hz, Mes* **C**-P), 127.42 (d, $^2J_{C\text{-}P}$ = 10.6 Hz, 3-**C**), 124.37 (Mes* Ar 5-**C**H), 124.33 (Mes* Ar 2-C), 123.70 (Mes* Ar 6-C), 122.08 (d, $^3J_{C\text{-}P}$ = 13.7 Hz, Mes* 3-**C**H), 106.41 (br, **C**=B) 54.57 (^{Me}IMe 3,4-**C**), 51.37 (s, 3-COO**C**H$_3$), 50.94 (^{Me}IMe N-**C**H$_3$), 47.80 (d, $^3J_{C\text{-}P}$ = 7.0 Hz, 4-**C**H), 43.73 (tmp 4-**C**H$_2$), 41.68 (tmp 5-**C**H$_2$), 40.48 (Mes* ^{t}Bu 6-**C**), 39.85 (d, $^3J_{C\text{-}P}$ = 9.0 Hz, Mes* ^{t}Bu 2-**C**), 36.72 (Mes* ^{t}Bu 6-**C**H$_3$), 35.80 (d, $^4J_{C\text{-}P}$ = 12.5 Hz, Mes* ^{t}Bu 2-**C**H$_3$), 33.97 (tmp 2-**C**H$_2$), 32.77 (d, $^3J_{C\text{-}P}$ = 11.6 Hz, 8-**C**), 31.97 (2-COO**C**H$_3$), 31.51 (Mes* ^{t}Bu 4-**C**H$_3$), 30.30 (d, $^3J_{C\text{-}P}$ = 3.0 Hz, 5-**C**), 29.63 (^{Me}IMe 3,4-CH$_3$), 29.33 (^{Me}IMe 3,4-**C**H$_3$), 28.34 (7-**C**H$_2$), 27.60 (6-**C**H$_2$), 19.32 (^{Me}IMe 1-**C**), 8.21 (tmp 2,6-**C**H$_3$), 7.88 (tmp 2,6-**C**H$_3$).

$^{13}C\{^1H\}$ NMR (151 MHz, 300 K, C_6D_6) of minor isomer: 169.05 (3-**C**OOCH$_3$), 168.94 (2-**C**OOCH$_3$, d, $^2J_{C\text{-}P}$ = 11.4 Hz), 161.63 (d, $^1J_{C\text{-}P}$ = 36.20 Hz, 2-**C**), 158.06 (d, $^4J_{C\text{-}P}$ = 7.8 Hz, Mes* Ar 4-**C**), 150.18 (d, $^5J_{C\text{-}P}$ = 2.3 Hz, Mes* ^{t}Bu 4-**C**), 143.18 (d, $^1J_{C\text{-}P}$ = 51.9 Hz, 10-**C**), 133.52 (d, $^2J_{C\text{-}P}$ = 17.8 Hz, 9-**C**), 129.18 (d, $^1J_{C\text{-}P}$ = 58.4 Hz, Mes* **C**-P), 127.42 (d, $^2J_{C\text{-}P}$ = 10.6 Hz, 3-**C**), 125.69 (Mes* Ar 5-**C**H), 125.23 (d, $^2J_{C\text{-}P}$ = 10.0 Hz, Mes* Ar 2-**C**), 123.62 (Mes* Ar 6-**C**), 122.56 (d, $^3J_{C\text{-}P}$ = 14.2 Hz, Mes* 3-**C**H), 106.41 (br, **C**=B), 54.32 (^{Me}IMe 3,4-**C**), 51.32 (s, 3-COO**C**H$_3$), 50.94 (^{Me}IMe N-**C**H$_3$), 47.80 (d, $^3J_{C\text{-}P}$ = 7.0 Hz, 4-**C**H), 43.97 (tmp 4-**C**H$_2$) , 42.79 (d, $^4J_{C\text{-}P}$ = 2.9 Hz, 2-COO**C**H$_3$), 41.40 (tmp 5-**C**H$_2$), 40.48 (Mes* ^{t}Bu 6-**C**), 39.70 (d, $^3J_{C\text{-}P}$ = 8.3 Hz, Mes* ^{t}Bu 2-**C**), 35.92 (Mes* ^{t}Bu 6-**C**H$_3$), 35.23 (d, J = 13.7 Hz, Mes* ^{t}Bu 2-**C**H$_3$), 34.41 (tmp 2-**C**H$_2$), 33.19 (d, $^3J_{C\text{-}P}$ = 12.5 Hz, 8-**C**), 31.95 (2-COO**C**H$_3$), 31.57 (Mes* ^{t}Bu 4-**C**H$_3$), 31.04 (s, 5-**C**), 29.08 (^{Me}IMe 3,4-**C**H$_3$), 28.70 (^{Me}IMe 3,4-**C**H$_3$), 27.88 (7-**C**H$_2$), 27.29 (6-**C**H$_2$), 19.19 (^{Me}IMe 1-**C**), 8.28 (tmp 2,6-**C**H$_3$), 7.57 (tmp 2,6-**C**H$_3$).

$^{11}B\{^1H\}$ NMR (160.5 MHz, 300 K, C_6D_6): 24.31 (s, br), 1.85 (s).

$^{31}P\{^1H\}$ NMR (202.5 MHz, 300 K, C_6D_6): δ -34.41 (s, major isomer), -36.16 (s, minor isomer).

High resolution mass spectrometry: calculated mass of $C_{48}H_{75}BN_3O_4P$ = 799.55828 Da, M^+ = 799.559310 Da.

Melting point: 133-135 °C.

UV-vis (λ_{max}, ε): shoulder peaks at 401 and 335 nm.

Elemental Analysis: Found (%): C 71.89, H 9.68, N 5.24. Calculated (%) for $C_{48}H_{75}BN_3O_4P$: C 72.67, H 9.68, N 4.93.

1.6 Generation of DMAP-supported Phosphabora-[3]dendralene

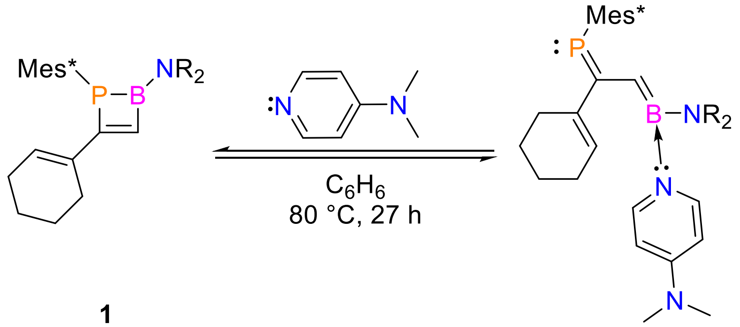

1

1 (0.10 g, 0.19 mmol) and 4-dimethylaminopyridine (0.09 g, 0.75 mmol) were dissolved in benzene (15 mL). The immediate formation of a black solution was observed, and the mixture was heated at 80 °C for 27 hours. The desired product can be isolated as a crude solid by removing the solvent *in vacuo,* yielding a black solid (crude 0.08 g, 0.12 mmol, 87%). Our attempts at crystallisation yielded crystalline DMAP and regenerated **1** in solution.

Crude ^{1}H NMR (500 MHz, Toluene-d$_8$): δ 8.35 (dd, $^1J_{H\text{-}H}$ = 6.4 Hz, $^2J_{H\text{-}H}$ = 3.4 Hz, uncomplexed DMAP 2,6-C**H**), 8.26 (dd, $^1J_{H\text{-}H}$ = 7.5 Hz, $^2J_{H\text{-}H}$ = 2.7 Hz, complexed DMAP 2,6-C**H**), 7.71 (s, 2H, Mes* 3,5-C**H**), 6.07 (dd, $^1J_{H\text{-}H}$ = 6.2 Hz, $^2J_{H\text{-}H}$ = 3.2 Hz, uncomplexed DMAP 3,5-C**H**), 5.59 (d, $^1J_{H\text{-}H}$ = 7.5 Hz, 3H, complexed DMAP 3,5-C**H** and Cy C=C**H** overlapping), 4.26 (d, $^2J_{H\text{-}P}$= 7.2 Hz, B=C-**H**), 2.27 (s, uncomlexed DMAP N-(C**H**$_3$)$_2$), 2.09 (m, 12H, 2,2,6,6-C**H**$_3$), 2.05 (s, br, 18H, Mes* 2,6-^{t}Bu C**H**$_3$), 2.04 (s, complexed DMAP N-(C**H**$_3$)$_2$, 1.53 (s, 9H, Mes* 4- ^{t}Bu CH$_3$). Cyclohexyl 3,4,5,6-CH$_2$ and 2,2,6,6-tetramethyl piperidine 2,3,4-CH$_2$ peaks were not assigned successfully due to the complexity of the spectra. However, the 1.84 – 1.55 (m, 6H) and 1.49 – 1.12 (m, 8H) regions contain the expected number of protons corresponding to those resonances.

^{13}C{^{1}H} NMR (126 MHz, Toluene-d$_8$): δ 192.43 (d, $^1J_{C\text{-}P}$ = 52.13 Hz, P=**C**), 155.83 (Mes* Ar 2,6-**C**), 154.45 (uncomplexed DMAP 4-**C**), 150.80 (complexed DMAP Ar 4-**C**), 147.53 (Mes* Ar 4-**C**), 146.01 (Mes* Ar 3,5-**C**), 145.81 (Cy 1-**C**), 145.05 (d, $^1J_{C\text{-}P}$ = 66.2 Hz, Mes* Ar 1-**C**), 137.85 (uncomplexed DMAP 2,6-**C**), 121.41 (d, $^2J_{C\text{-}P}$ = 22.4 Hz, Cy C=**C**), 107.17 (uncomplexed DMAP 3,5-**C**), 105.16 (complexed DMAP 3,5-**C**), 54.00 (tmp 2,2,6,6-**C**Me$_2$), 49.63 (tmp 3,5-**C**H$_2$), 39.52 (Mes* 2,6-^{t}Bu **C**Me$_3$), 38.85, (Mes* 2,6-^{t}Bu **C**Me$_3$), 38.64 (uncomplexed DMAP N-(**C**H$_3$)$_2$), 35.57 (Mes* 2,6-^{t}Bu **C**H$_3$), 33.75 (br, Mes* 4-^{t}Bu **C**H$_3$), 32.42 (complexed DMAP N-(**C**H$_3$)$_2$). 2,2,6,6-Tetramethyl piperidine 4-C and 2,2,6,6-CH$_3$, as well as cyclohexyl 3,4,5,6-C were not able to be identified due to the complexity of the ^{1}H and ^{1}H-^{13}C HMQC NMR spectra.

^{11}B{^{1}H} NMR (160 MHz, Toluene-d$_8$): δ 30.44.

^{31}P{^{1}H} NMR (202 MHz, Toluene-d$_8$): δ 137.98.

2. UV-vis Studies

2.1 Spectra of 2 in *n*-pentane

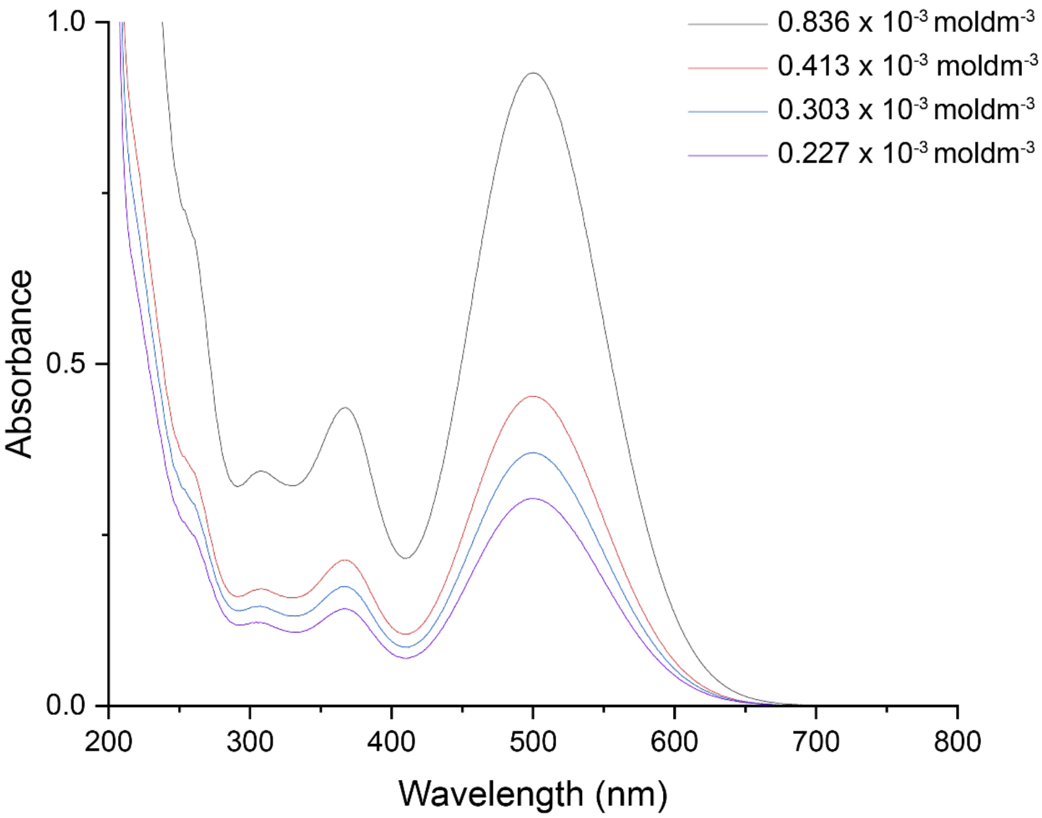

Figure S1. The UV-vis spectrum of **2** in *n*-pentane

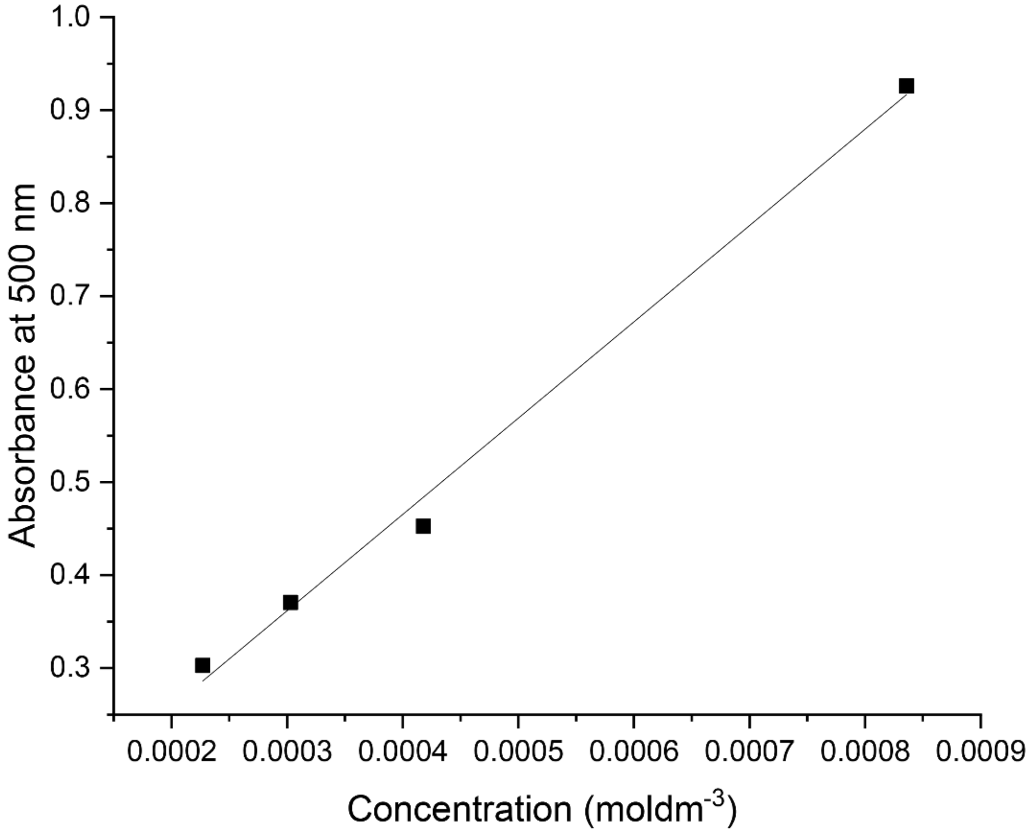

Figure S2. The absorbance at 500 nm against concentration plot for **2**

2.2 Spectra of 3 in *n*-pentane

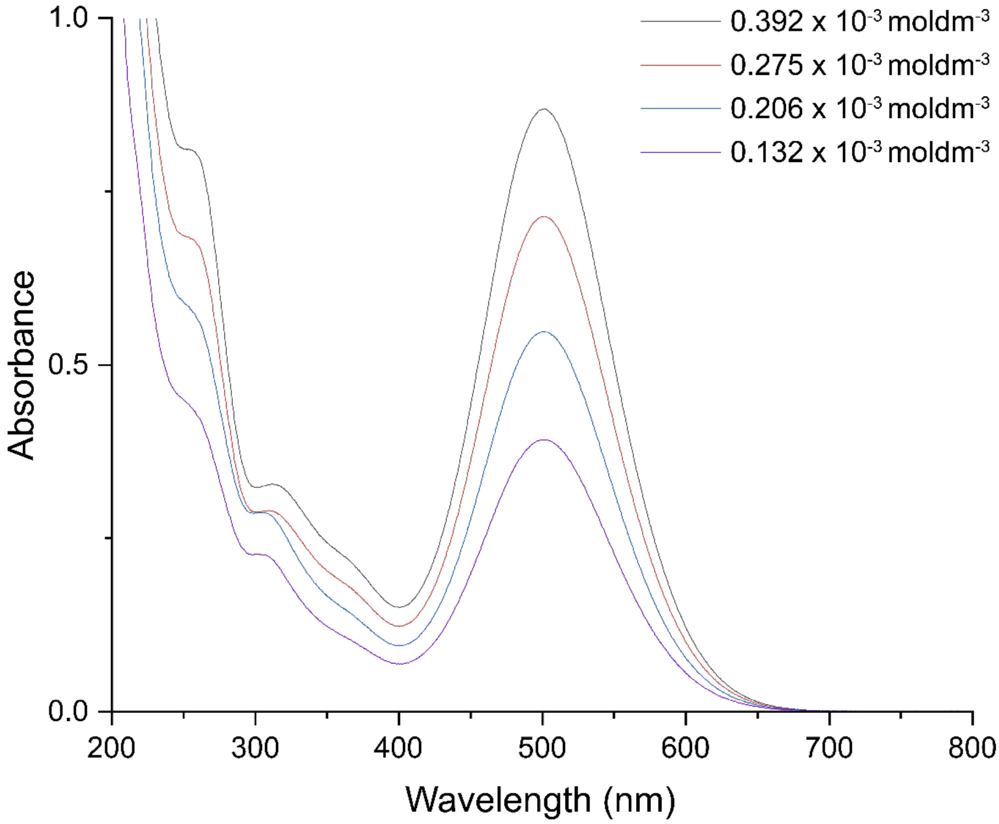

Figure S3. The UV-vis spectrum of **3** in *n*-pentane

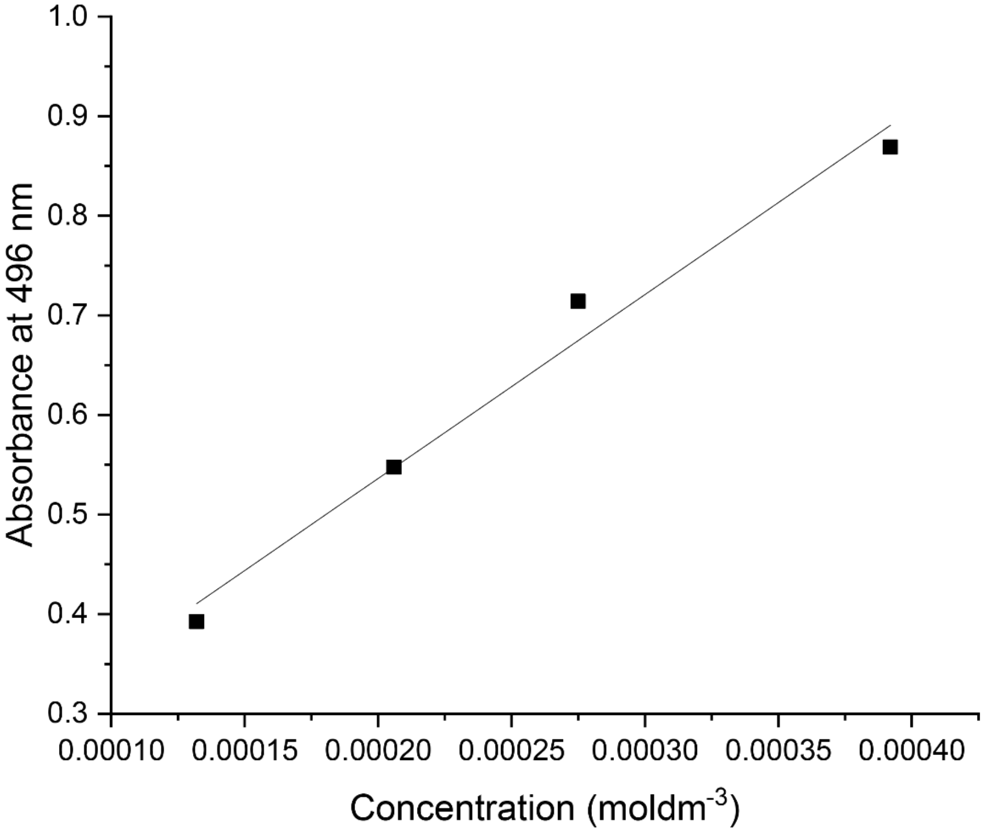

Figure S4. The absorbance at 500 nm against concentration plot for **3**

3. Density Functional Theory Calculations

3.1 Computational Methods

All electronic structure calculations were done using the Gaussian016 (Revision B.01) program. Geometry optimisations were performed at the M062X/def2SVP level of theory. Local minima were confirmed by the absence of imaginary eigenvalues in the hessian matrix, by way of a frequency calculation performed at the same level of theory. Calculations were optimised using a polarisable continuum solvent model (SCRF) using *n*-pentane as a solvent.

The geometry optimisation of **3** was carried out at the M062X/def2SVP level of theory prior to introduction of a polarisable continuum solvent model using *n*-pentane in a single point calculation

3.2 Images of Frontier Molecular Orbitals

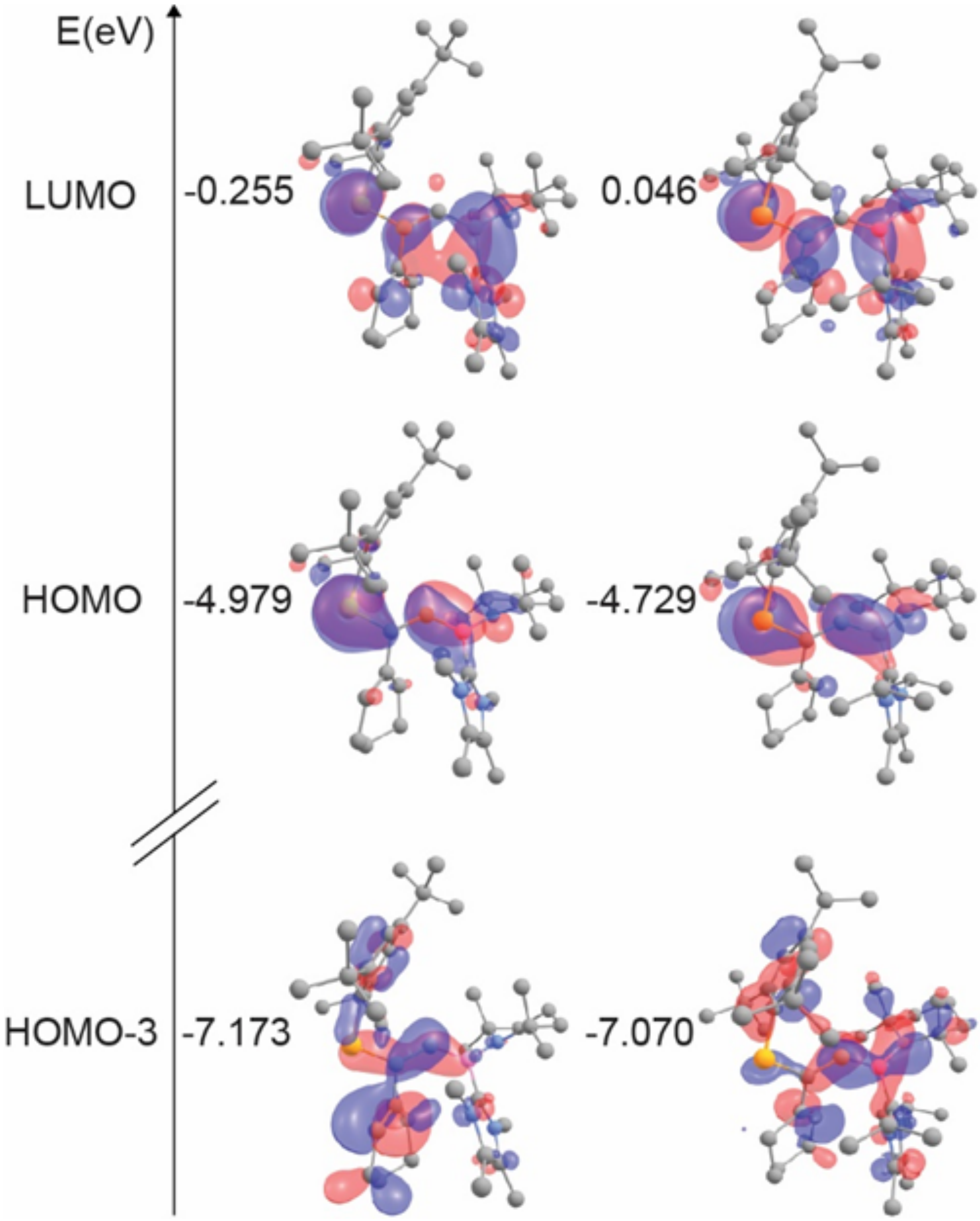

Figure S5. The frontier molecular orbitals and HOMO-3 of **2** (left) and **3** (right) calculated at M062X/Def2SVPP including their energies in eV

3.3 Phorphabora-[3]dendralene Conformations M062X/def2SVP Energies

Table S1. The sums of electronic and thermal energies and their normalised values for **2** at the MO62X/def2SVP level of theory.

Conformation of 2	Sum of Electronic and Thermal Energies (H p^{-1})	Normalised Sum of Electronic and Thermal Energies (kJ mol^{-1})
**gauche** ***cis, trans***	-2169.1262	0.000
**quasi** ***trans, trans***	-2169.1254	2.210

Table S2. The sums of electronic and thermal energies and their normalised values for **3** at the MO62X/def2SVP level of theory.

Conformation of 3	Sum of Electronic and Thermal Energies (H p^{-1})	Normalised Sum of Electronic and Thermal Energies (kJ mol^{-1})
**gauche** ***cis, trans***	-2326.0588	0.000
**quasi** ***trans, trans***	-2326.0620	8.208

3.4 Time-Dependant Density Functional Theory

Time dependent (TD)-DFT calculations were carried out at the M062X/def2SVP level of theory using *n*-pentane as the solvent employing the SCRF solvent model.

Table S3. Summary of the TD-DFT output for phosphabora-[*3*]dendralene **2** in the gauche *cis,trans* conformation at M062X/Def2SVP using *n*-pentane as a solvent.

Wavelength (nm)	Energy (eV)	Transition	Oscillator Strength
**440.43**	2.815	HOMO to LUMO	0.439
**337.81**	3.670	HOMO to LUMO+1	0.005
**310.29**	3.996	HOMO-1 to LUMO<br>HOMO to LUMO+2	0.028
**285.57**	4.342	HOMO to LUMO+3	0.030

**240.71**	5.151	HOMO-1 to LUMO+1	0.127

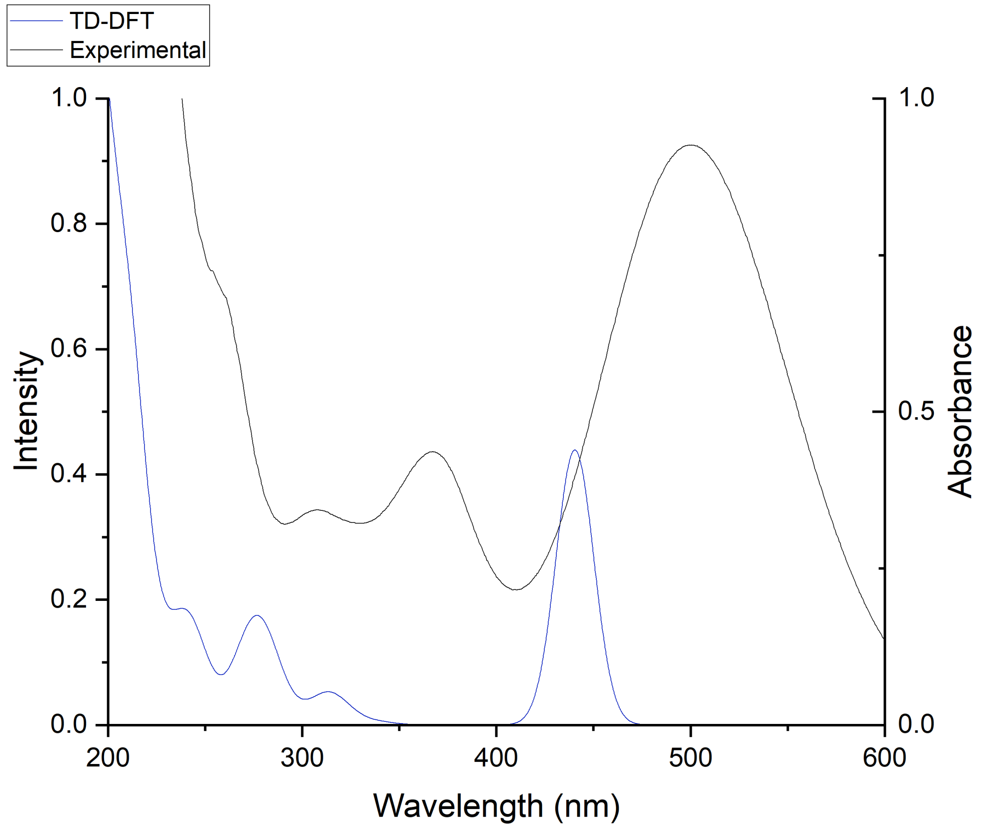

Figure S6. The Predicted (TD-DFT) uv-vis spectrum for **2** (blue) in the gauche *cis,trans* conformation at M062X/Def2SVP including Gaussian broadening with band width on half height equal to 23 nm and the experimental uv-vis spectrum of **2** in *n*-pentane at 0.836 × 10^{-3} mol dm^{-3}

Table S4. Summary of the TD-DFT output for phosphabora-[3]dendralene **2** in the quasi *trans,trans* conformation at M062X/Def2SVP.

Wavelength (nm)	Energy (eV)	Transition	Oscillator Strength
**438.48**	2.828	HOMO to LUMO	0.427
**352.85**	3.154	HOMO to LUMO+1	0.018
**312.80**	3.964	HOMO-3 to LUMO<br>HOMO-1 to LUMO<br>HOMO-1 to LUMO+1	0.026
**275.50**	4.500	HOMO-2 to LUMO	0.105
**242.00**	5.123	HOMO-1 to LUMO+2	0.171

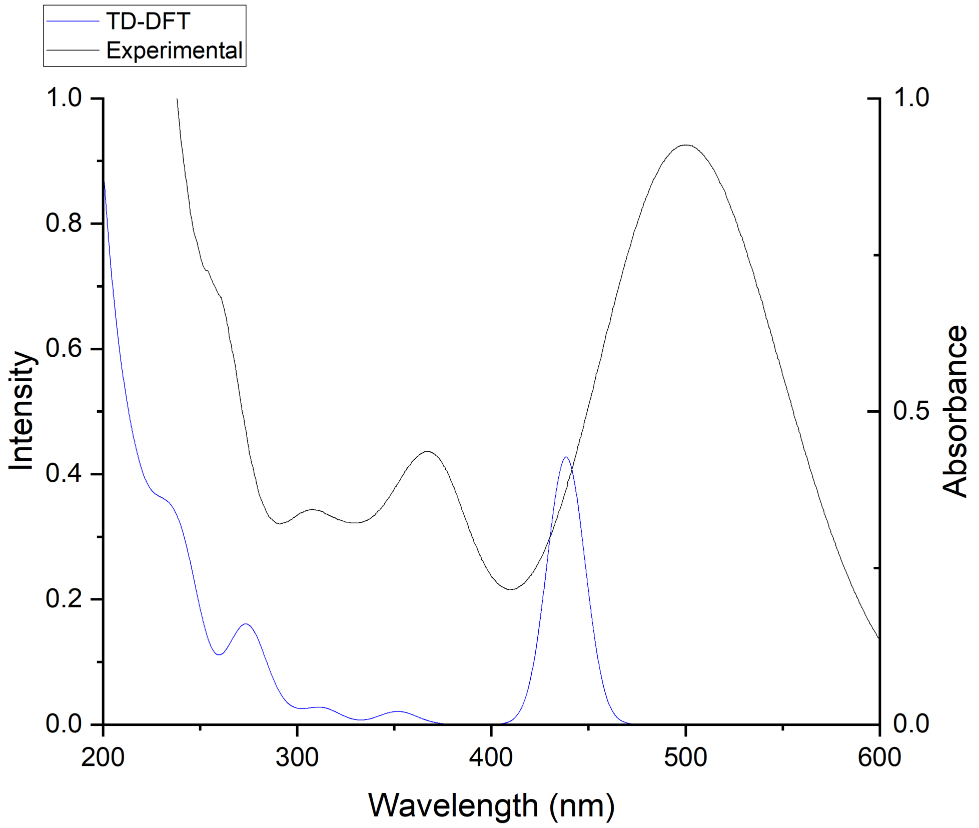

Figure S7. The predicted (TD-DFT) uv-vis spectrum for **2** (blue) in the quasi *trans,trans* conformation at M062X/Def2SVP including Gaussian broadening with band width on half height equal to 23 nm and the experimental uv-vis spectrum of **2** in *n*-pentane at 0.836 × 10^{-3} mol dm^{-3}

Table S5. Summary of the TD-DFT output for phosphabora-[*3*]dendralene **3** in the gauche *cis,trans* conformation at M062X/Def2SVP.

Wavelength (nm)	Energy (eV)	Transition	Oscillator Strength
**458.59**	2.704	HOMO to LUMO	0.419
**328.01**	3.780	HOMO-1 to LUMO<br>HOMO to LUMO+2	0.027
**283.70**	4.370	HOMO-2 to LUMO	0.146
**241.27**	5.139	HOMO-1 to LUMO+1	0.147

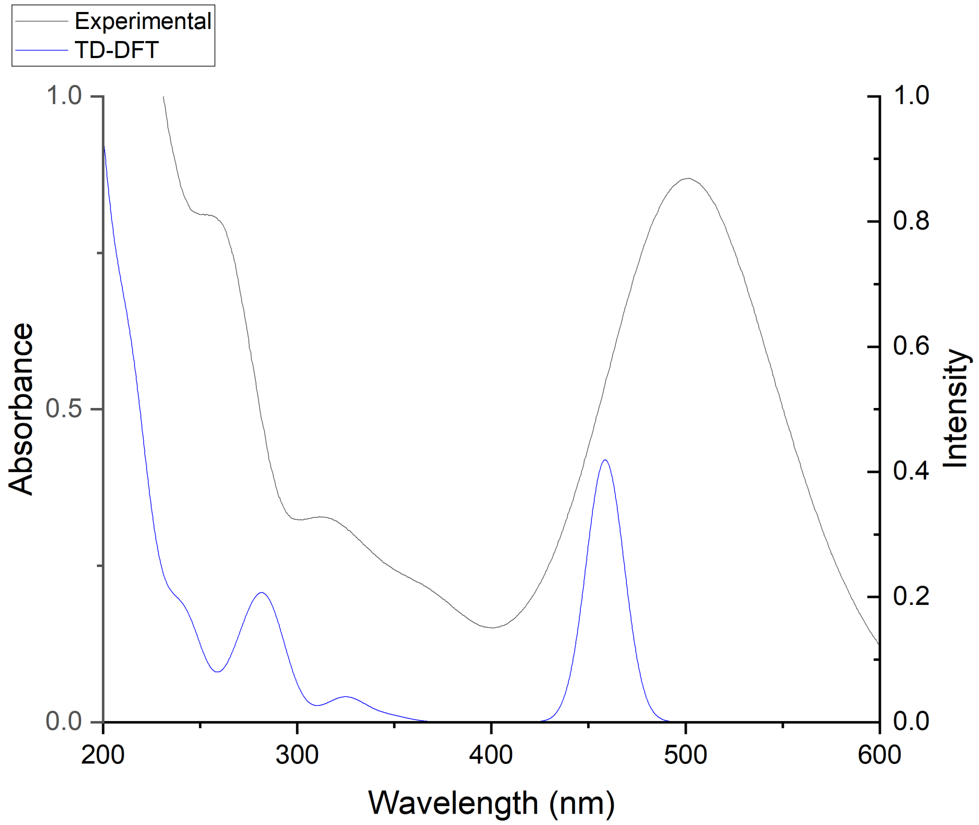

Figure S8. The predicted (TD-DFT) uv-vis spectrum for **3** (blue) in the gauche *cis,trans* conformation at M062X/Def2SVP including Gaussian broadening with band width on half height equal to 23 nm and the experimental uv-vis spectrum of **3** in *n*-pentane at 0.392 × 10^{-3} mol dm^{-3}

Table S6. Summary of the TD-DFT output for phosphabora-[*3*]dendralene **3** in the quasi *trans,trans* conformation at M062X/Def2SVP.

Wavelength (nm)	Energy (eV)	Transition	Oscillator Strength
**447.43**	2.771	HOMO to LUMO	0.423
**344.88**	3.595	HOMO to LUMO+2	0.001
**318.69**	3.890	HOMO-1 to LUMO	0.028
**283.72**	4.370	HOMO-2 to LUMO	0.149
**240.61**	5.153	HOMO-1 to LUMO+2	0.201

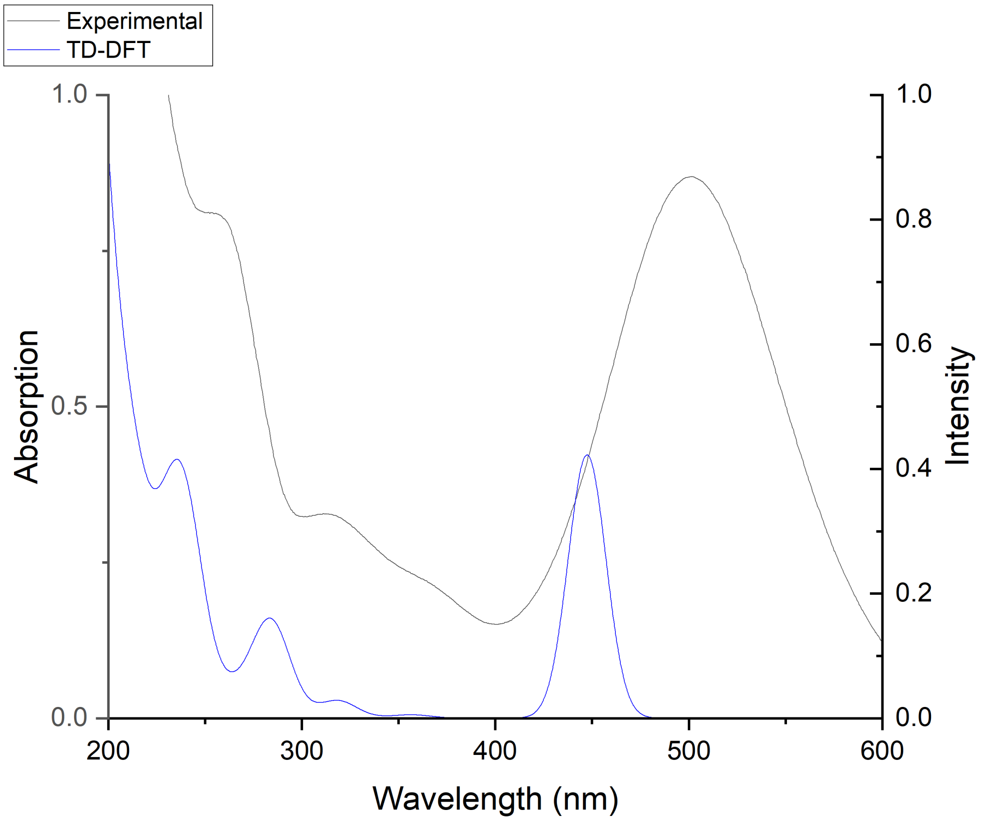

Figure S9. The Predicted (TD-DFT) uv-vis spectrum for **3** (blue) in the quasi *trans,trans* conformation at M062X/Def2SVP including Gaussian broadening with band width on half height equal to 23 nm and the experimental uv-vis spectrum of **3** in *n*-pentane at 0.392×10^{-3} mol dm^{-3}

3.5 Relative energies of parent [*3*]Dendralene conformations

Table S7. The sums of electronic and thermal energies and their normalised values for [3]dendralene at the MO62X/def2SVP level of theory.

[*3*]Dendralene Conformation	Sum of Electronic and Thermal Energies (H p^{-1})	Normalised Sum of Electronic and Thermal Energies (kJ mol^{-1})
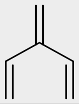	-232.96818	0.000
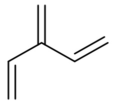	-232.96804	0.354
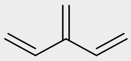	-232.96579	6.198

3.6 Conformational study of a model Phosphabora-[3]dendralene

Table S8. Absolute and relative energies an for every conformation of a model phosphabora-[3]dendralene at the MO62X/def2SVP level of theory.

**Model Compound Conformation**	**Sum of electronic and thermal energies (H p^{-1})**	**Normalised sum of electronic and thermal energies (kJ mol^{-1})**
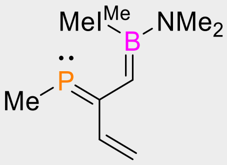	-1077.585139	0.000
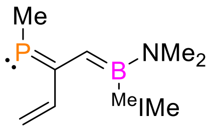	-1077.584858	0.731
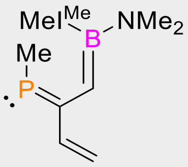	-1077.584735	1.050
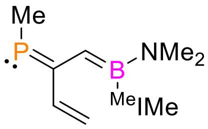	-1077.584114	2.665
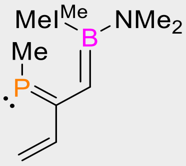	-1077.581529	9.386
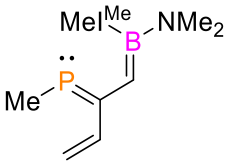	-1077.580805	11.268
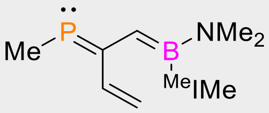	-1077.57889	16.247
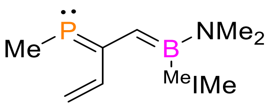	-1077.577147	20.779

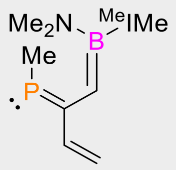	-1077.576967	21.247
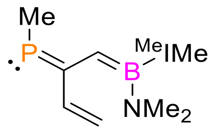	-1077.574739	27.040
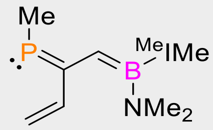	-1077.574119	28.652
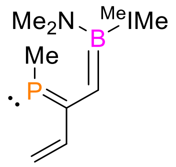	-1077.573578	30.059
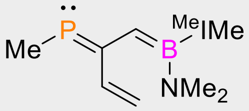	-1077.570242	38.732
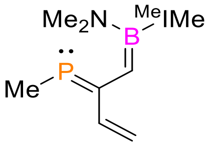	-1077.567225	46.576
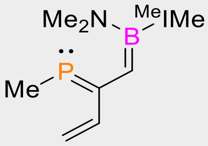	-1077.566939	47.320
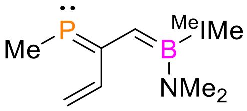	-1077.563298	56.787

3.7 Barrier to racemisation of 4

Table S9. The sums of electronic and thermal energies and their normalised values for the two diastereomers of **4** and the transition state for inversion at P, calculated at the MO62X/def2SVP level of theory.

Species	Sum of Electronic and Thermal Energies (Hartrees)	Normalised Sum of Electronic and Thermal Energies (kcal mol^{-1})	i
**4-*S*,*R***	-2701.6893	0.000	-
**4-TS**	-2701.6488	25.47	-206.10 cm^{-1}
**4-*R*,*R***	-2701.671	11.51	-

4. 2D NOESY, 1D Selective NOESY NMR and Interproton Distances Calculations

4.1 Compound 2

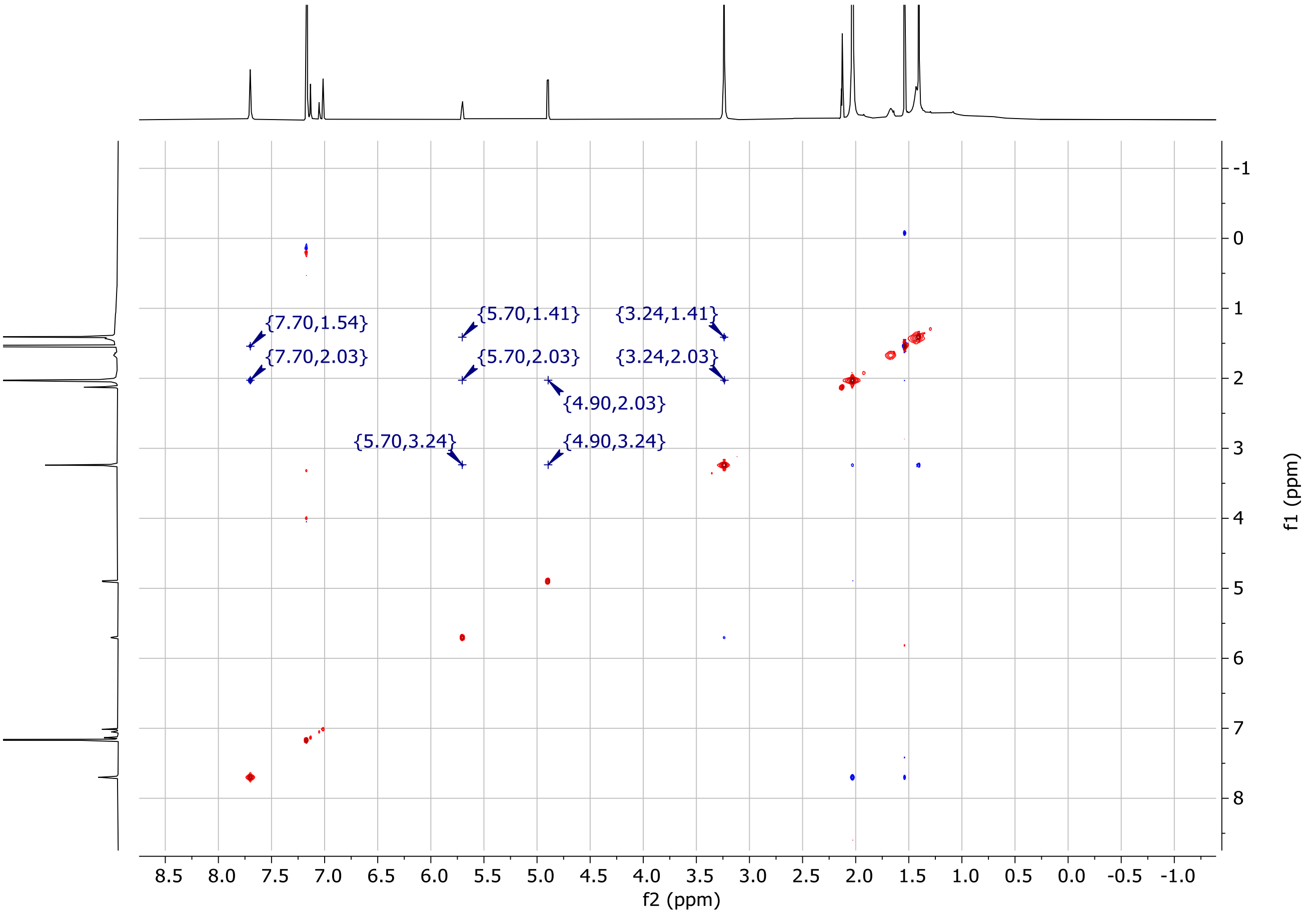

Figure S10. 2D NOESY NMR spectrum of **2** (601 MHz, 300 K, Toluene-d8, mixing time 0.4 s)

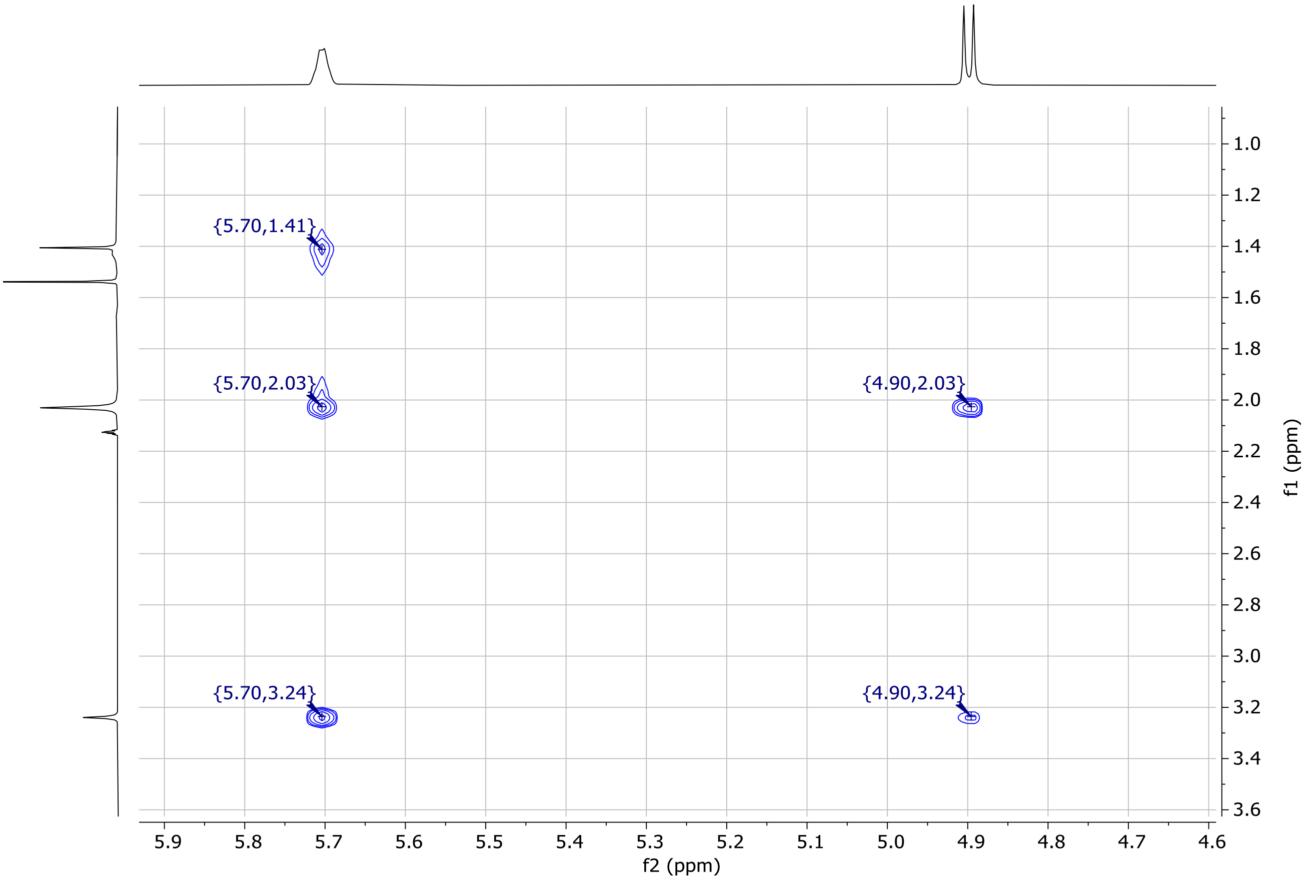

Figure S11. Expansion of (4.6-6.2, 1.0-3.5) ppm of the above 2D NOESY NMR spectrum of **2**

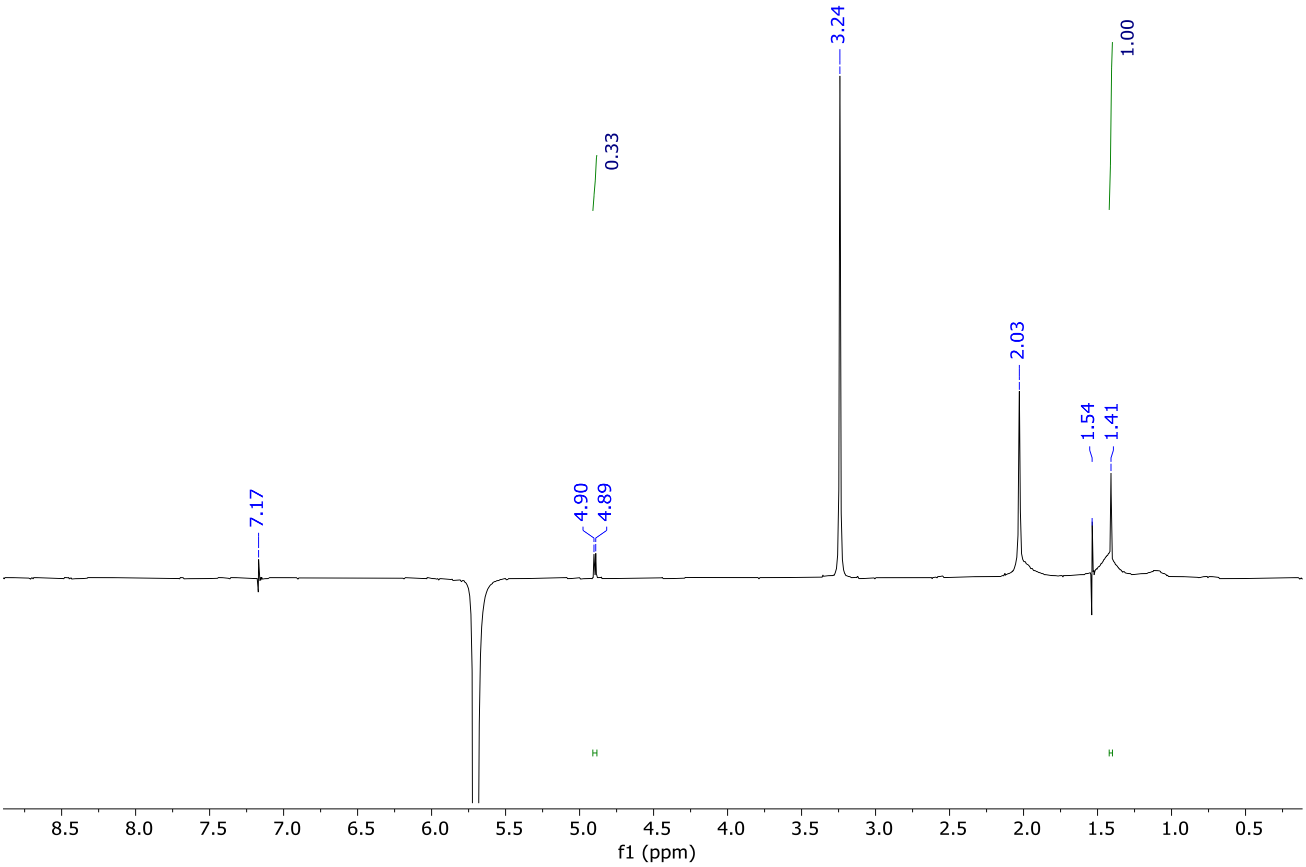

Figure S12. Selective 1D NOESY NMR spectrum of **2** (exciting at 5.66 ppm, mixing time 0.6 s, 601 MHz, 300 K, Toluene-d8)

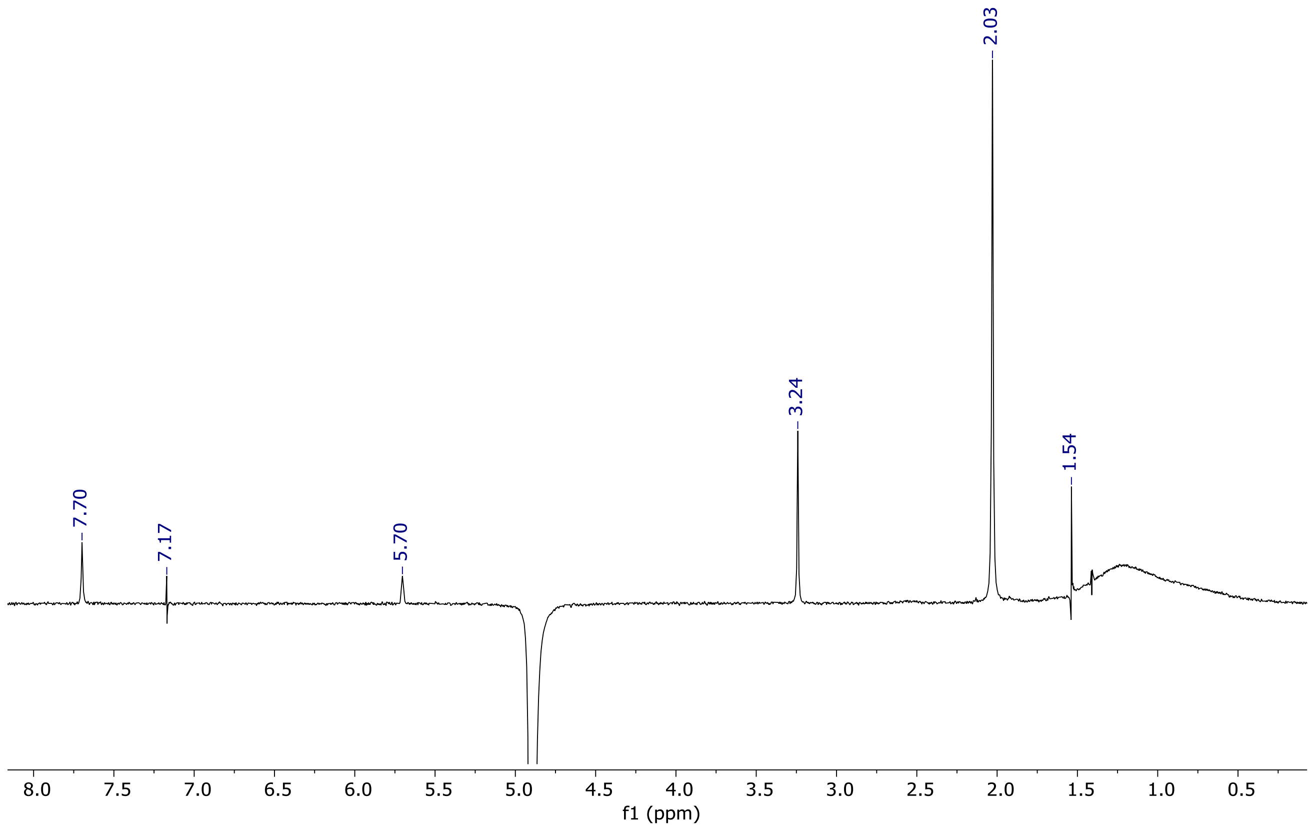

Figure S13. Selective 1D NOESY NMR spectrum of **2** (exciting at 4.85 ppm, mixing time 0.6 s, 601 MHz, 300 K, Toluene-d_8)

4.2 Compound 3

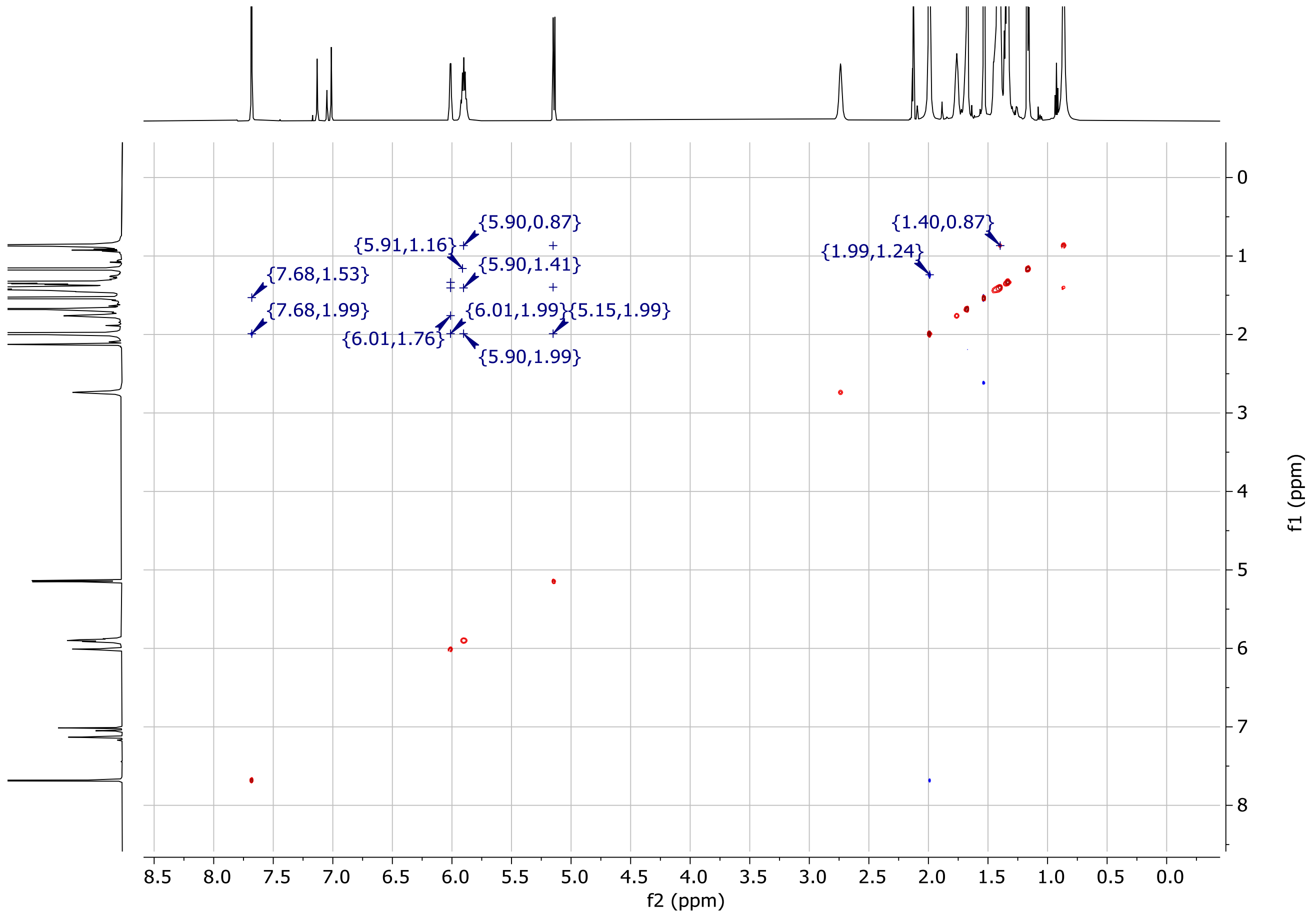

Figure S14. The 2D NOESY NMR spectrum of **3** (601 MHz, 300 K, Toluene-d8, mixing time 0.4 s)

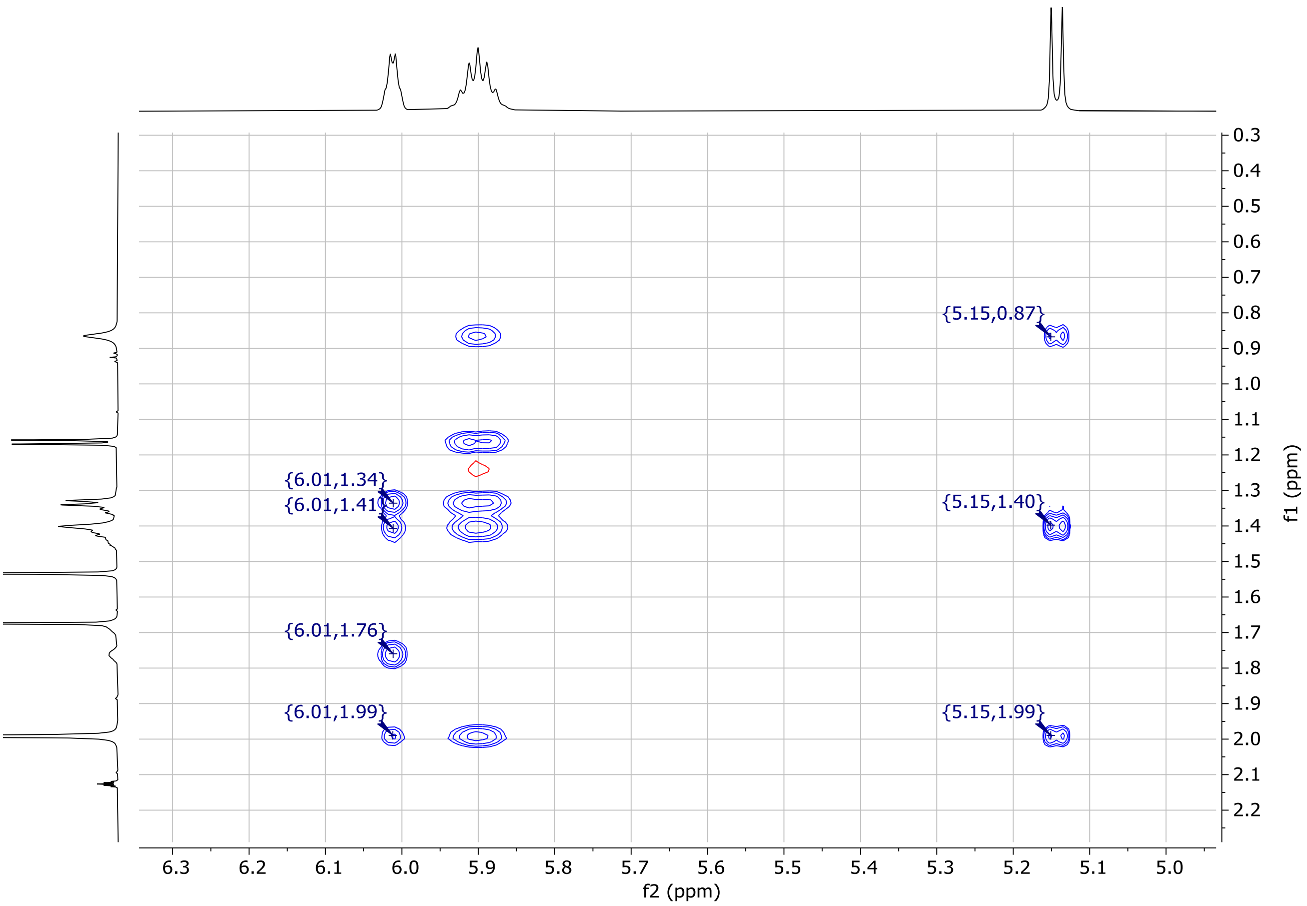

Figure S15. Expansion of (4.8-6.3, 0.6-2.1) ppm of the of the above 2D NOESY NMR spectrum of **3**

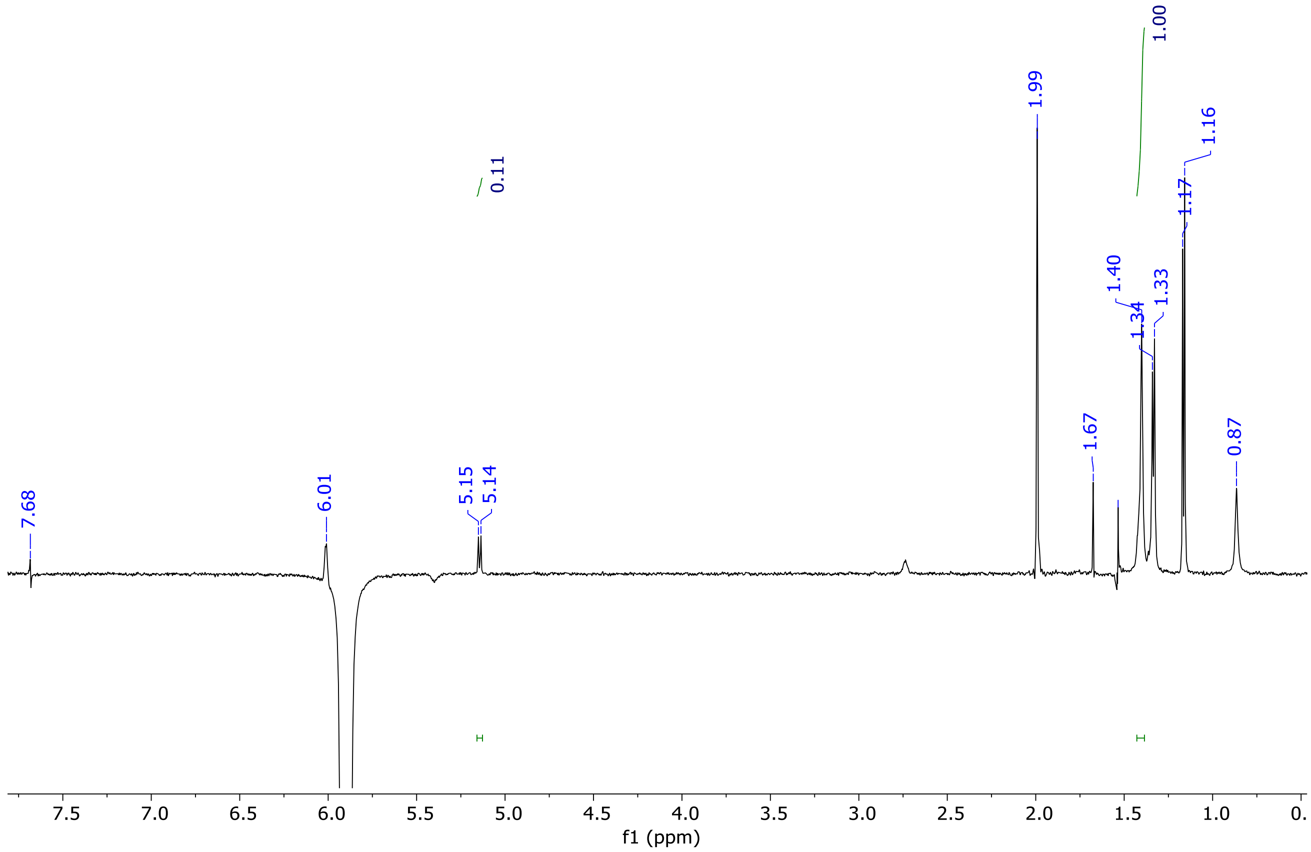

Figure S16. Selective 1D NOESY NMR spectrum of **3** (exciting at 5.97 ppm, mixing time 0.6 s, 601 MHz, 300 K, Toluene-d_8)

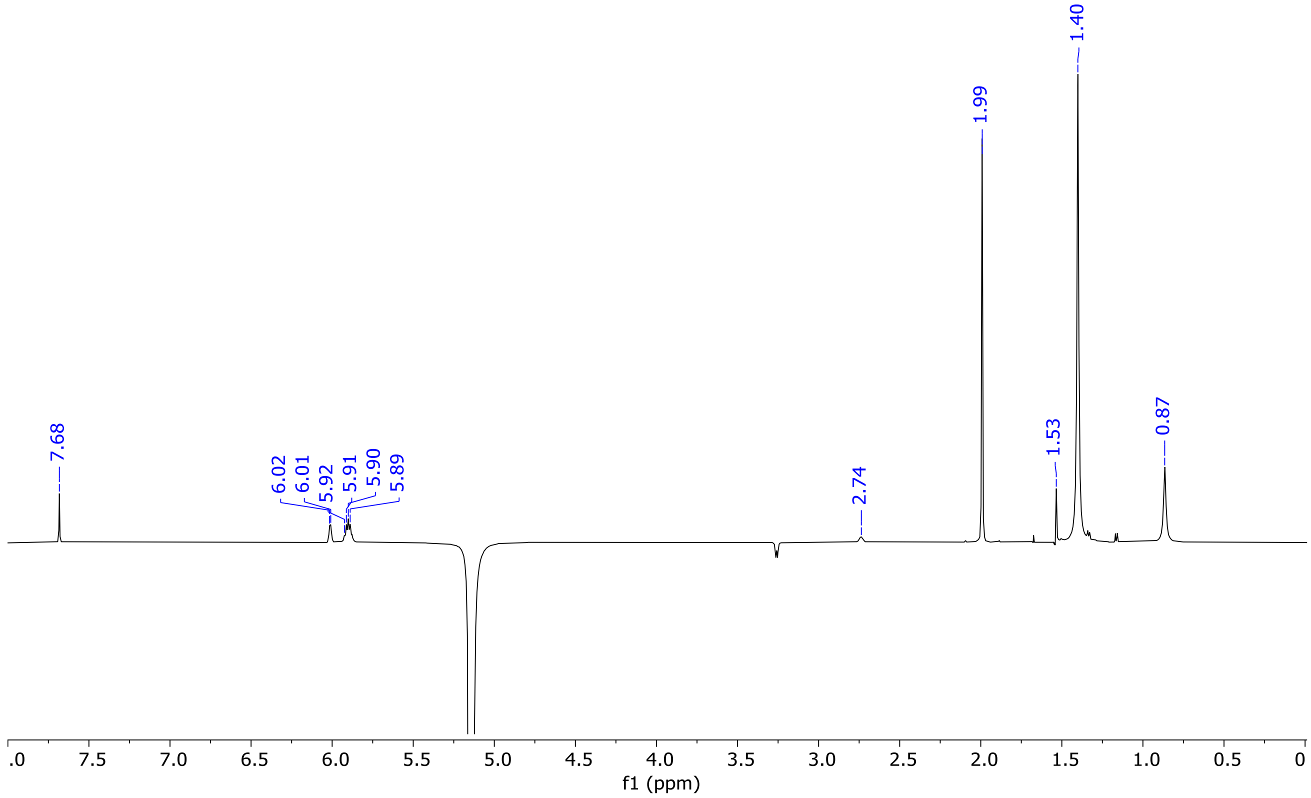

Figure S17. Selective 1D NOESY NMR spectrum of **3** (exciting at 5.10, mixing time 0.6 s, 601 MHz, 300 K, Toluene-d_8)

4.3 Interproton Distance Estimations for 2 and 3

Table S10. Calculated C=C**H**/B=C**H** interproton distances at the M062X/Def2SVP level of theory for **2** and **3** in the gauche *cis,trans* and quasi *trans,trans* conformations.

Conformation	2	3
gauche *cis-trans*	4.663 Å	4.695 Å
quasi trans-trans	3.500 Å	3.480 Å

The relationship between interproton distances and the observed NOE integrals is described by equation 1, where r_{ij} is the distance between the irradiated proton and the proton of interest, r_{ref} is a known interproton distance, a_{ref} is the integral area of the NOE corresponding to the known distance and a_{ij} is the integral area of the NOE corresponding to the distance to be measured.[5]

$$r_{ij} = r_{ref}\left(\frac{a_{ref}}{a_{ij}}\right)^{\frac{1}{6}} \quad (1)$$

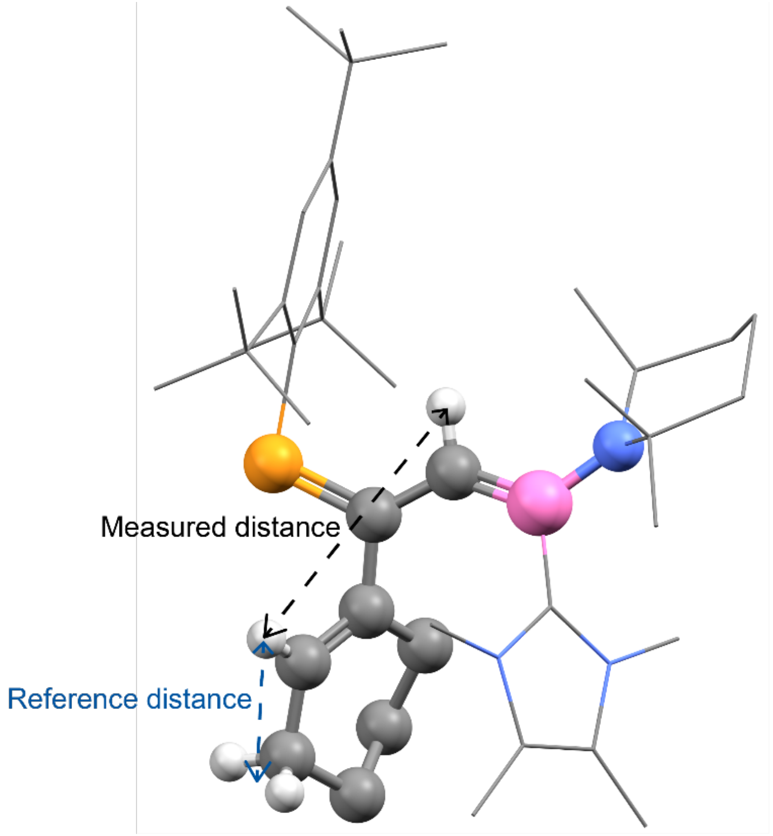

Figure S18. An example of the measured interproton distance (r_{ij}) and reference interproton distance (r_{ref}) in **2**

Table S11. The reference interproton distance (r_{ref}), NOE integral area corresponding to the reference distance (a_{ref}), NOE integral corresponding to the measured distance (r_{ij}) and the measured distance (r_{ij}) in **2** and **3**.

Compound	r_{ref} (Å)	a_{ref}	a_{ij}	r_{ij} (Å)
**2**	2.5695	1.00	0.33	3.091
**3**	2.5485	1.00	0.11	3.682

5. Variable Temperature NMR studies

5.1 Compound 2

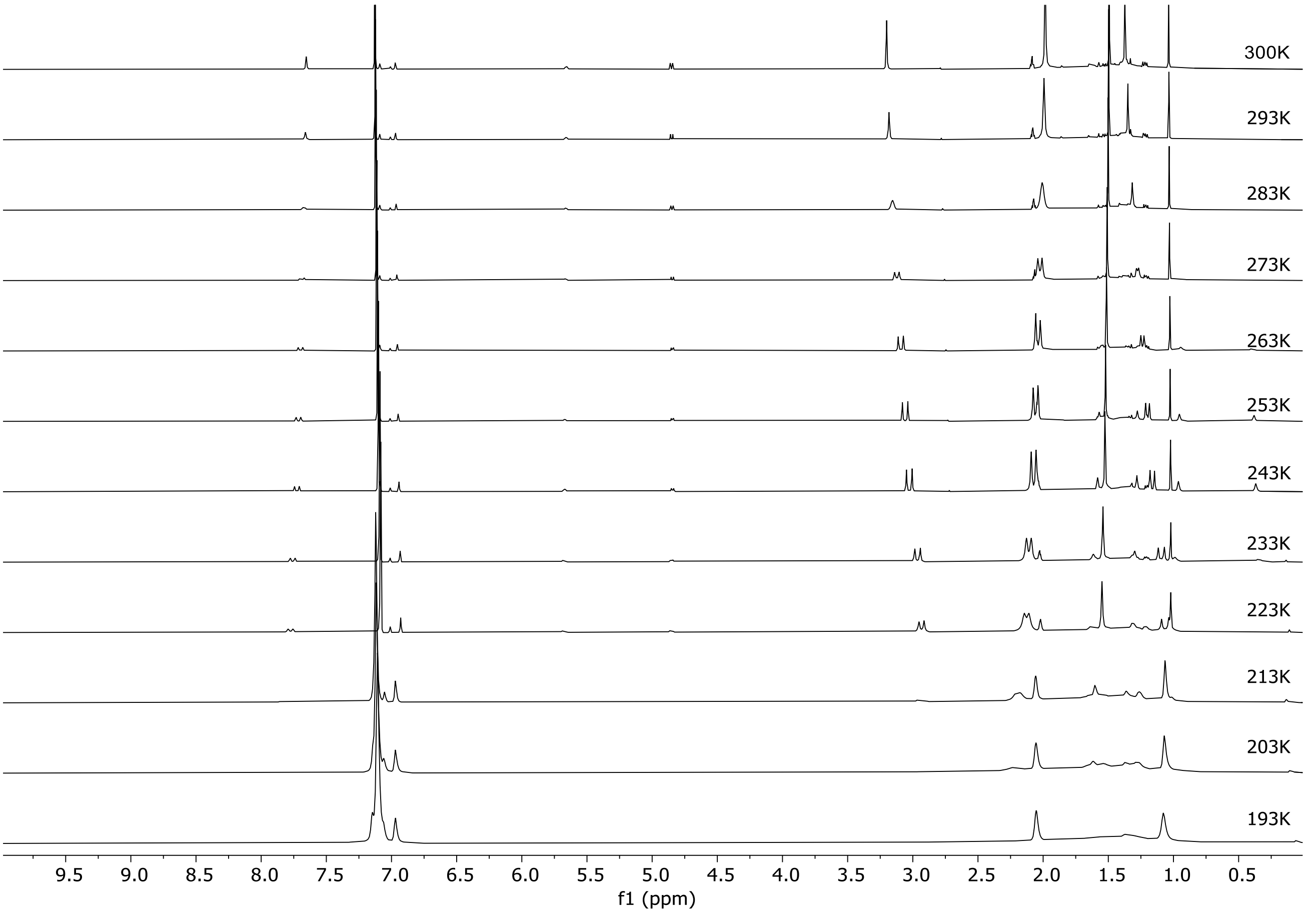

Figure S19. ^{1}H VT-NMR spectrum of **2** (500.2 MHz, 300 K to 193 K, Toluene-d_8)

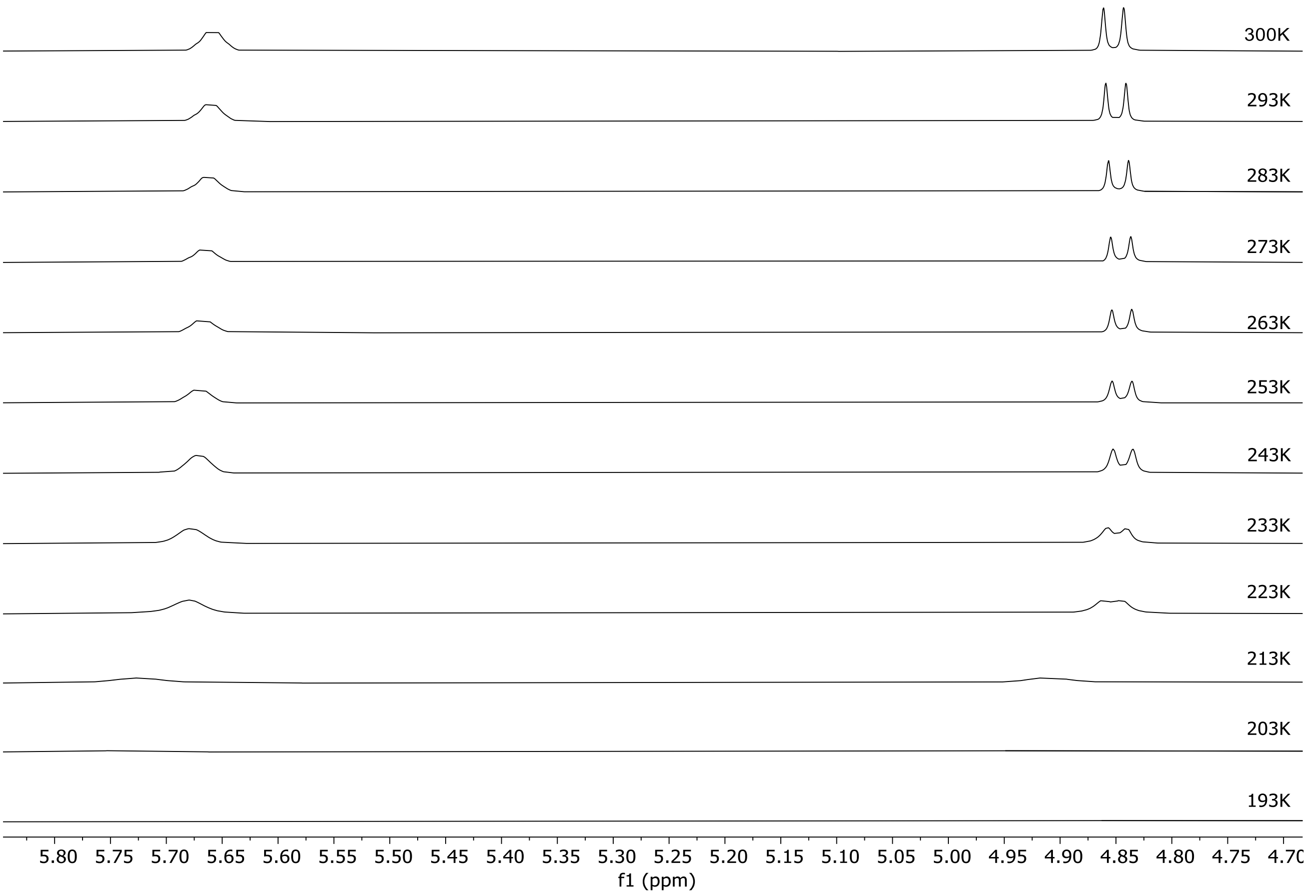

Figure S20. Expansion (5.90 to 4.70 ppm) of above ^{1}H VT-NMR spectrum of **2** (500.2 MHz, 300 K to 193 K, Toluene-d_8)

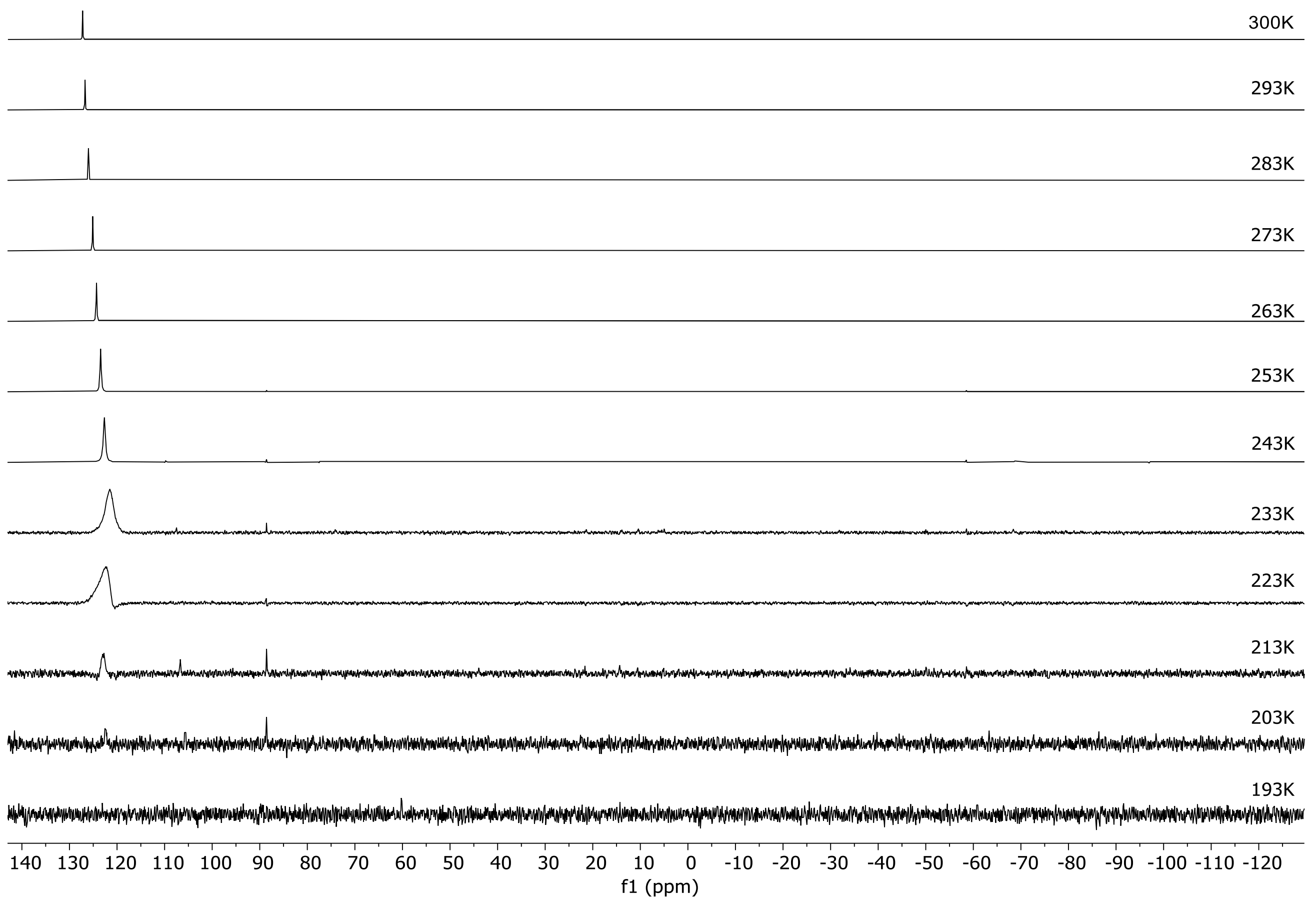

Figure S21. $^{31}P\{^{1}H\}$ VT-NMR spectra of **2** (202.5 MHz, 300 K to 193 K, Toluene-d_8)

5.2 Compound 3

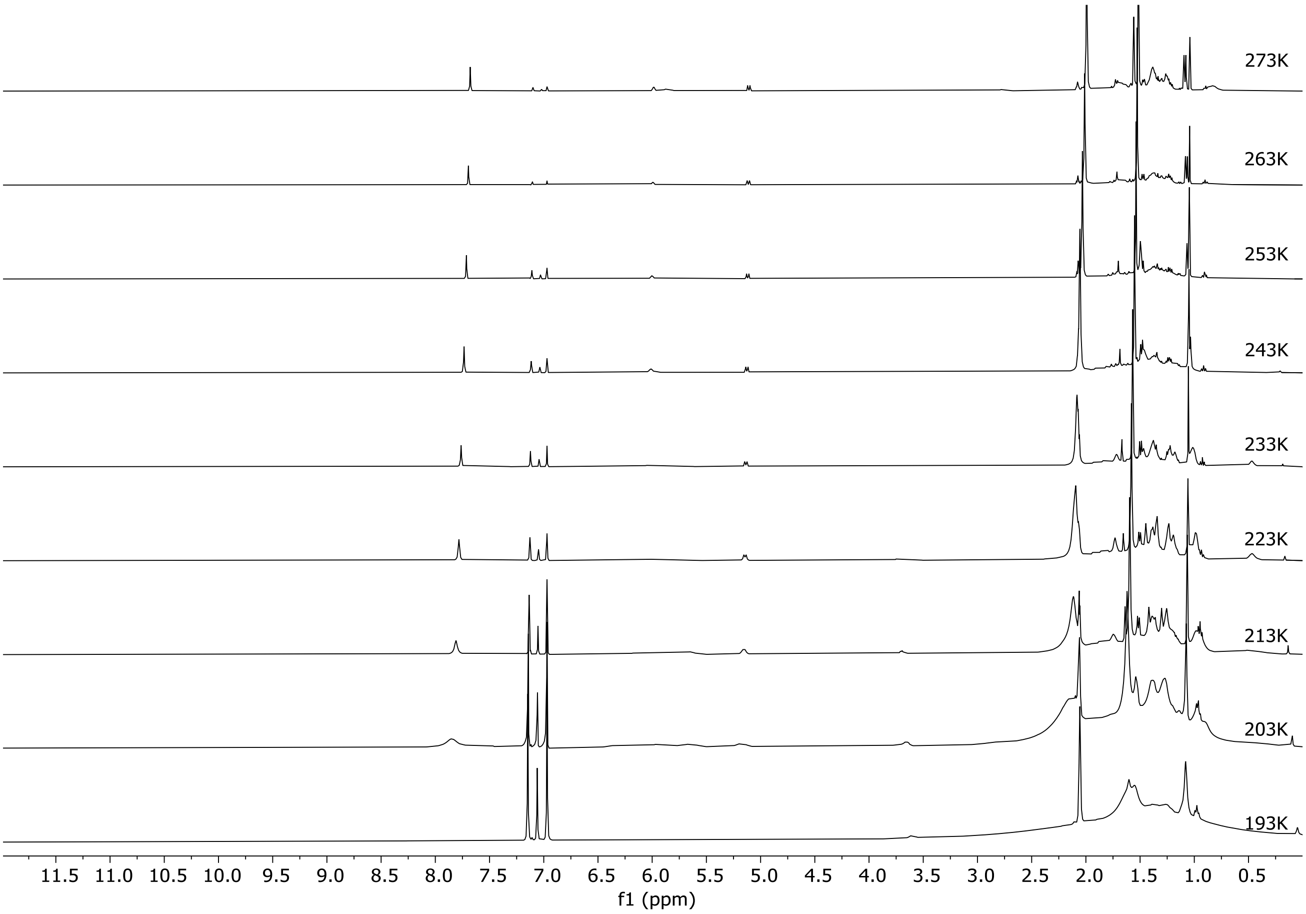

Figure S22. ^{1}H VT-NMR spectrum of **3** (500.2 MHz, 273 K to 193 K, Toluene-d_8)

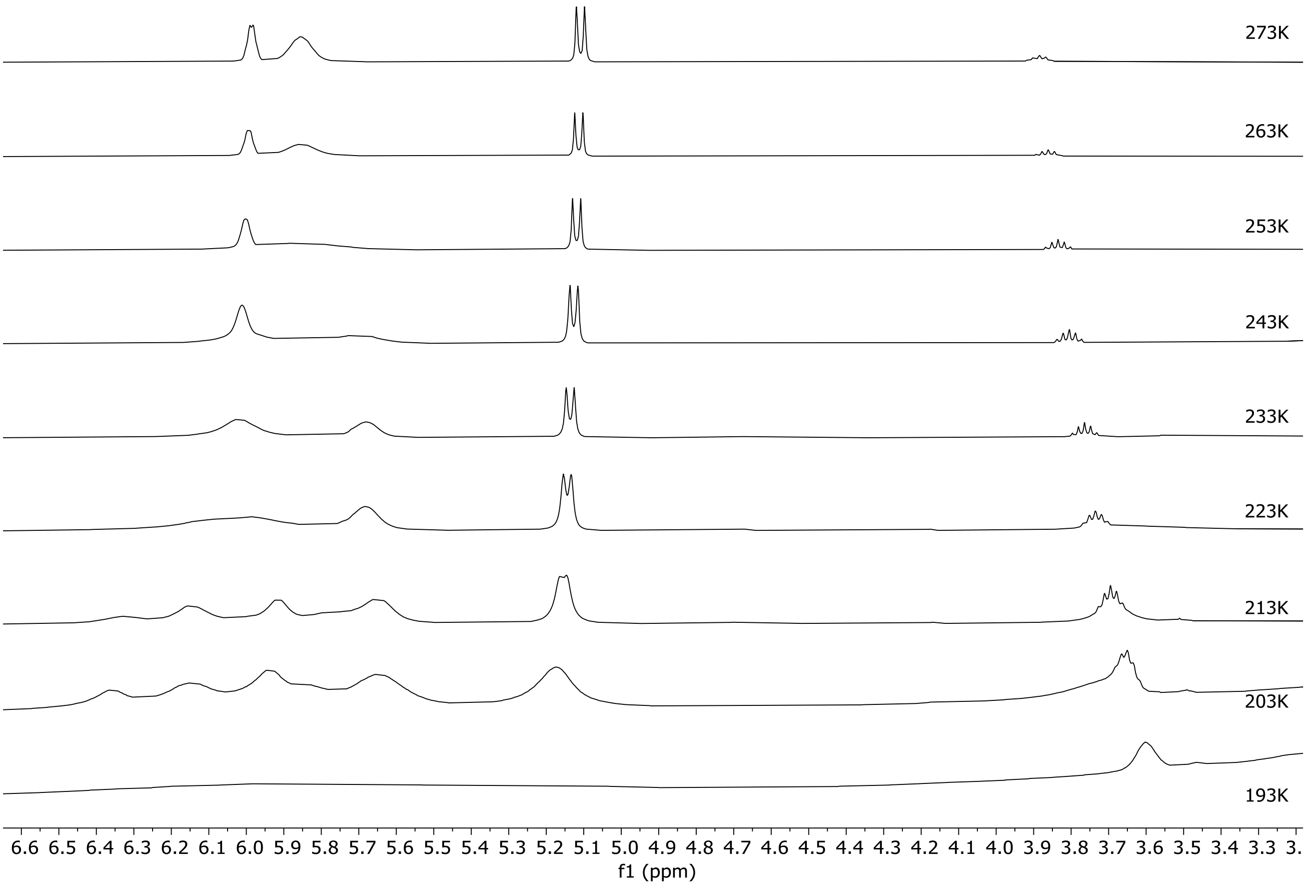

Figure S23. Expansion (6.6 to 3.2 ppm) of above ^{1}H VT-NMR spectrum of **3** (500.2 MHz, 273 K to 193 K, Toluene-d_8)

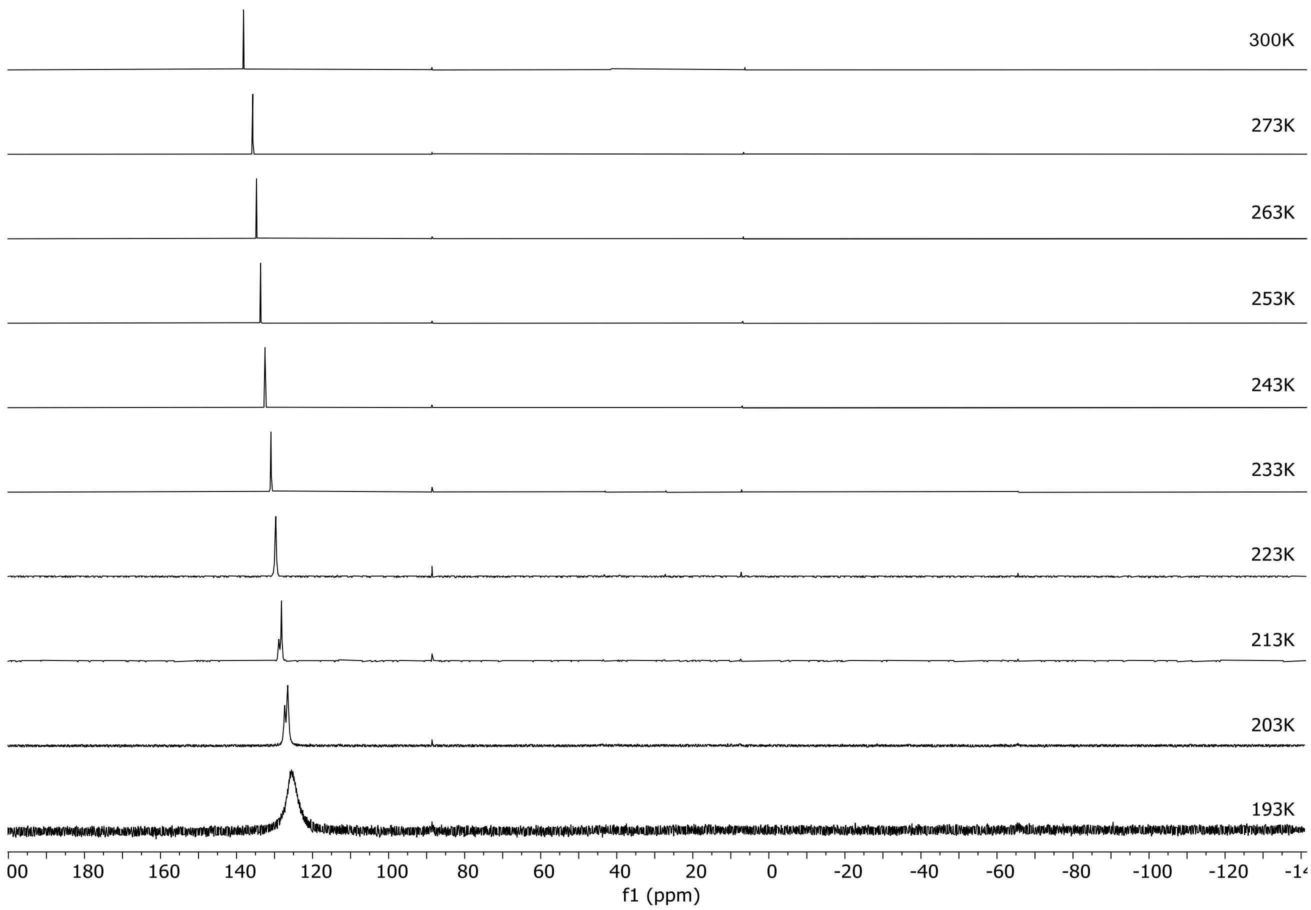

Figure S24. $^{31}P\{^{1}H\}$ VT-NMR spectrum of **3** (202.5 MHz, 300 K to 193 K, Toluene-d_8)

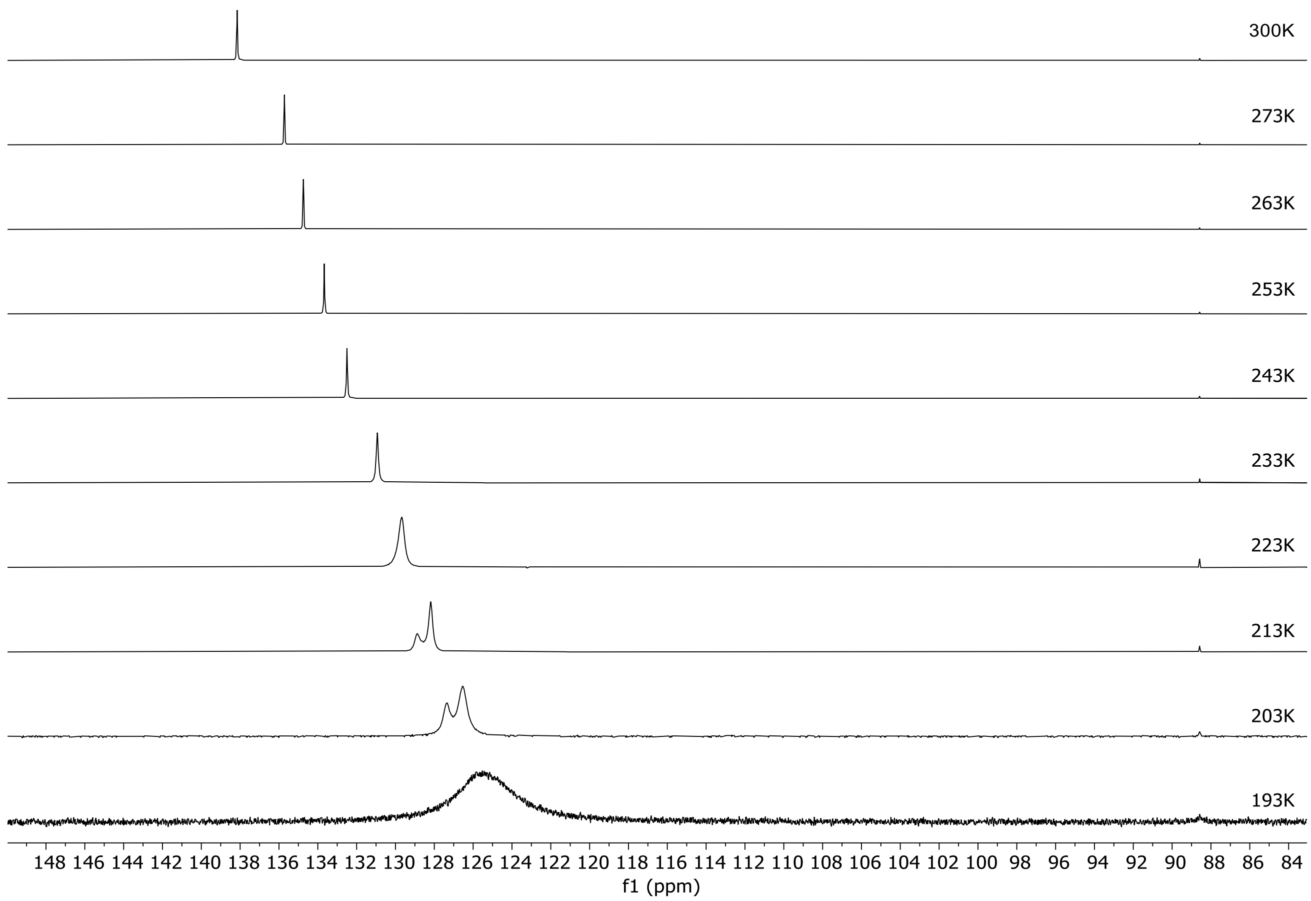

Figure S25. Expansion (150 to 83 ppm) of above $^{31}P\{^{1}H\}$ VT-NMR spectra of **3** (202.5 MHz, 300 K to 193 K, Toluene-d_8

5.3 Compound 4

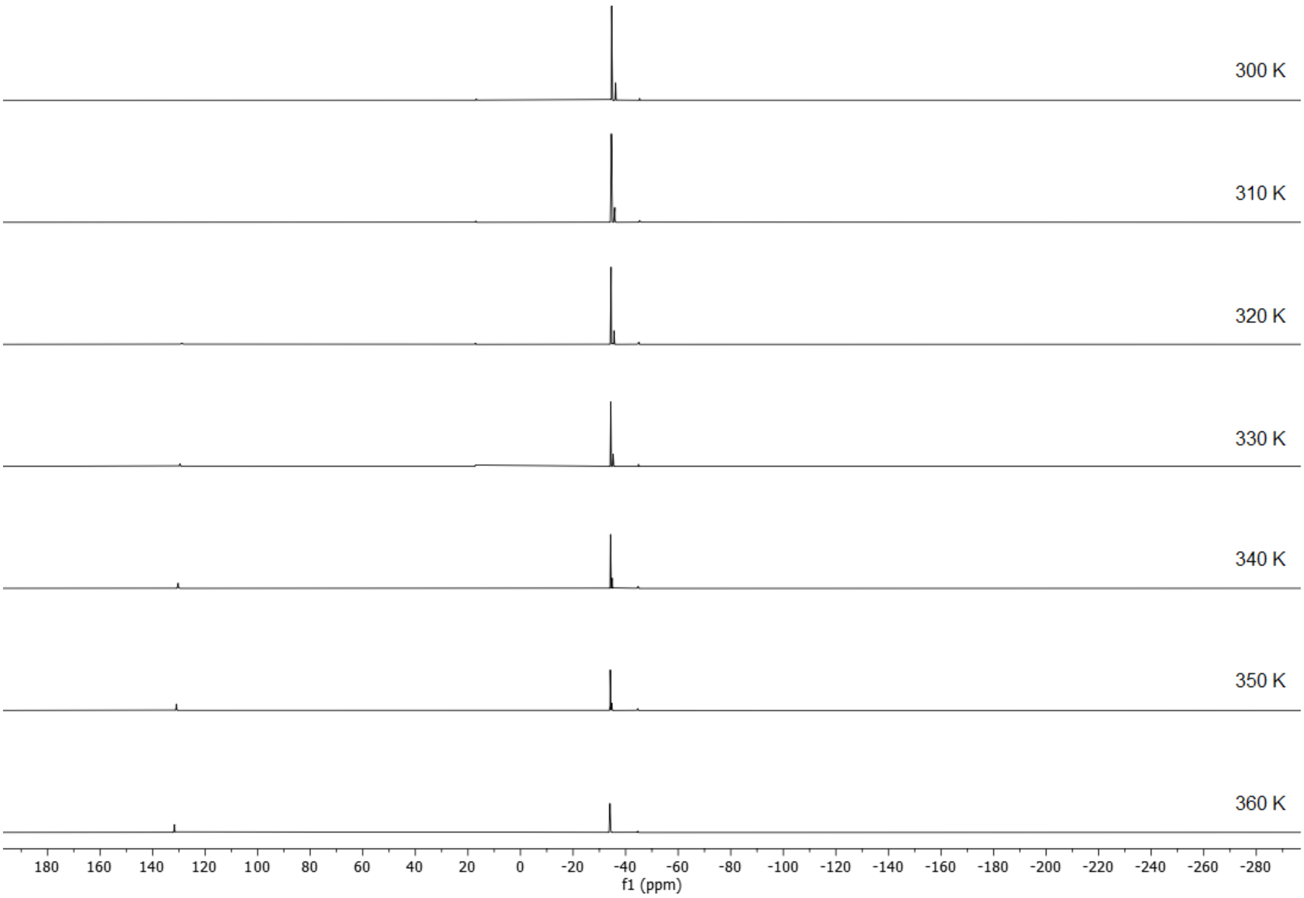

Figure S26. $^{31}P\{^{1}H\}$ VT-NMR spectrum of **4** (202.5 MHz, 300 K to 360 K, Toluene-d$_8$)

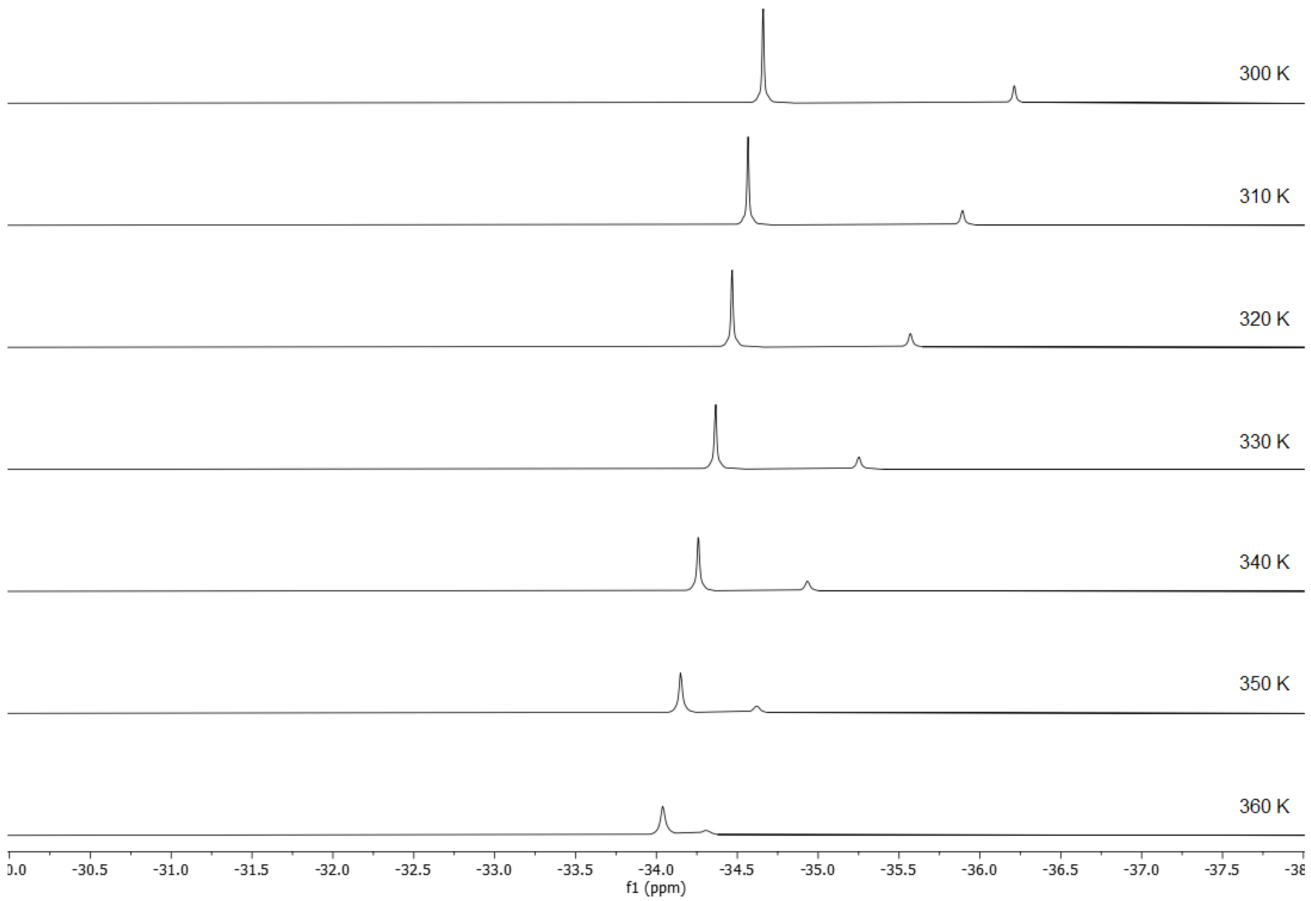

Figure S27. Expansion (-30 to -38 ppm) of above $^{31}P\{^1H\}$ VT-NMR spectrum of **4** (202.5 MHz, 300 K to 360 K, Toluene-d_8)

6. X-Ray Crystallographic Information

6.1 Experimental and Crystal Data for 1

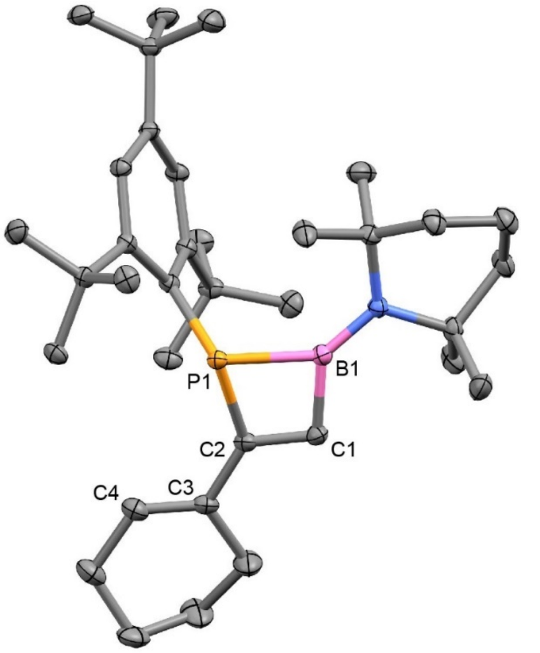

Experimental. Single translucent yellow block-shaped crystals of **1** were obtained by recrystallisation from hexane by slow cooling. A suitable crystal with dimensions 0.30 × 0.16 × 0.13 mm^3 was selected and mounted on a MITIGEN holder in Paratone oil on a Rigaku Oxford Diffraction SuperNova diffractometer. The crystal was kept at a steady T = 120.00(10) K during data collection. The structure was solved with the **ShelXS** (Sheldrick, 2008) solution program using direct methods and by using **Olex2** 1.5-beta (Dolomanov et al., 2009) as the graphical interface. The model was refined with **ShelXL** 2018/3 (Sheldrick, 2015) using full matrix least squares minimisation on $\boldsymbol{F}^2$.

Crystal Data. $C_{35}H_{57}BNP$, M_r = 533.59, monoclinic, $P2_1/n$ (No. 14), a = 11.7734(2) Å, b = 15.0615(3) Å, c = 18.3021(4) Å, β = 95.857(2)°, $\alpha = \gamma$ = 90°, V = 3228.48(11) Å^3, T = 120.00(10) K, Z = 4, Z' = 1, μ(Mo K_α) = 0.108, 82143 reflections measured, 9846 unique (R_{int} = 0.0577) which were used in all calculations. The final wR_2 was 0.1155 (all data) and R_1 was 0.0467 (I≥2 σ(I)).

Compound	1
Formula	$C_{35}H_{57}BNP$
$D_{calc.}$/ g cm^{-3}	1.098
μ/mm^{-1}	0.108
Formula Weight	533.59
Colour	translucent yellow
Shape	block-shaped
Size/mm^3	0.30×0.16×0.13
T/K	120.00(10)
Crystal System	monoclinic
Space Group	$P2_1/n$
a/Å	11.7734(2)
b/Å	15.0615(3)
c/Å	18.3021(4)
α/°	90
β/°	95.857(2)
γ/°	90
V/Å^3	3228.48(11)
Z	4
Z'	1
Wavelength/Å	0.71073
Radiation type	Mo K_α

$\Theta_{min}/^\circ$	3.216	Refl's I≥2 σ(I)	8170
$\Theta_{max}/^\circ$	30.506	R_{int}	0.0577
Measured Refl's.	82143	Parameters	375
Indep't Refl's	9846		

Restraints	5
Largest Peak	0.398
Deepest Hole	-0.251
GooF	1.048
wR_2 (all data)	0.1155
wR_2	0.1090
R_1 (all data)	0.0595
R_1	0.0467

Structure Quality Indicators

Reflections:	d min (Mo) 2Θ=61.0° 0.70	I/σ(I) 28.0	Rint 5.77%	Full 50.5° 99.8
Refinement:	Shift 0.001	Max Peak 0.4	Min Peak -0.2	GooF 1.048

A translucent yellow block-shaped-shaped crystal with dimensions 0.30 × 0.16 × 0.13 mm^3 was mounted on a MITIGEN holder in Paratone oil. Data were collected using a Rigaku Oxford Diffraction SuperNova diffractometer equipped with an Oxford Cryosystems Cryostream 700+ low-temperature device operating at T = 120.00(10) K.

Data were measured using ω scans with Mo K_α radiation. The diffraction pattern was indexed and the total number of runs and images was based on the strategy calculation from the program CrysAlisPro 1.171.41.123a (Rigaku OD, 2022). The maximum resolution that was achieved was Θ = 30.506° (0.70 Å).

The unit cell was refined using CrysAlisPro 1.171.41.123a (Rigaku OD, 2022) on 20434 reflections, 25% of the observed reflections.

Data reduction, scaling and absorption corrections were performed using CrysAlisPro 1.171.41.123a (Rigaku OD, 2022). The final completeness is 99.80 % out to 30.506° in Θ. A multi-scan absorption correction was performed using CrysAlisPro 1.171.41.123a (Rigaku Oxford Diffraction, 2022) Spherical absorption correction using equivalent radius and absorption coefficient. Empirical absorption correction using spherical harmonics, implemented in SCALE3 ABSPACK scaling algorithm. The absorption coefficient μ of this material is 0.108 mm^{-1} at this wavelength (λ = 0.71073Å) and the minimum and maximum transmissions are 0.983 and 0.983.

The structure was solved and the space group $P2_1/n$ (# 14) determined by the ShelXS (Sheldrick, 2008) structure solution program using direct methods and refined by full matrix least squares minimisation on $\boldsymbol{F}^2$ using version 2018/3 of **ShelXL** 2018/3 (Sheldrick, 2015). All non-hydrogen atoms were refined anisotropically. Hydrogen atom positions were calculated geometrically and refined using the riding model. Hydrogen atom positions were calculated geometrically and refined using the riding model.

_refine_special_details: C6 and C7 were modelled as disordered over two sites, consistent with peaks in a difference map.

There is a single molecule in the asymmetric unit, which is represented by the reported sum formula. In other words: Z is 4 and Z' is 1.

6.2 Experimental and Crystal Data for 2

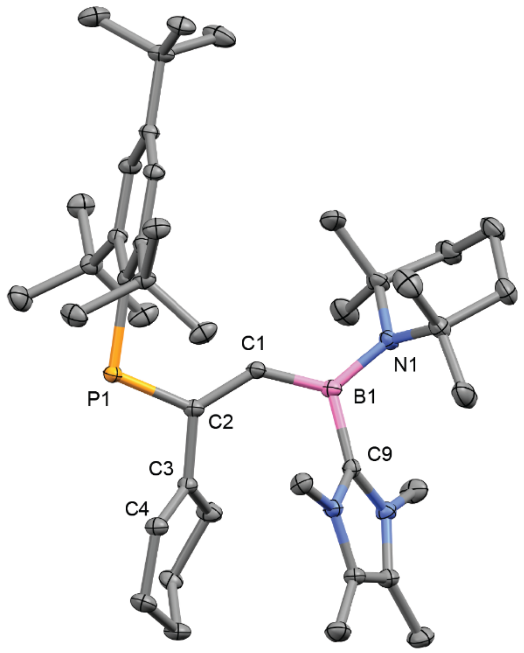

Experimental. Single red prism-shaped crystals of **2** were obtained by a recrystallization from benzene by slow evaporation. A suitable crystal with dimensions 0.34 × 0.23 × 0.07 mm^3 was selected and mounted on a MITIGEN holder in Paratone oil on a Bruker D8 VENTURE diffractometer. The crystal was kept at a steady T = 100.00 K during data collection. The structure was solved with the **ShelXT** 2018/2 (Sheldrick, 2018) solution program using dual methods and by using **Olex2** 1.5-beta (Dolomanov et al., 2009) as the graphical interface. The model was refined with **ShelXL** 2018/3 (Sheldrick, 2015) using full matrix least squares minimisation on $\boldsymbol{F^2}$.

Crystal Data. $C_{57}H_{84}BN_3P$, M_r = 868.14, triclinic, P-1 (No. 2), a = 11.8846(12) Å, b = 14.2257(15) Å, c = 16.9871(17) Å, α = 78.469(4)°, β = 74.673(4)°, γ = 69.801(4)°, V = 2580.7(5) Å^3, T = 100.00 K, Z = 2, Z' = 1, $\mu(MoK_\alpha)$ = 0.092, 105095 reflections measured, 15611 unique (R_{int} = 0.0442) which were used in all calculations. The final wR_2 was 0.1066 (all data) and R_1 was 0.0410 (I≥2 σ(I)).

Compound	**2**
Formula	$C_{57}H_{84}BN_3P.2.5C_6H_6$
$D_{calc.}$/ g cm^{-3}	1.117
μ/mm^{-1}	0.092
Formula Weight	868.14
Colour	red
Shape	prism-shaped
Size/mm^3	0.34×0.23×0.07
T/K	100.00
Crystal System	triclinic
Space Group	P-1
a/Å	11.8846(12)
b/Å	14.2257(15)
c/Å	16.9871(17)
α/°	78.469(4)
β/°	74.673(4)
γ/°	69.801(4)
V/Å^3	2580.7(5)
Z	2
Z'	1
Wavelength/Å	0.71073
Radiation type	MoK_α
Θ_{min}/°	2.095
Θ_{max}/°	30.487
Measured Refl's.	105095
Indep't Refl's	15611
Refl's I≥2 σ(I)	12731

R_{int}	0.0442
Parameters	890
Restraints	7
Largest Peak	0.359
Deepest Hole	-0.279
GooF	1.042
wR_2 (all data)	0.1066
wR_2	0.0965
R_1 (all data)	0.0562
R_1	0.0410

Structure Quality Indicators

Reflections:	d min (Mo) 2Θ=61.0° 0.70	I/σ(I) 31.9	Rint m=6.73 4.42%	Full 50.5° 99% to 61.0° 99.9
Refinement:	Shift 0.002	Max Peak 0.4	Min Peak -0.3	GooF 1.042

A red prism-shaped-shaped crystal with dimensions 0.34 × 0.23 × 0.07 mm^3 was mounted on a MITIGEN holder in Paratone oil. Data were collected using a Bruker D8 VENTURE diffractometer equipped with an Oxford Cryosystems Cryostream 800 low-temperature device operating at T = 100.00 K.

Data were measured using φ and ω scans with MoK_α radiation. The diffraction pattern was indexed and the total number of runs and images was based on the strategy calculation from the program APEX4. The maximum resolution that was achieved was Θ = 30.487° (0.70 Å).

The unit cell was refined using SAINT V8.40B (Bruker, 2016) on 9821 reflections, 9% of the observed reflections.

Data reduction, scaling and absorption corrections were performed using SAINT V8.40B (Bruker, 2016). The final completeness is 99.90 % out to 30.487° in Θ. SADABS-2016/2 (Bruker, 2016/2) was used for absorption correction. wR_2(int) was 0.1151 before and 0.0510 after correction. The Ratio of minimum to maximum transmission is 0.9631. The λ/2 correction factor is not present. The absorption coefficient μ of this material is 0.092 mm^{-1} at this wavelength (λ = 0.71073Å) and the minimum and maximum transmissions are 0.719 and 0.746.

The structure was solved and the space group P-1 (# 2) determined by the ShelXT 2018/2 (Sheldrick, 2018) structure solution program using dual methods and refined by full matrix least squares minimisation on $\boldsymbol{F}^2$ using version 2018/3 of **ShelXL** 2018/3 (Sheldrick, 2015). All non-hydrogen atoms were refined anisotropically. Hydrogen atom positions were calculated geometrically and refined using the riding model. Most hydrogen atom positions were calculated geometrically and refined using the riding model, but some hydrogen atoms were refined freely.

_refine_special_details: Disorder was modelled based on peaks in a difference map and refined with geometric and displacement ellipsoid restraints.

There is a single molecule in the asymmetric unit, which is represented by the reported sum formula. In other words: Z is 2 and Z' is 1.

6.3 Experimental and Crystal Data for 3

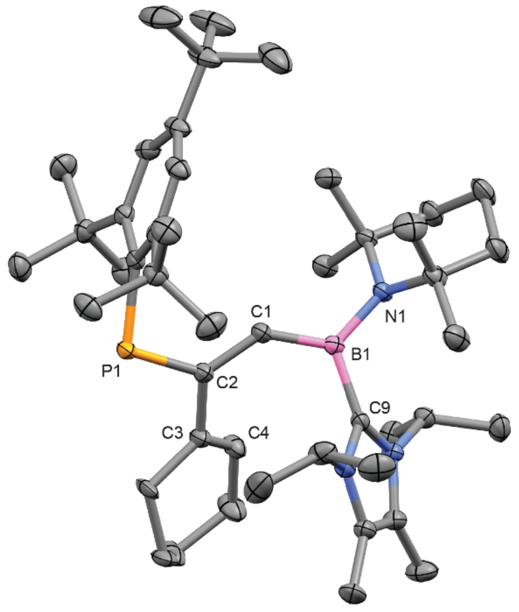

Experimental. Single black plate-shaped crystals of **3** recrystallised from hexane by slow evaporation. A suitable crystal with dimensions 0.29 × 0.13 × 0.04 mm^3 was selected and mounted on a mitegen tip in Paratone oil on a Rigaku Oxford Diffraction SuperNova diffractometer. The crystal was kept at a steady T = 120.0 K during data collection. The structure was solved with the **ShelXT** 2018/2 (Sheldrick, 2018) solution program using dual methods and by using **Olex2** 1.5-beta (Dolomanov et al., 2009) as the graphical interface. The model was refined with **ShelXL** 2018/3 (Sheldrick, 2015) using full matrix least squares minimisation on $\boldsymbol{F}^2$.

Crystal Data. $C_{46}H_{77}BN_3P$, M_r = 713.88, monoclinic, $P2_1/c$ (No. 14), a = 18.3315(2) Å, b = 12.67040(10) Å, c = 19.1824(2) Å, β = 93.3330(10)°, $\alpha = \gamma$ = 90°, V = 4447.91(8) Å^3, T = 120.0 K, Z = 4, Z' = 1, μ(Cu K$_\alpha$) = 0.776, 38880 reflections measured, 9161 unique (R_{int} = 0.0721) which were used in all calculations. The final wR_2 was 0.1636 (all data) and R_1 was 0.0580 (I≥2 σ(I)).

Compound	3
Formula	$C_{46}H_{77}BN_3P$
$D_{calc.}$/ g cm^{-3}	1.066
μ/mm^{-1}	0.776
Formula Weight	713.88
Colour	black
Shape	plate-shaped
Size/mm^3	0.29×0.13×0.04
T/K	120.0
Crystal System	monoclinic
Space Group	$P2_1/c$
a/Å	18.3315(2)
b/Å	12.67040(10)
c/Å	19.1824(2)
α/°	90
β/°	93.3330(10)
γ/°	90
V/Å^3	4447.91(8)
Z	4
Z'	1
Wavelength/Å	1.54184
Radiation type	Cu K$_\alpha$
Θ_{min}/°	4.184
Θ_{max}/°	75.860
Measured Refl's.	38880
Indep't Refl's	9161
Refl's I≥2 σ(I)	7901

R_{int}	0.0721
Parameters	507
Restraints	21
Largest Peak	0.729
Deepest Hole	-0.592
GooF	1.030
wR_2 (all data)	0.1636
wR_2	0.1539
R_1 (all data)	0.0654
R_1	0.0580

Structure Quality Indicators

Reflections:	d min (Cu\a) 2Θ=151.7° 0.80	I/σ(I) 20.5	Rint 7.21%	Full 135.4° 99% to 151.7° 99.9
Refinement:	Shift 0.000	Max Peak 0.7	Min Peak -0.6	GooF 1.030

A black plate-shaped-shaped crystal with dimensions 0.29 × 0.13 × 0.04 mm^3 was mounted on a mitegen tip in Paratone oil. Data were collected using a Rigaku Oxford Diffraction SuperNova diffractometer equipped with an Oxford Cryosystems Cryostream 700+ low-temperature device operating at T = 120.0 K.

Data were measured using ω scans with Cu K_{α} radiation. The diffraction pattern was indexed and the total number of runs and images was based on the strategy calculation from the program CrysAlisPro 1.171.41.123a (Rigaku OD, 2022). The maximum resolution that was achieved was Θ = 75.860° (0.80 Å).

The unit cell was refined using CrysAlisPro 1.171.41.123a (Rigaku OD, 2022) on 17798 reflections, 46% of the observed reflections.

Data reduction, scaling and absorption corrections were performed using CrysAlisPro 1.171.41.123a (Rigaku OD, 2022). The final completeness is 99.90 % out to 75.860° in Θ. A multi-scan absorption correction was performed using CrysAlisPro 1.171.41.123a (Rigaku Oxford Diffraction, 2022) Empirical absorption correction using spherical harmonics, implemented in SCALE3 ABSPACK scaling algorithm. The absorption coefficient μ of this material is 0.776 mm^{-1} at this wavelength (λ = 1.54184Å) and the minimum and maximum transmissions are 0.847 and 1.000.

The structure was solved and the space group $P2_1/c$ (# 14) determined by the ShelXT 2018/2 (Sheldrick, 2018) structure solution program using dual methods and refined by full matrix least squares minimisation on $\boldsymbol{F}^2$ using version 2018/3 of **ShelXL** 2018/3 (Sheldrick, 2015). All non-hydrogen atoms were refined anisotropically. Hydrogen atom positions were calculated geometrically and refined using the riding model. Hydrogen atom positions were calculated geometrically and refined using the riding model.

_refine_special_details: Atoms C26-C28 were modelled as disordered, consistent with peaks in a difference map. Displacement ellipsoid and geometric restraints were used.

There is a single molecule in the asymmetric unit, which is represented by the reported sum formula. In other words: Z is 4 and Z' is 1

6.4 Experimental and Crystal Data for 4

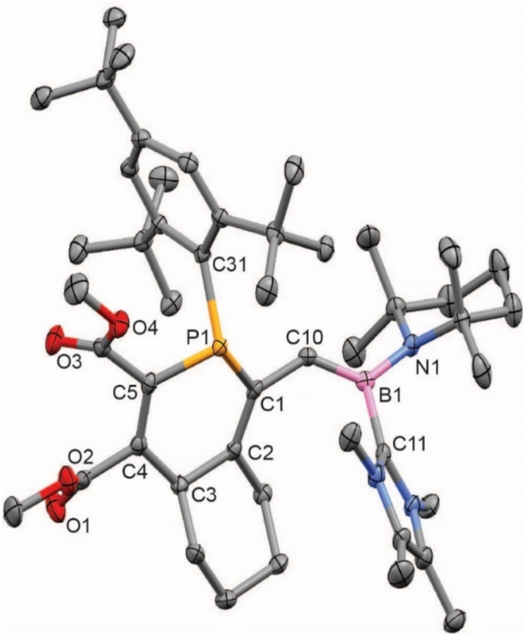

Experimental. Single red plate-shaped crystals of **4** were obtained by recrystallization from hexane and storage at room temperature for 2 days. A suitable crystal with dimensions 0.36 × 0.22 × 0.17 mm^3 was selected and mounted on a mitegen tip in Paratone oil on an Xcalibur, Eos diffractometer. The crystal was kept at a steady T = 120.0 K during data collection. The structure was solved with the **ShelXT** 2018/2 (Sheldrick, 2018) solution program using dual methods and by using **Olex2** 1.5-beta (Dolomanov et al., 2009) as the graphical interface. The model was refined with **ShelXL** 2018/3 (Sheldrick, 2015) using full matrix least squares minimisation on $\boldsymbol{F^2}$.

Crystal Data. $C_{51}H_{82}BN_3O_4P$, M_r = 842.97, monoclinic, $P2_1/n$ (No. 14), a = 21.4330(5) Å, b = 10.5389(2) Å, c = 24.3822(6) Å, β = 113.594(3)°, $\alpha = \gamma$ = 90°, V = 5047.1(2) $Å^3$, T = 120.0 K, Z = 4, Z' = 1 μ(Mo K_α) = 0.099, 118293 reflections measured, 9568 unique (R_{int} = 0.0634) which were used in all calculations. The final wR_2 was 0.1065 (all data) and R_1 was 0.0515 (I≥2 σ(I)).

Compound	4
Formula	$C_{51}H_{82}BN_3O_4P$. $0.5(C_6H_{14})$
$D_{calc.}$/ g cm^{-3}	1.109
μ/mm^{-1}	0.099
Formula Weight	842.97
Colour	red
Shape	plate-shaped
Size/mm^3	0.36×0.22×0.17
T/K	120.0
Crystal System	monoclinic
Space Group	$P2_1/n$
a/Å	21.4330(5)
b/Å	10.5389(2)
c/Å	24.3822(6)
α/°	90
β/°	113.594(3)
γ/°	90
V/$Å^3$	5047.1(2)
Z	4
Z'	1
Wavelength/Å	0.71073
Radiation type	Mo K_α
Θ_{min}/°	6.628
Θ_{max}/°	51.36
Measured Refl's.	118293
Indep't Refl's	9568

Refl's I≥2 σ(I)	0.0515
R_{int}	0.0634
Parameters	561
Restraints	0
Largest Peak	0.4012
Deepest Hole	-0.3498
GooF	1.071
wR_2 (all data)	0.1065
wR_2	0.1022
R_1 (all data)	0.0616
R_1	0.0515

Structure Quality Indicators

Reflections:	d min (Mo) 2Θ=51.4° 0.82	I/σ(I) 33.9	Rint 5.59%	Full 50.5° 99.8
Refinement:	Shift 0.001	Max Peak 0.4	Min Peak -0.3	GooF 1.071

A red plate-shaped-shaped crystal with dimensions 0.36 × 0.22 × 0.17 mm^3 was mounted on a mitegen tip in Paratone oil. Data were collected using an Xcalibur, Eos diffractometer equipped with an Oxford Cryosystems Cryostream 700+ low-temperature device operating at T = 120.0 K.

Data were measured using ω scans with Mo K_α radiation. The diffraction pattern was indexed and the total number of runs and images was based on the strategy calculation from the program CrysAlisPro 1.171.41.123a (Rigaku OD, 2022). The maximum resolution that was achieved was Θ = 51.36° (0.82 Å).

The unit cell was refined using CrysAlisPro 1.171.41.123a (Rigaku OD, 2022) on 9568 reflections, 8% of the observed reflections.

Data reduction, scaling and absorption corrections were performed using CrysAlisPro 1.171.41.123a (Rigaku OD, 2022). The final completeness is 99.8 % out to 51.36° in Θ. A multi-scan absorption correction was performed using CrysAlisPro 1.171.41.123a (Rigaku Oxford Diffraction, 2022) Empirical absorption correction using spherical harmonics, implemented in SCALE3 ABSPACK scaling algorithm. The absorption coefficient μ of this material is 0.099 mm^{-1} at this wavelength (λ = 0.71073 Å) and the minimum and maximum transmissions are 0.986 and 0.986.

The structure was solved and the space group $P2_1/n$ (# 14) determined by the ShelXT 2018/2 (Sheldrick, 2018) structure solution program using dual methods and refined by full matrix least squares minimisation on $\boldsymbol{F^2}$ using version 2018/3 of **ShelXL** 2018/3 (Sheldrick, 2015). All non-hydrogen atoms were refined anisotropically. Hydrogen atom positions were calculated geometrically and refined using the riding model. Hydrogen atom positions were calculated geometrically and refined using the riding model.

There is a single molecule in the asymmetric unit, which is represented by the reported sum formula. In other words: Z is 4 and Z' is 1

7. NMR Spectra

7.1 Compound 1

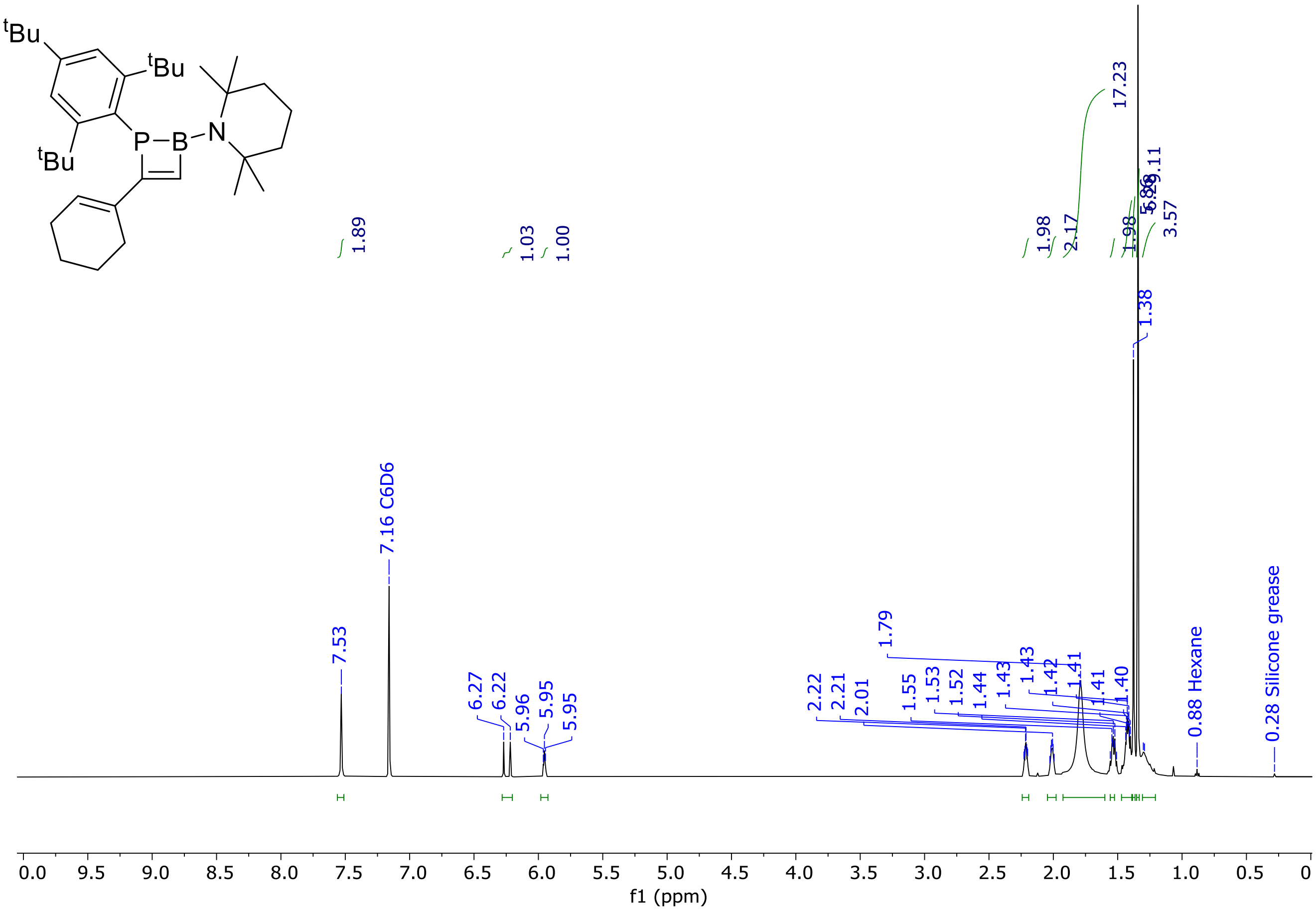

^{1}H NMR spectrum of 1 (500.2 MHz, 300 K, C_6D_6)

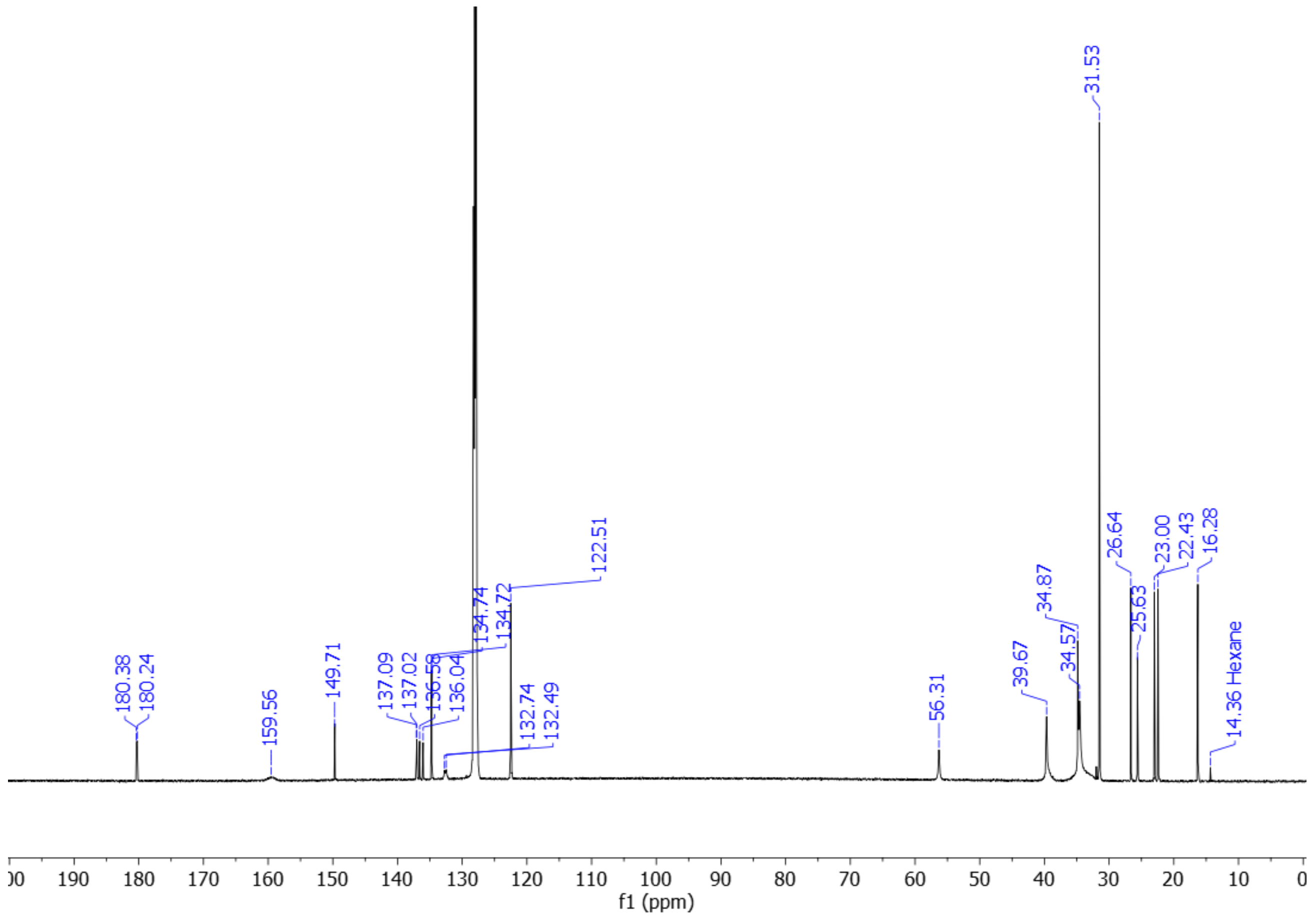

$^{13}C\{^{1}H\}$ NMR spectrum of 1 (125.8 MHz, 300 K, C_6D_6)

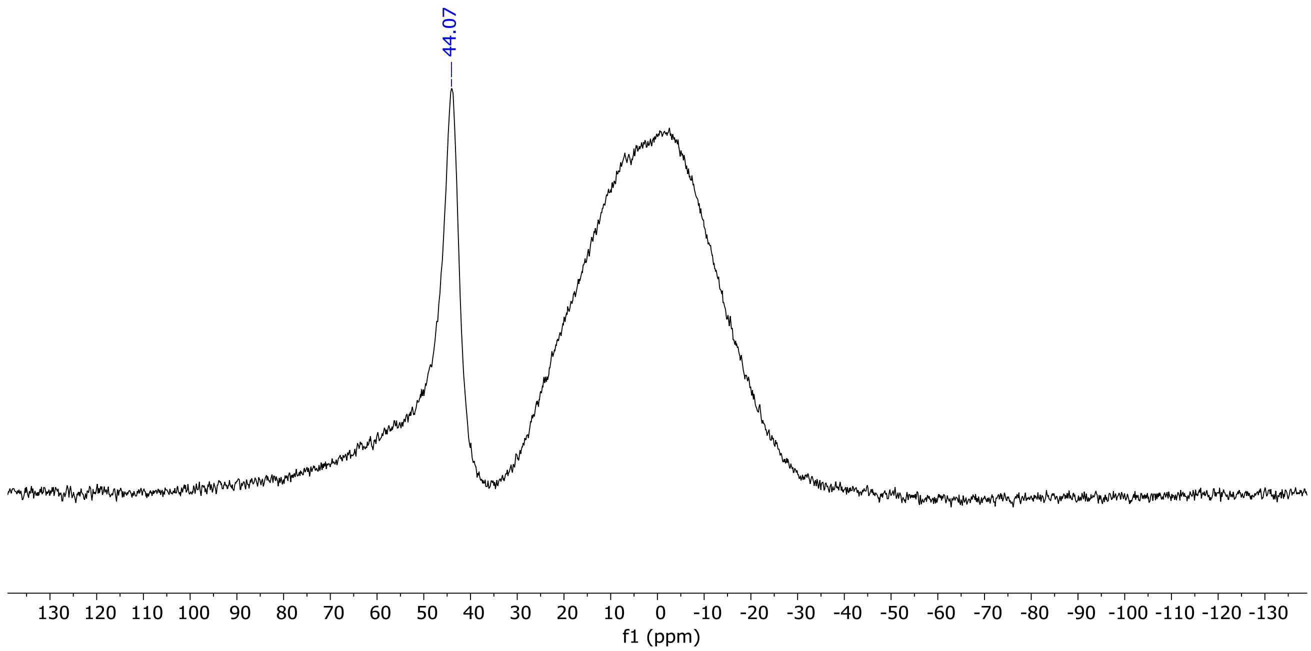

$^{11}B\{^{1}H\}$ NMR spectrum of 1 (160.5 MHz, 300 K, C_6D_6)

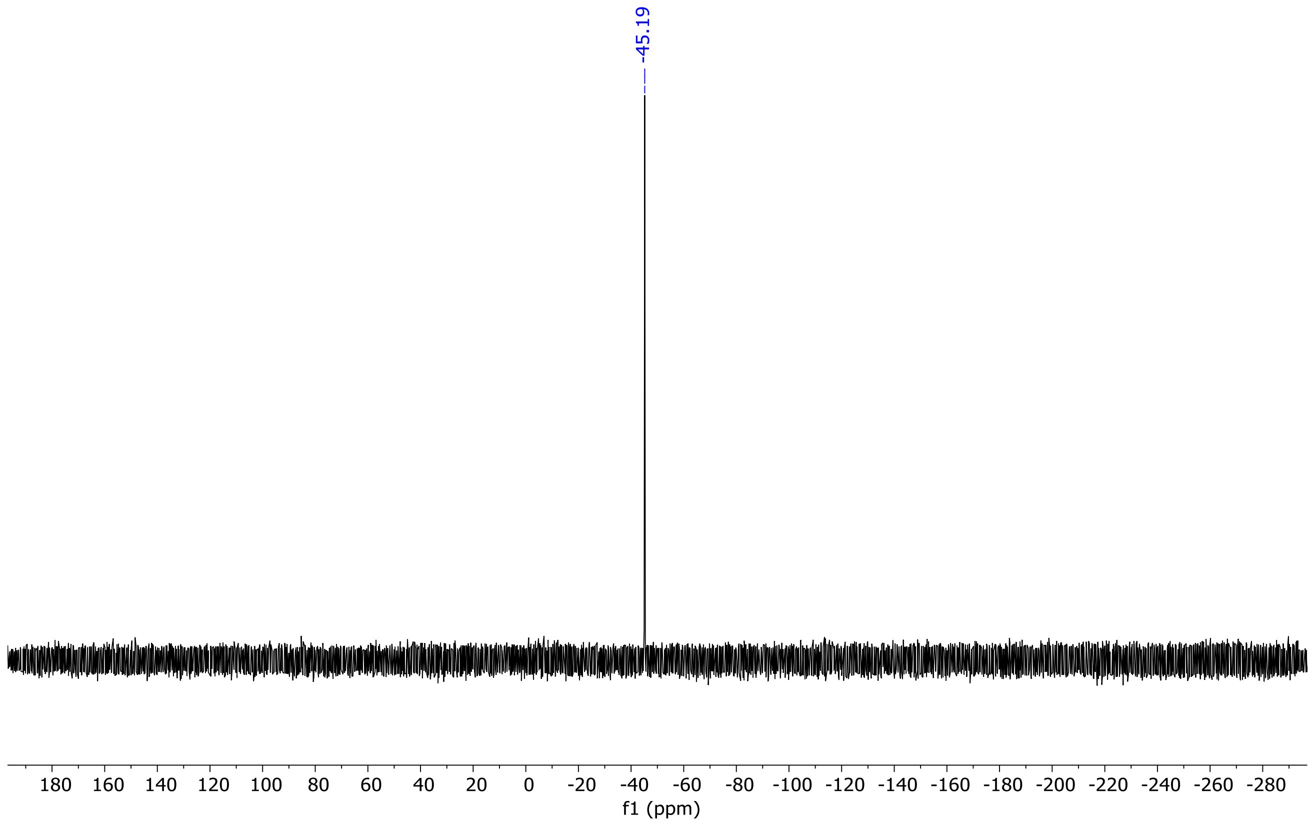

$^{31}P\{^{1}H\}$ NMR spectrum of 1 (202.5 MHz, 300 K, C_6D_6)

7.2 Compound 2

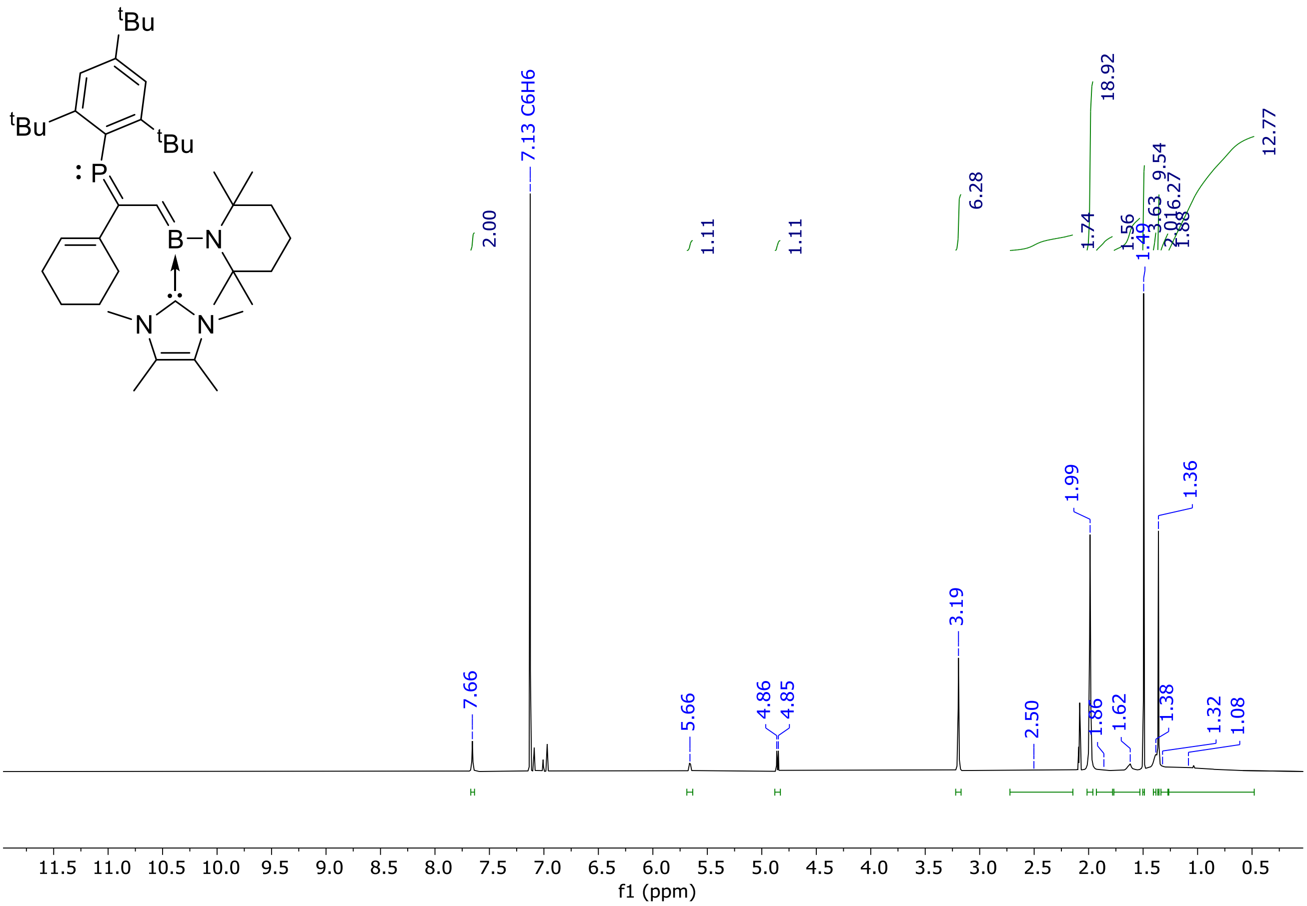

^{1}H NMR spectrum of 2 (500.2 MHz, 300 K, Toluene-d_8)

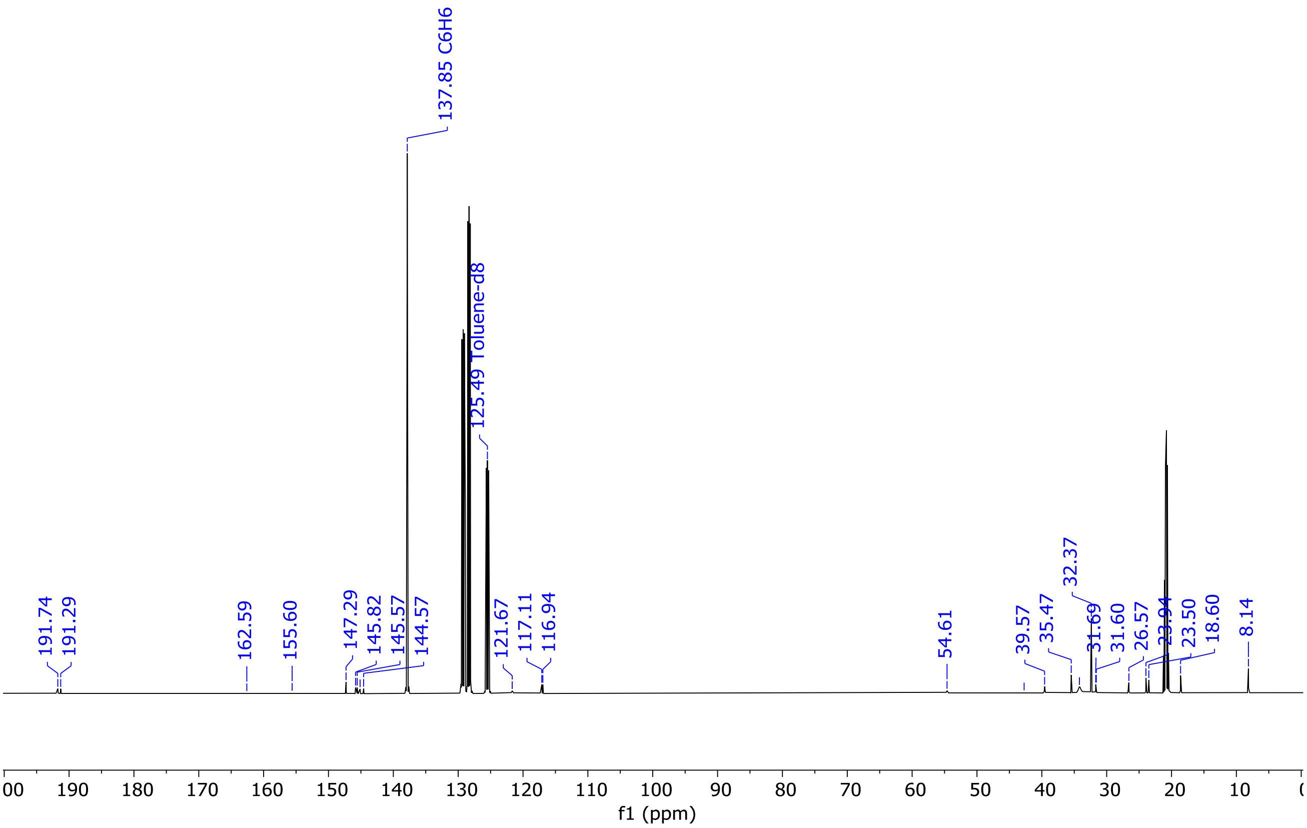

$^{13}C\{^1H\}$ **NMR spectrum of 2 (125.8 MHz, 300 K, Toluene-d_8)**

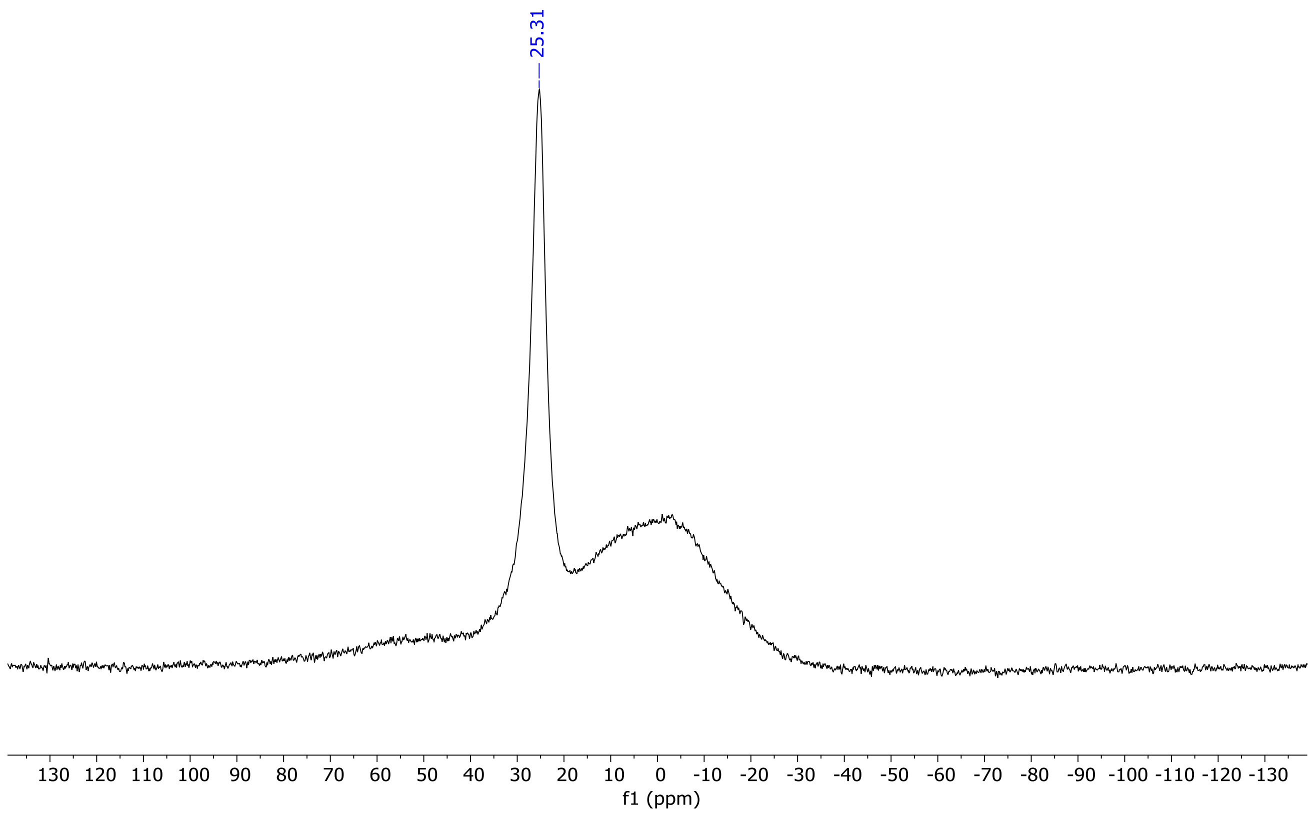

$^{11}B\{^{1}H\}$ NMR spectrum of 2 (160.5 MHz, 300 K, Toluene-d_8)

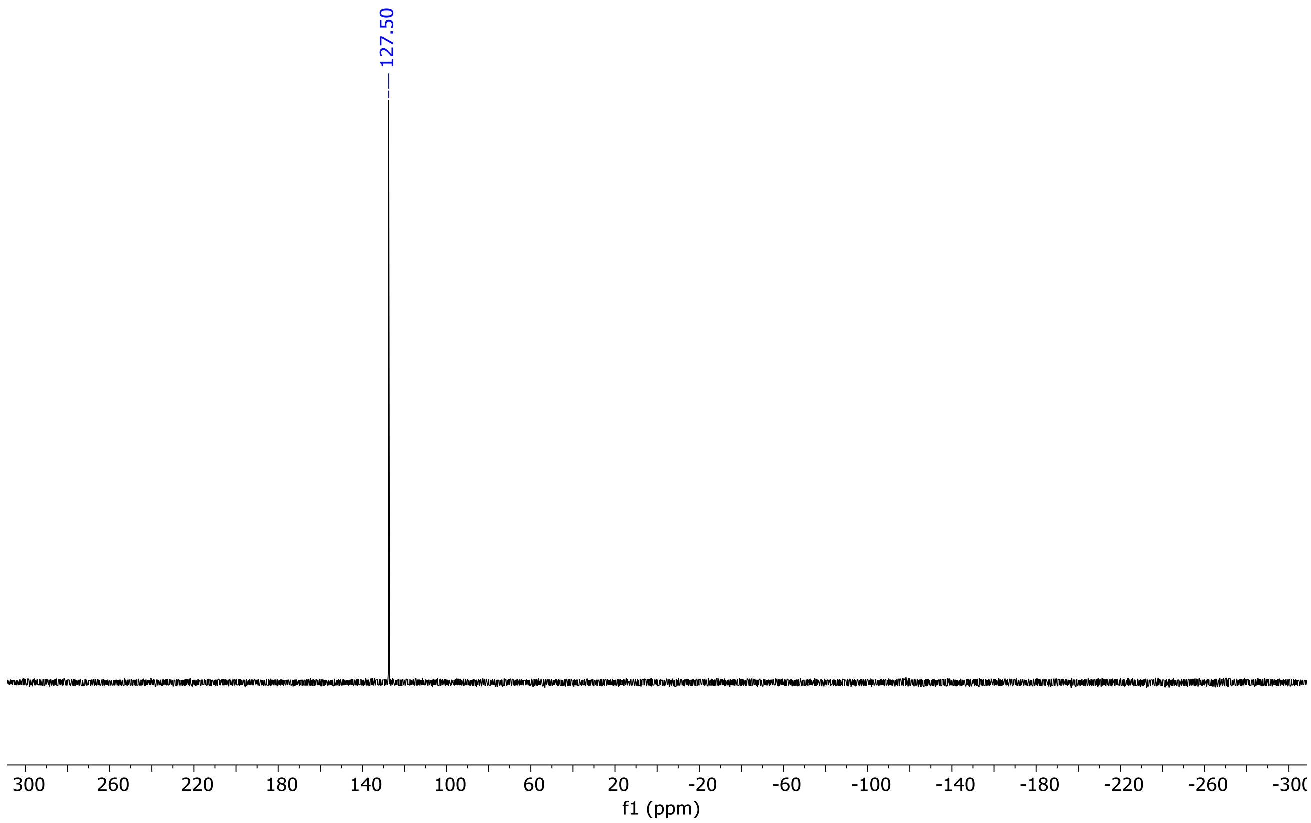

$^{31}P\{^{1}H\}$ NMR spectrum of 2 (202.5 MHz, 300 K, Toluene-d_8)

7.3 Compound 3

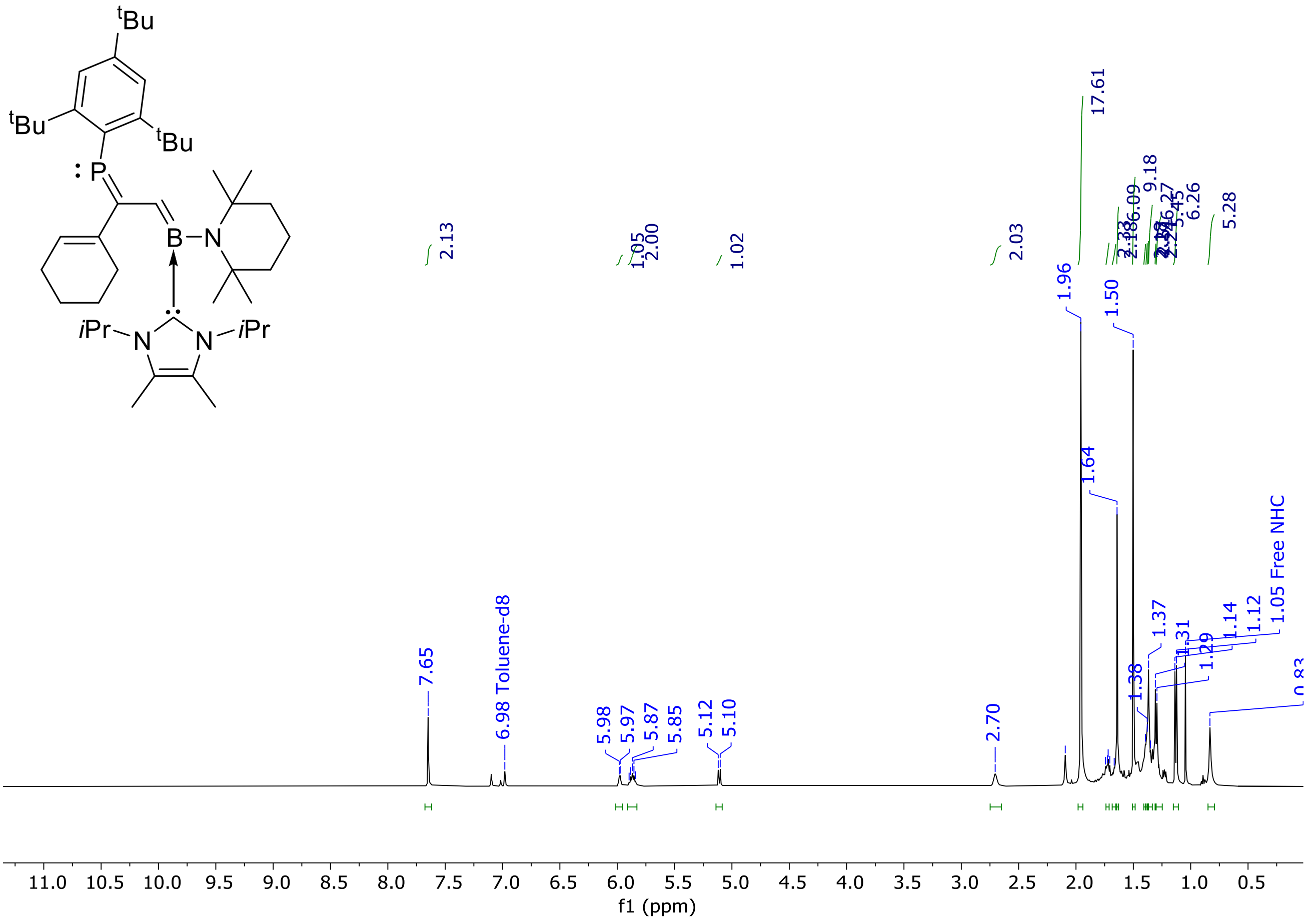

^{1}H NMR spectrum of 3 (500.2 MHz, 300 K, Toluene-d_8)

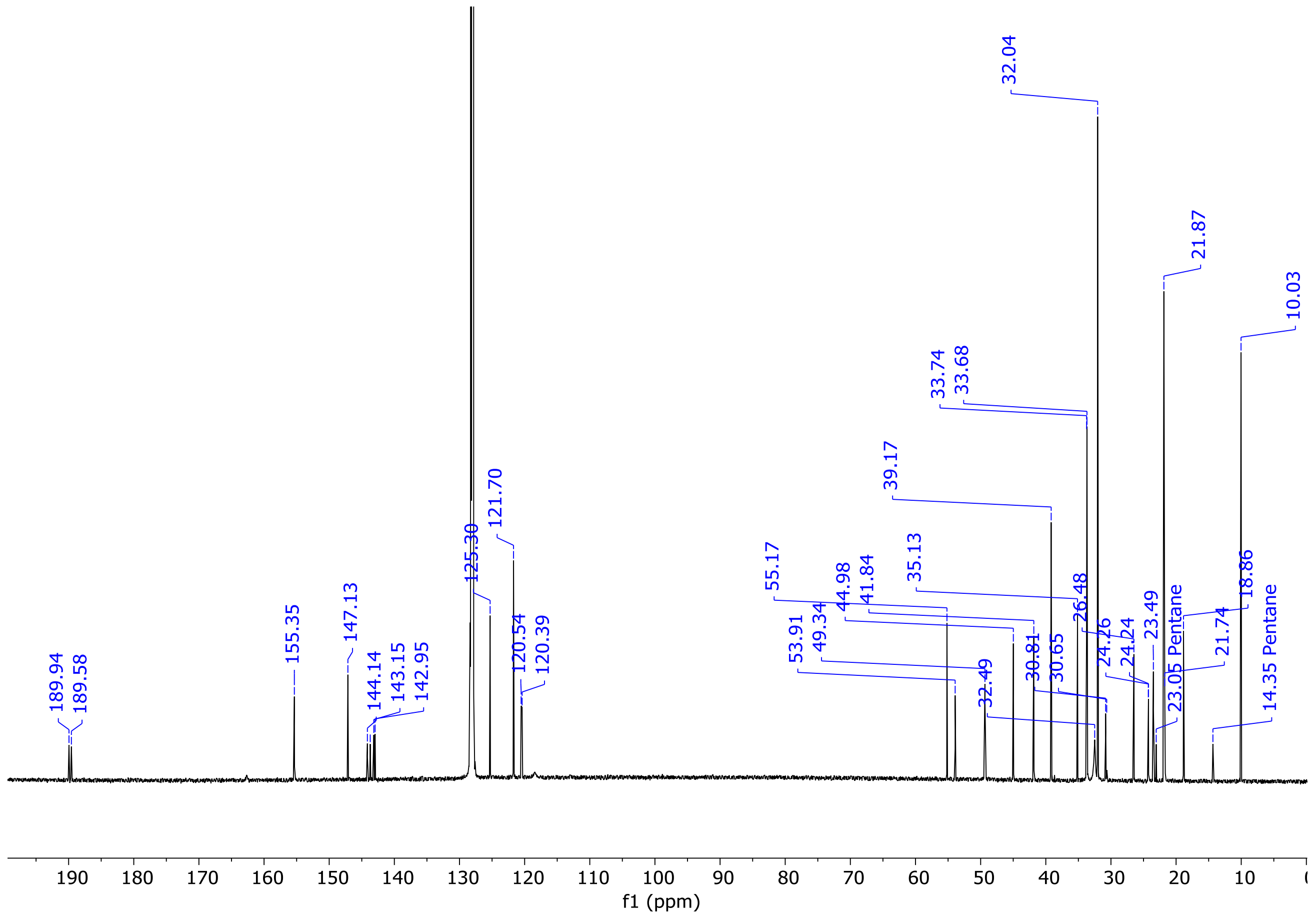

$^{13}C\{^1H\}$ NMR spectrum of 3 (125.8 MHz, 300 K, C_6D_6)

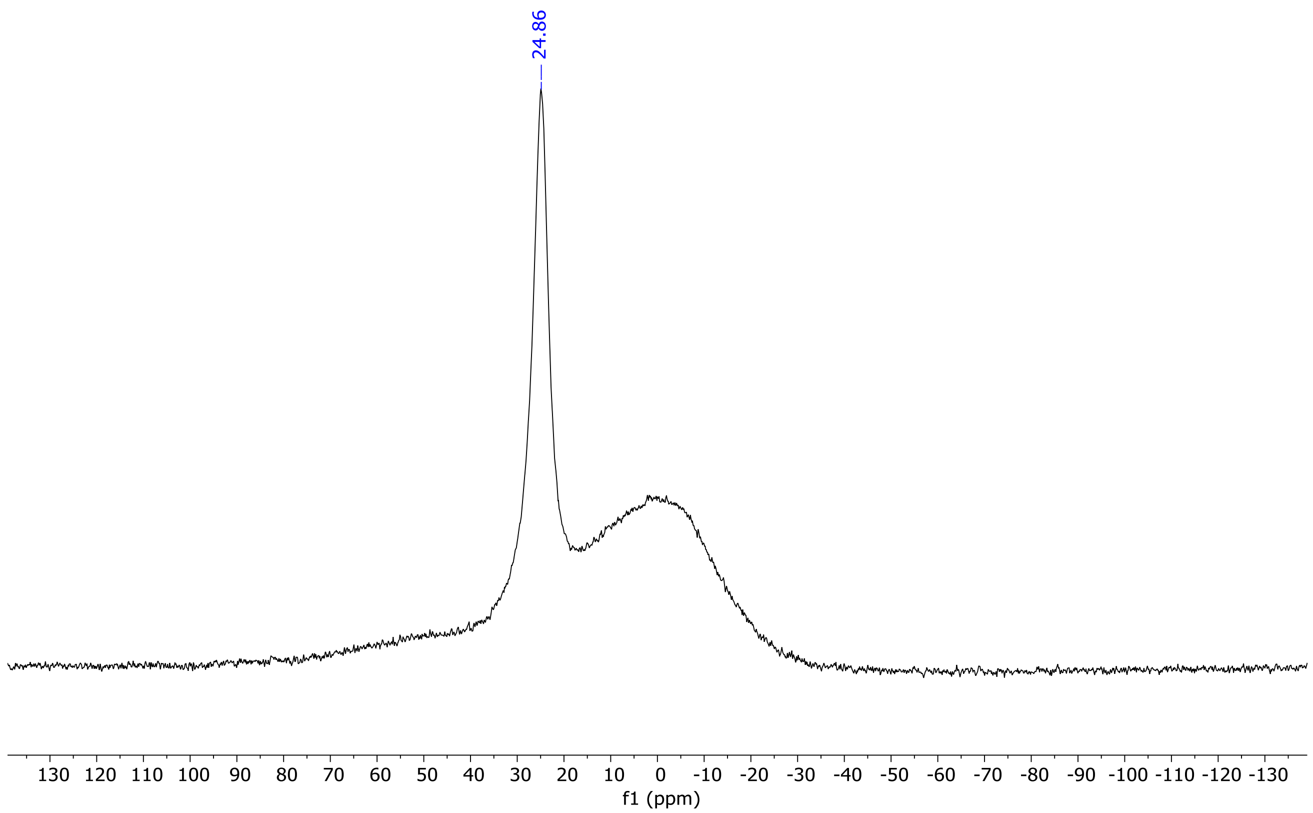

$^{11}B\{^{1}H\}$ NMR spectrum of 3 (160.5 MHz, 300 K, C_6D_6)

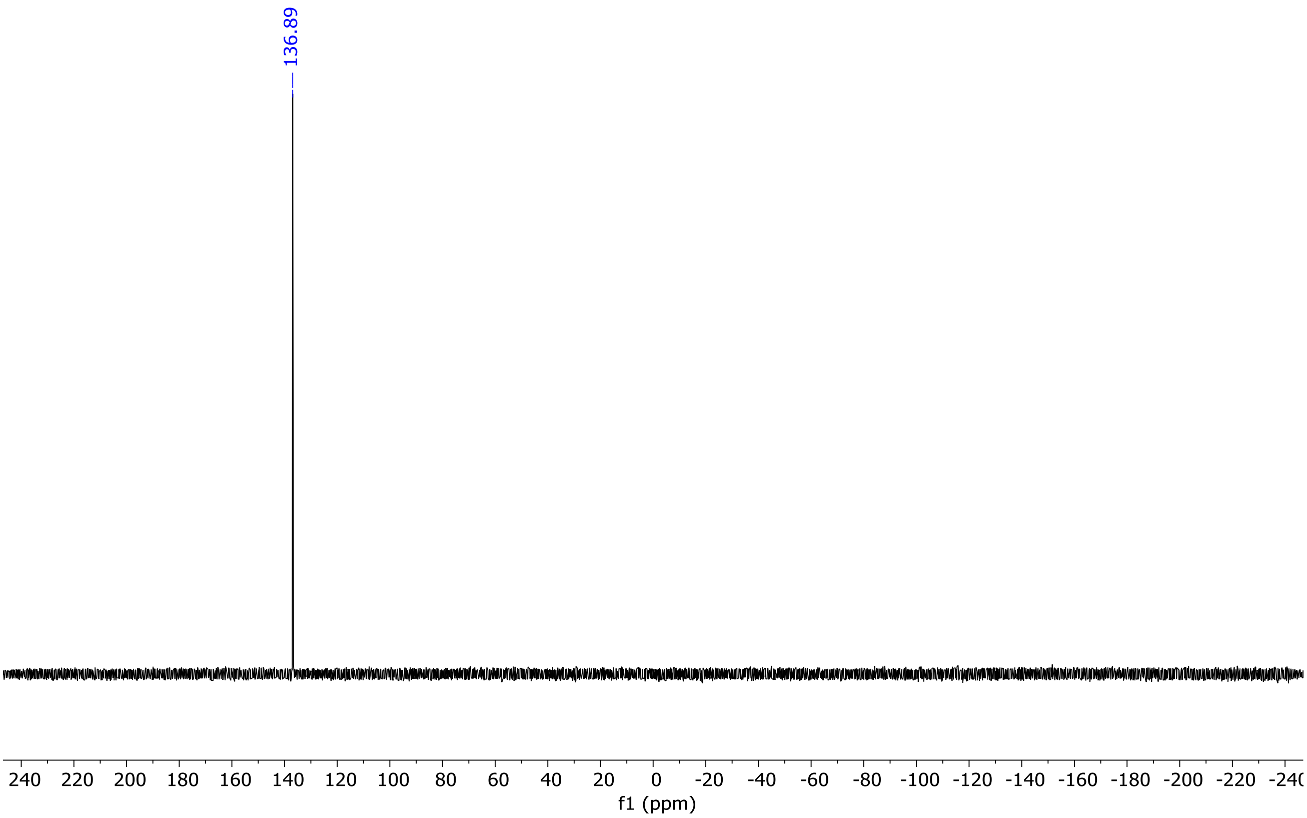

$^{31}P\{^1H\}$ NMR spectrum of 3 (202.5 MHz, 300 K, C_6D_6)

7.4 Compound 4

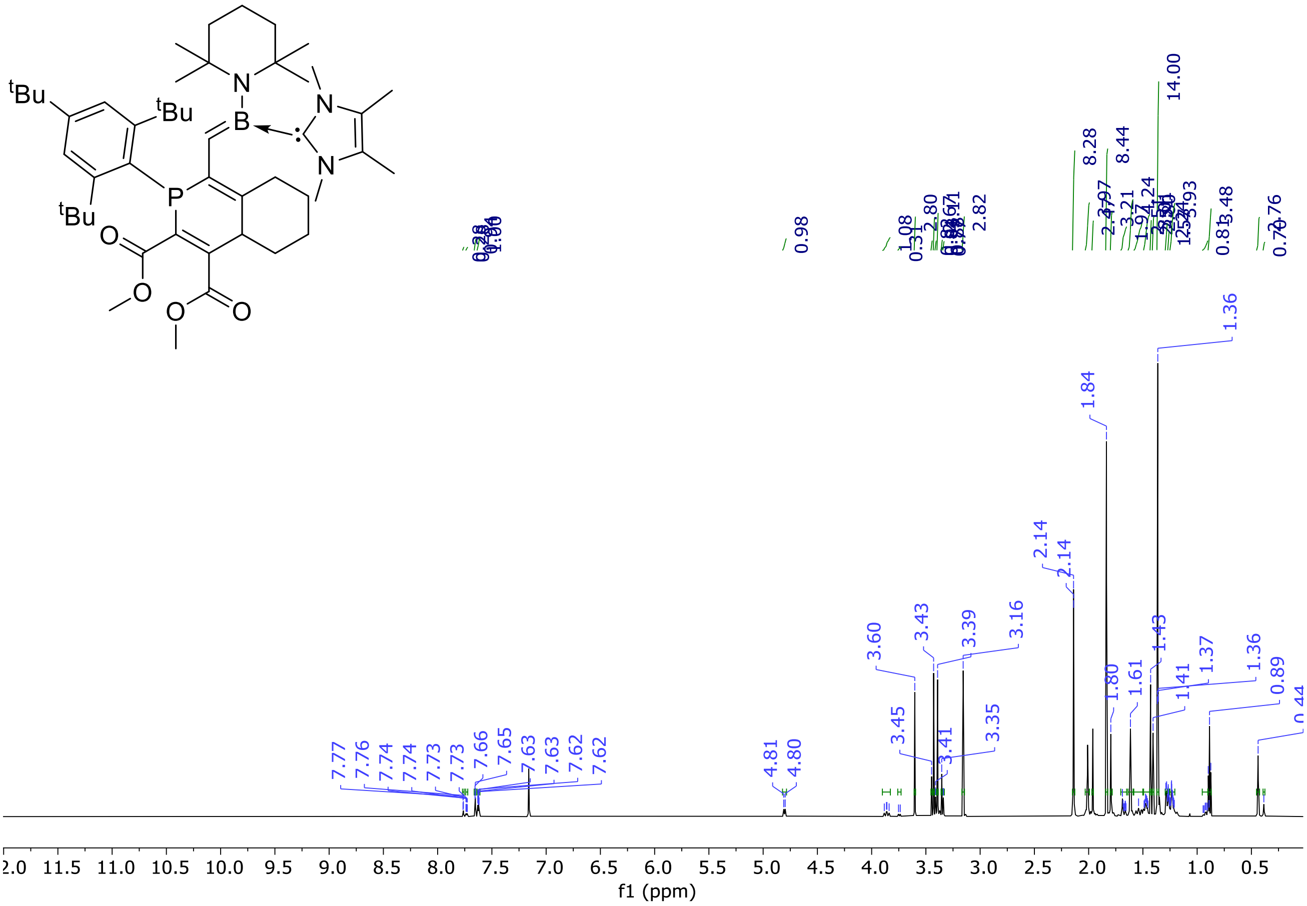

^{1}H NMR spectrum of 4 (500.2 MHz, 300 K, Toluene-d$_8$)

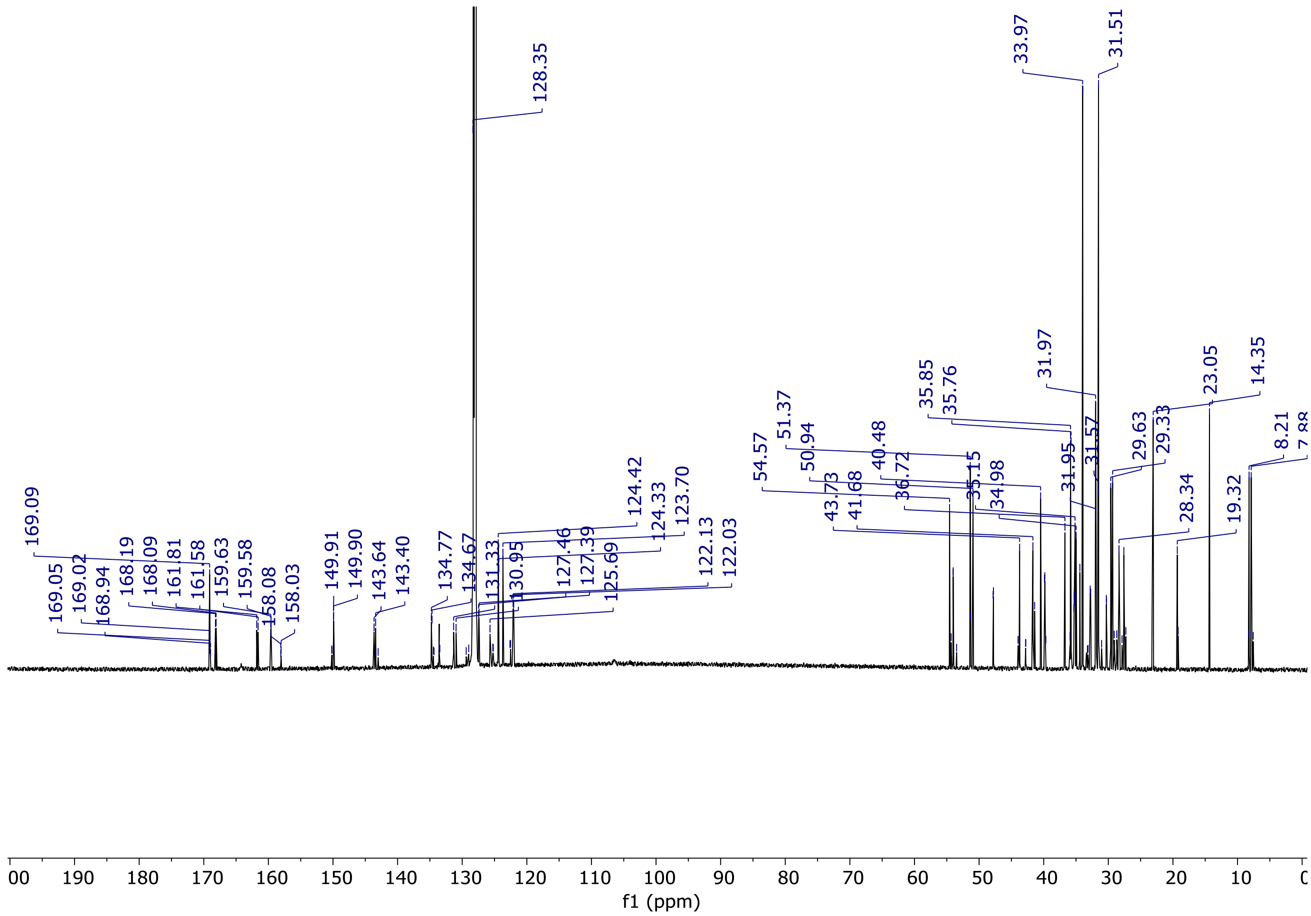

$^{13}C\{^1H\}$ NMR spectrum of 4 (125.8 MHz, 300 K, C_6D_6)

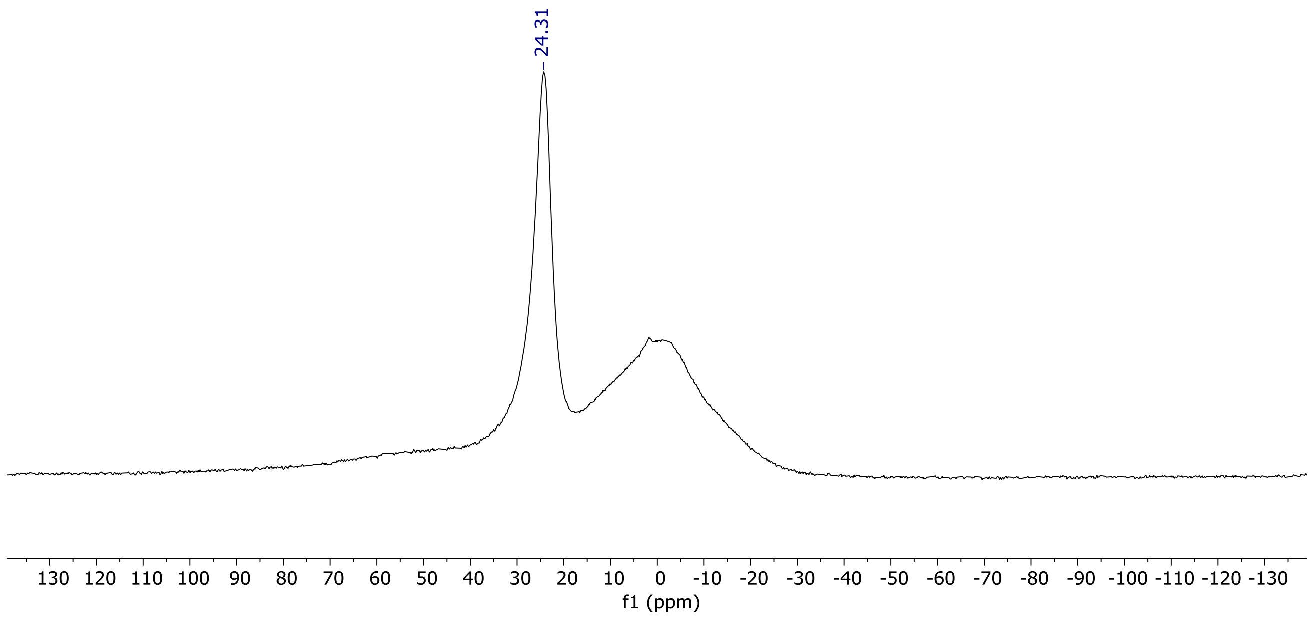

$^{11}B\{^1H\}$ NMR spectrum of 4 (160.5 MHz, 300 K, C_6D_6)

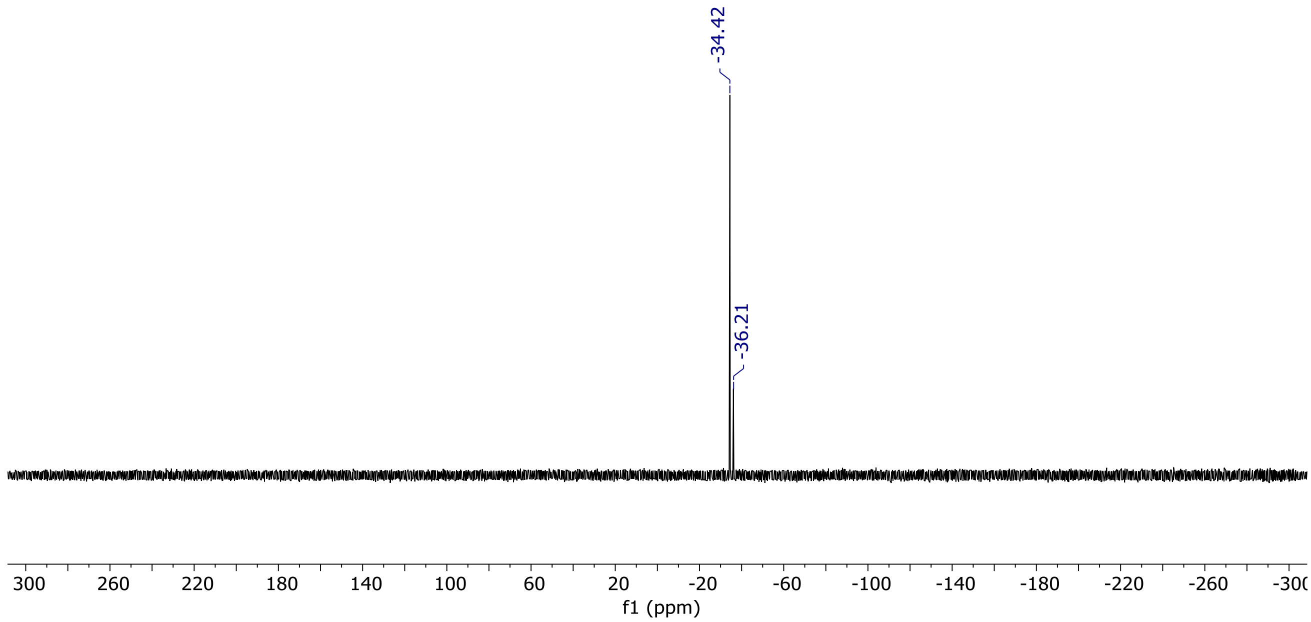

$^{31}P\{^1H\}$ NMR spectrum of 4 (202.5 MHz, 300 K, C_6D_6)

7.5 DMAP-supported Phosphabora[3]-dendralene

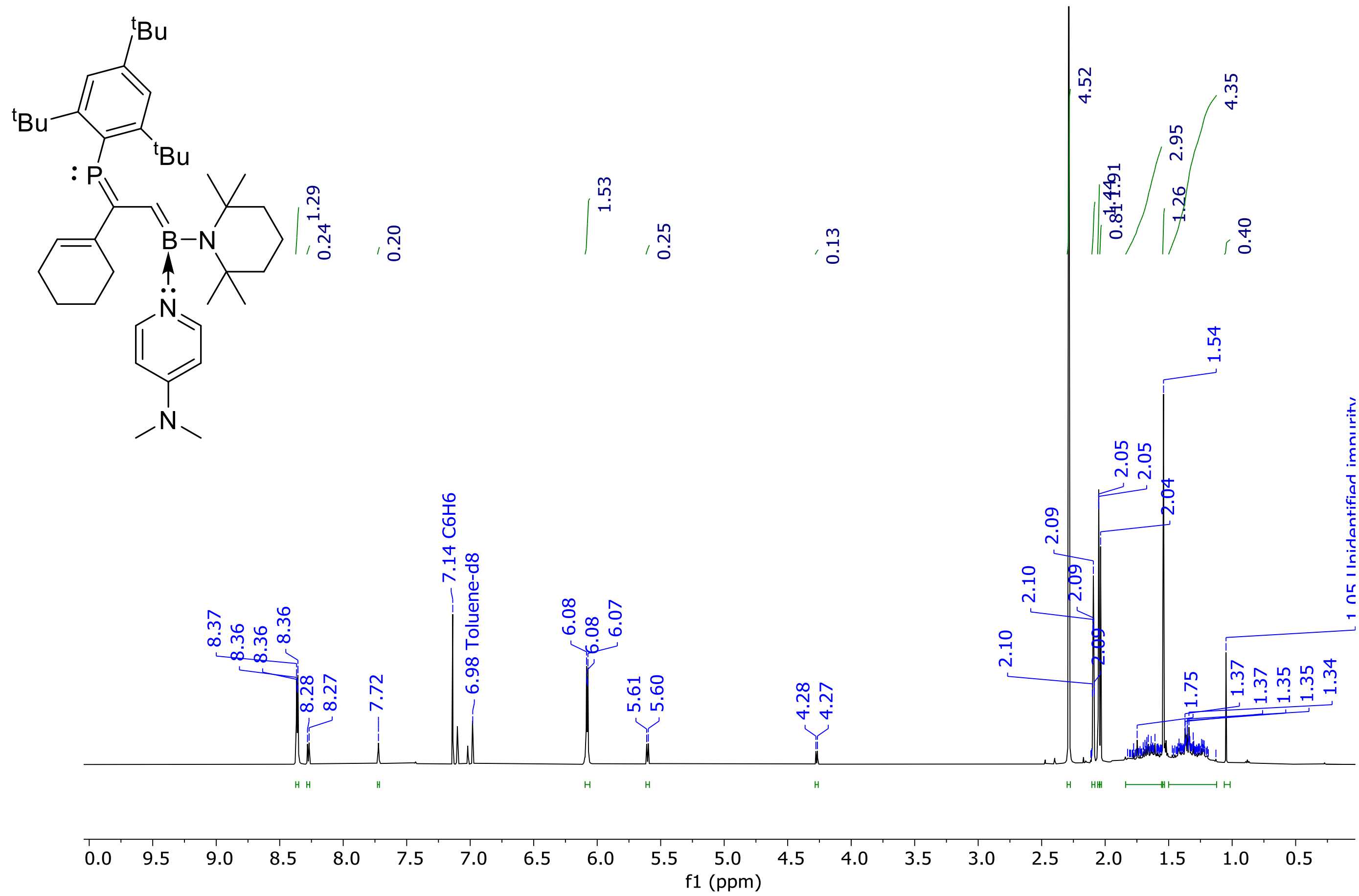

^{1}H NMR spectrum of DMAP-supported phosphabora[3]-dendralene (500.2 MHz, 300 K, Toluene-d_8)

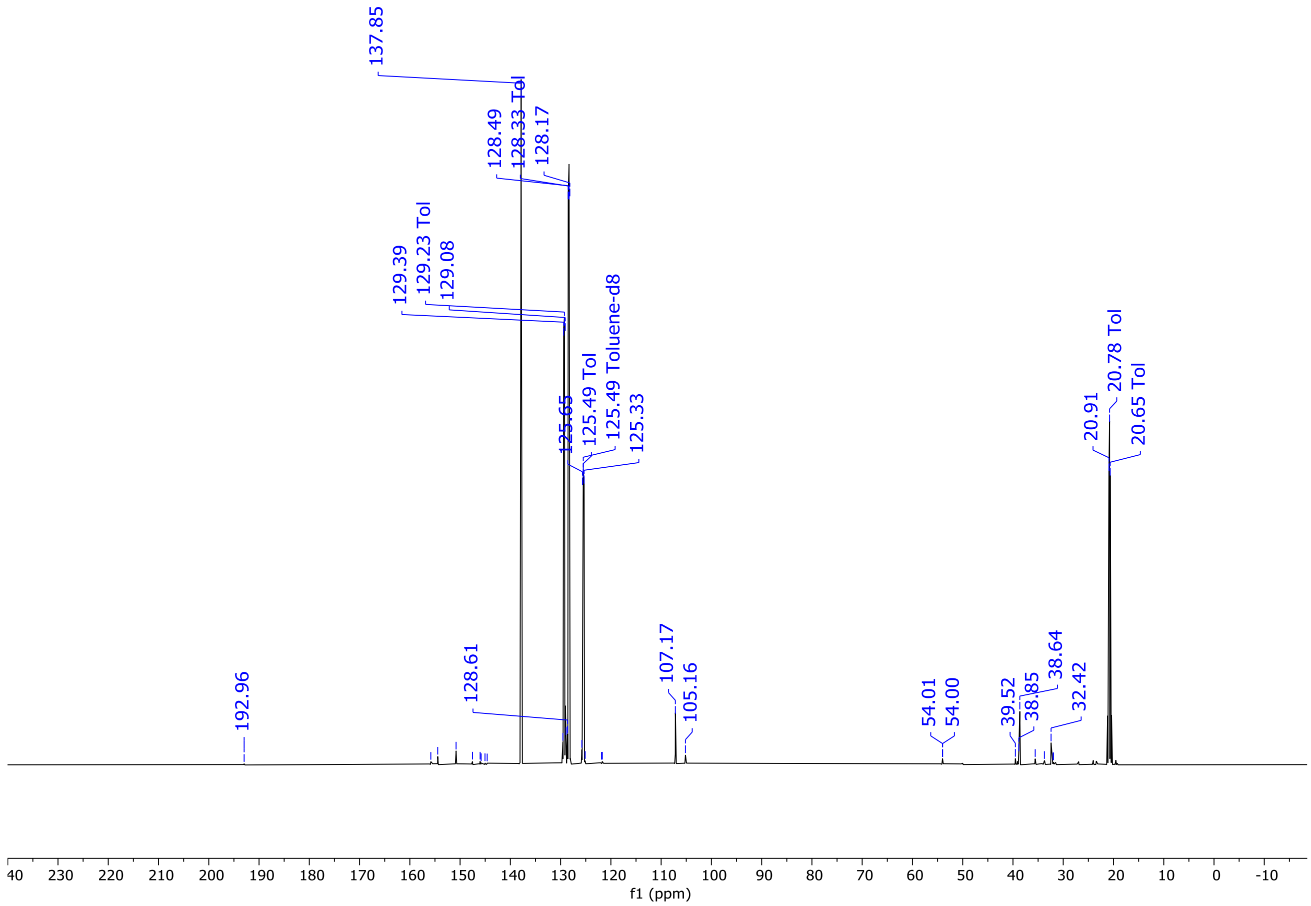

$^{13}C\{^{1}H\}$ NMR spectrum of 4 (125.8 MHz, 300 K, Toluene-d$_8$)

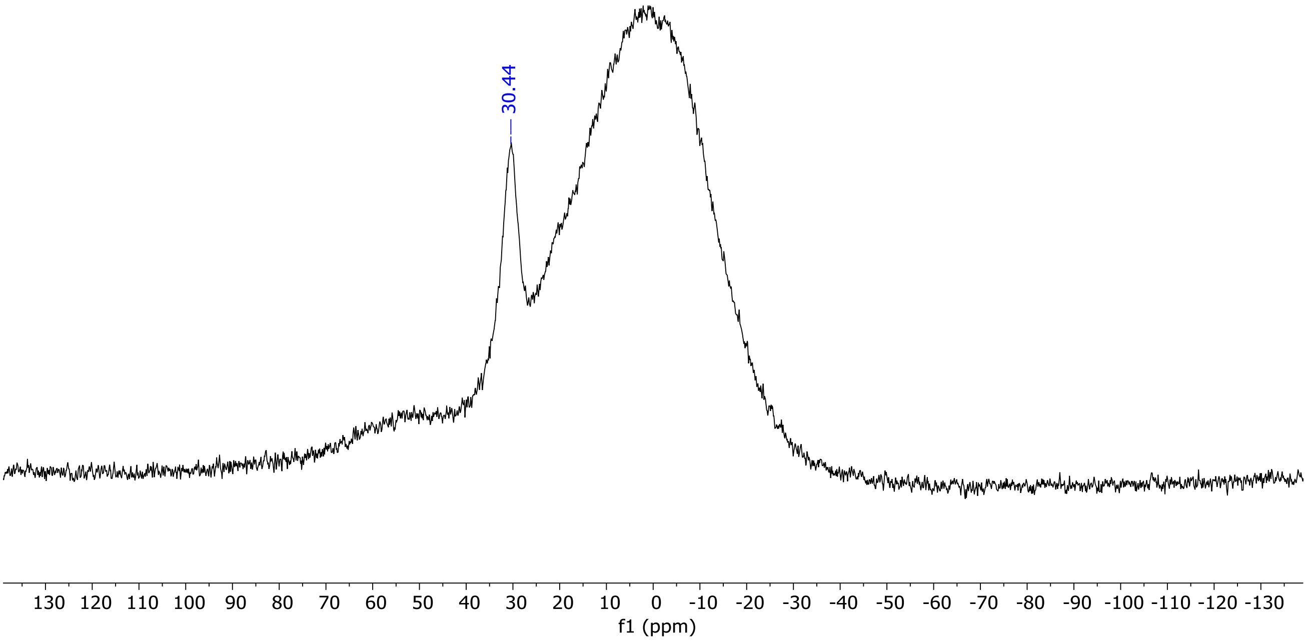

$^{11}B\{^{1}H\}$ NMR spectrum of DMAP-supported phosphabora[3]-dendralene (160.5 MHz, 300 K, Toluene-d_8)

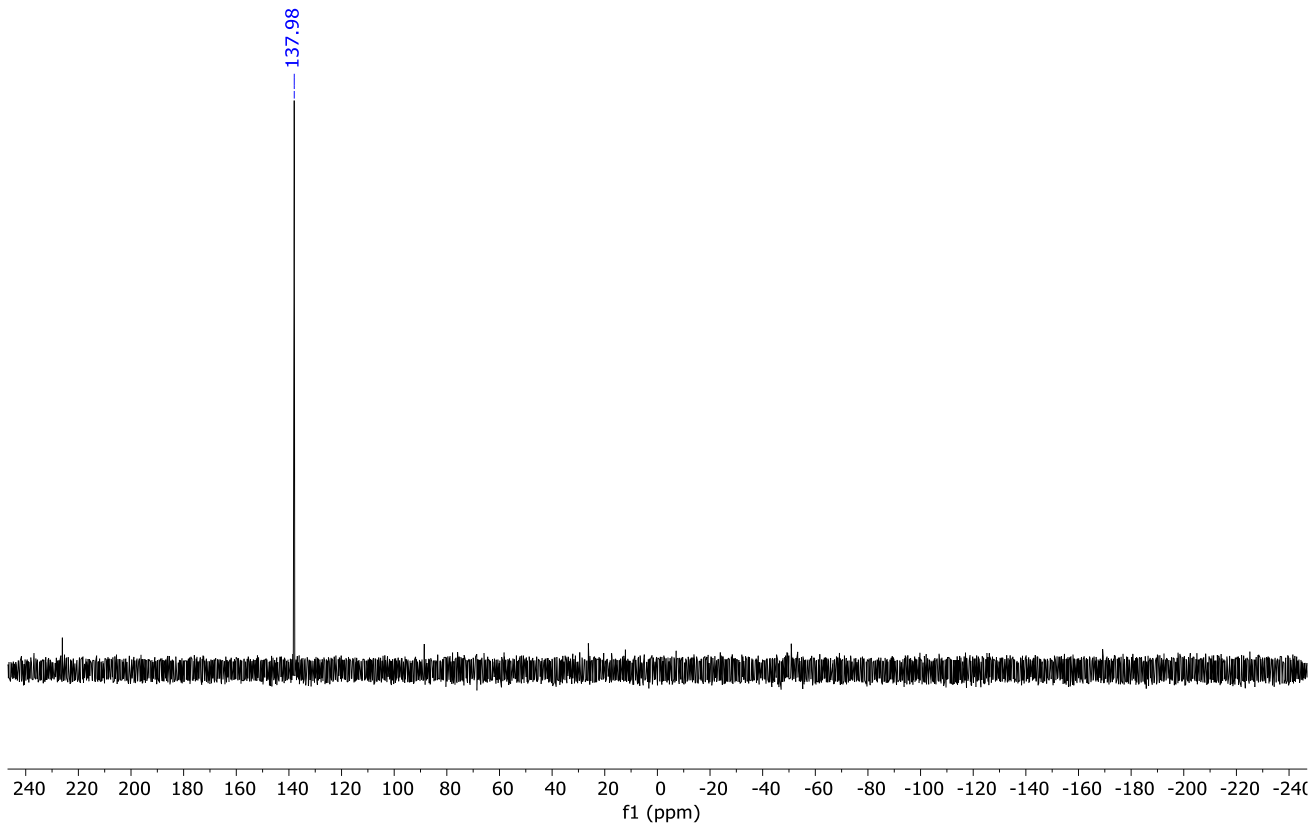

$^{31}P\{^{1}H\}$ NMR spectrum of DMAP-supported phosphabora[3]-dendralene (202.5 MHz, 300 K, Toluene-d_8)

8. References

(1) Cowley, A. H.; Norman, N. C.; Pakulski, M.; Becker, G.; Layh, M.; Kirchner, E.; Schmidt, M. Phosphorus Compounds Containing Sterically Demanding Groups. In *Inorganic Syntheses*; John Wiley & Sons, Ltd, 1990; pp 235–240. https://doi.org/10.1002/9780470132586.ch46.

(2) Purkait, T. K.; Press, E. M.; Marro, E. A.; Siegler, M. A.; Klausen, R. S. Low-Energy Electronic Transition in SiB Rings. *Organometallics* **2019**, *38* (8), 1688–1698. https://doi.org/10.1021/acs.organomet.8b00804.

(3) Borys, A. M.; Rice, E. F.; Nichol, G. S.; Cowley, M. J. The Phospha-Bora-Wittig Reaction. *J. Am. Chem. Soc.* **2021**, *143* (35), 14065–14070. https://doi.org/10.1021/jacs.1c06228.

(4) Kuhn, N.; Kratz, T. Synthesis of Imidazol-2-Ylidenes by Reduction of Imidazole-2(3H)-Thiones. *Synthesis* **1993**, *1993* (6), 561–562. https://doi.org/10.1055/s-1993-25902.

(5) Bell, R. A.; Saunders, J. K. Correlation of the Intramolecular Nuclear Overhauser Effect with Internuclear Distance. *Can. J. Chem.* **1970**, *48* (7), 1114–1122. https://doi.org/10.1139/v70-184.
